# Biosynthetic flexibility of *Pseudomonas aeruginosa* leads to hydroxylated 2-alkylquinolones with proinflammatory host response

**DOI:** 10.1038/s42004-023-00937-y

**Published:** 2023-07-03

**Authors:** Viktoriia Savchenko, Dávid Szamosvári, Yifan Bao, Marc Pignitter, Thomas Böttcher

**Affiliations:** 1 Faculty of Chemistry, Institute for Biological Chemistry & Centre for Microbiology and Environmental Systems Science, Department of Microbiology and Ecosystems Science, University of Vienna, Josef-Holaubek-Platz 2 (UZA II), 1090 Vienna, Austria; 2 Vienna Doctoral School in Chemistry (DoSChem), University of Vienna, Währinger Str. 42, 1090 Vienna, Austria; 3 Faculty of Chemistry, Institute for Physiological Chemistry, University of Vienna, Josef-Holaubek-Platz 2 (UZA II), 1090 Vienna, Austria

**Keywords:** Small molecules, Biosynthesis, Natural products, Natural product synthesis, Mass spectrometry

## Abstract

The human pathogen *Pseudomonas aeruginosa* produces various 4(1*H*)-quinolones with diverse functions. Among these, 2-nonyl-4(1*H*)-quinolone (NQ) and its *N*-oxide (NQNO) belong to the main metabolites. Their biosynthesis involves substrates from the fatty acid metabolism and we hypothesized that oxidized fatty acids could be responsible for a so far undetected class of metabolites. We developed a divergent synthesis strategy for 2′-hydroxy (2′-OH) and 2′-oxo- substituted quinolones and *N*-oxides and demonstrated for the first time that 2′-OH-NQ and 2′-OH-NQNO but not the corresponding 2′-oxo compounds are naturally produced by PAO1 and PA14 strains of *P*. *aeruginosa*. The main metabolite 2′-OH-NQ is produced even in concentrations comparable to NQ. Exogenous availability of β-hydroxydecanoic acid can further increase the production of 2′-OH-NQ. In contrast to NQ, 2′-OH-NQ potently induced the cytokine IL-8 in a human cell line at 100 nм, suggesting a potential role in host immune modulation.

## Abstract (web summary)

4(1*H*)-Quinolones are widely present in human pathogen *Pseudomonas aeruginosa* with large structural and functional diversities, however, only limited congeners have been unambiguously identified so far. Here, the authors report hydroxy-2-alkylquinolone congeners from the strain *P. aeruginosa* by biosynthetic analysis as well as total synthesis, and reveal their function as cytokine IL-8 inducers.

## Introduction

**Fig. 1:**
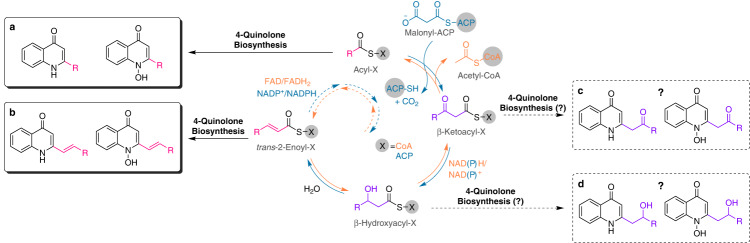
Fatty acid biosynthesis (cyan arrows) and fatty acid β-oxidation (orange arrows) in *P. aeruginosa* with known (black arrows) and hypothetical (dashed black arrows) substrate incorporation in 4-quinolone biosynthesis. **a** 2-alkyl-4(1*H*)-quinolones (AQs) and 2-alkyl-4(1*H*)-quinolone *N*-oxides (AQNOs); **b** Δ^1^-AQs and Δ^1^-AQNOs; **c** 2′-oxo-AQs and 2′-oxo-AQNOs; **d** 2′-OH-AQs and 2′-OH-AQNOs.

The compound classes of 2-alkyl-4(1*H*)-quinolones (AQs) and 2-alkyl-4(1*H*)-quinolone *N*-oxides (AQNOs) are produced most notably by the opportunistic human pathogen *Pseudomonas aeruginosa*, which is one of the major pathogens involved in the infection of the lungs of cystic fibrosis patients and other body sites^1–3^. While AQs, like 2-heptyl-4-hydroxyquinoline (HHQ), are known to act as quorum sensing molecules in *P. aeruginosa*^4,5^, AQNOs were found to exhibit a profound antibiotic activity against Gram-positive bacteria like *Staphylococcus* *aureus* including MRSA with the *trans*-unsaturated 2-nonenyl-4(1*H*)-quinolone *N*-oxide (Δ^1^-NQNO) as its most potent member^6^. AQs and AQNOs are characterized by a great diversity in length and saturation of their alkyl side chains, which may correlate with functional specialization in their biological activities. In *P. aeruginosa* alone, over 50 different 2-alkyl-4(1*H*)-quinolone compounds were already identified^7^. This variety is likely a result of the rather broad substrate specificity of the β-ketoacyl-(acyl-carrier-protein) synthase III (FabH)-like enzymes PqsB and PqsC. The heterodimeric PqsBC complex uses CoA-activated fatty acids to catalyze a decarboxylative Claisen-condensation of acyl-ACP and 2-aminobenzoylacetate (2-ABA) or 2-hydroxy-aminobenzoylacetate (2-HABA) to form AQs or AQNOs, respectively^8,9^. The fatty acids used in the biosynthesis of AQs and AQNOs may be provided by the bacterial fatty acid synthesis, β-oxidation, or be utilized from the medium (Fig. 1). Surprisingly, only saturated and unsaturated AQs and AQNOs have been unambigously identified so far^6,7^. We here aim to investigate if β-hydroxy fatty acids and β-oxo fatty acids of the fatty acid metabolism could also be utilized as substrates for the AQ and AQNO biosynthesis and potentially yield 2′-hydroxy-2-alkyl-4(1*H*)-quinolones (2′-OH-AQs) and 2′-oxo-2-alkyl-4(1*H*)-quinolones (2′-oxo-AQs) as well as their corresponding *N*-oxides (Fig. 1c, d). The discovery of these molecules would expand our understanding of the molecular arsenal of *P. aeruginosa* and allow to investigate their function in microbial competition and microbe host interactions.

In this work, we have generated synthetic standards confirming that 2′-OH-NQ and 2′-OH-NQNO but not the 2′-oxo compounds are produced by *P*. *aeruginosa*. 2′-OH-NQ was among the major 4(1*H*)-quinolones of *P*. *aeruginosa* and, in contrast to NQ, caused a potent stimulation of cytokine IL-8 in an epithelial cell line.

## Results and discussion

### Synthesis of quinolone standards

In order to investigate if 2′-OH-NQ and 2′-oxo-NQ and their corresponding *N*-oxides are produced by *P. aeruginosa*, we first aimed to prepare synthetic standards of these compounds. Several well established methods are known for the synthesis of saturated and unsaturated 2-alkyl and 2-alkenyl-4(1*H*)-quinolones and their *N*-oxides^10^. However, the key step of the cyclization requires relatively harsh conditions, which are not compatible with more sensitive functional groups. We thus aimed for a strategy to directly generate functionalized AQs and AQNOs. Sicker et al. reported 1987 the cyclization of 2′-nitrocinnamoyl derivatives to 2-methyl-4(1*H*)-quinolone *N-*oxides through a Pt(OH)_2_ catalyzed hydrogenation reaction under acidic conditions at room temperature^11^. In 2001, Jung et al. used a similar nitro-substrate for the reductive cyclization with Pd/C under neutral conditions to obtain 2-substituted 3-carboxyethylester-4(1*H*)-quinolone *N*-oxides^12^. Inspired by these earlier works, we developed a divergent reductive cyclization that allows the controlled synthesis of AQs or AQNOs, depending on the choice of catalyst and reaction time. Its mild reaction conditions appeared highly suitable for the synthesis of our more functionalized target compounds **1g,** **1h,** **2g** and **2h**.

**Fig. 2:**
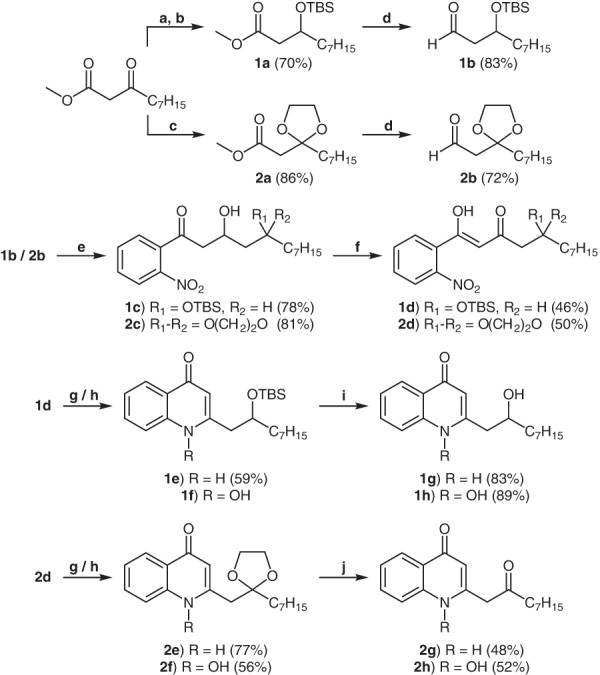
Synthesis of 2′-OH-NQ (**1g**), 2′-oxo-NQ (**2g**), and their corresponding *N*-oxides (**1h** and **2h**). Reaction conditions: **a** NaBH_4_, MeOH, rt, 1 h; **b** TBS-Cl, imidazole, DMF, 0 °C, o/n; **c** trimethylorthoformate, pTsOH, ethylene glycol, rt, 5 h; **d** DIBAL-H, DCM/toluene, -78 °C, 2 h; **e** 2-nitro-acetophenone, Bu_2_BOTf, DIPEA, DCM, 40 min at 0 °C, 30 min at rt, 30 min at −78 °C, 2.5 h at 0 °C; **f** DMP, DCM, 1 h at 0 °C, 4 h at rt; **g** H_2_, 10% Pd/C, EtOH, rt, 24 h; **h** H_2_, 10% Pt/C, EtOH, rt, 3 h; **i** TBAF, THF, rt, 30 min; **j** 6 M HCl aq./THF (1:1), rt, 24 h.

We started our synthesis by using methyl 3-oxodecanoate as common precursor whose keto group was either reduced with NaBH_4_ and subsequently protected with TBS (**1a**) or directly protected as ketal (**2a**) (Fig. 2). After reduction of the methyl ester group with DIBAL-H, the protected decanal derivatives **1b** and **2b** were used in a Lewis-acid catalyzed Mukaiyama aldol-type reaction^13^ with 2′-nitroacetophenone to obtain the key intermediates **1c** and **2c** in good yields. To allow later cyclization, compounds **1c** and **2c** were oxidized to their respective β-keto-enol compounds **1d** and **2d** via Dess-Martin oxidation. Other oxidation reagents like Jones reagent or IBX failed to provide the desired compounds. With **1d** and **2d** in hand, we performed the cyclization to their respective 4(1*H*)-quinolones and 4(1*H*)-quinolone *N*-oxides under reductive conditions at neutral pH. Using Pd/C as catalyst, we observed the cyclization of **1d** and **2d** to the protected 4(1*H*)-quinolones **1e** and **2e** after 24 h. Shorter reaction times gave significantly less 4(1*H*)-quinolones with equal amounts of 4(1*H*)-quinolone *N*-oxides. Surprisingly, when using Pt/C for 2–3 h, exclusively 4(1*H*)-quinolone *N*-oxides **1f** and **2f** were obtained without simultaneous production of the respective 4(1*H*)-quinolones. Longer reaction times led to complex mixtures of products with 4(1*H*)-quinolones as side products complicating later separation. Finally, deprotection with TBAF (for **1e** and **1f**) or HCl (for **2e** and **2f**) gave the desired 2′-OH-NQ (**1g**), 2′-OH-NQNO (**1h**), 2′-oxo-NQ (**2g**) and 2′-oxo-NQNO (**2h**) in high purity after column chromatography or HPLC.

### Qualitative and quantitative analysis of metabolites produced by *Pseudomonas aeruginosa*

**Fig. 3:**
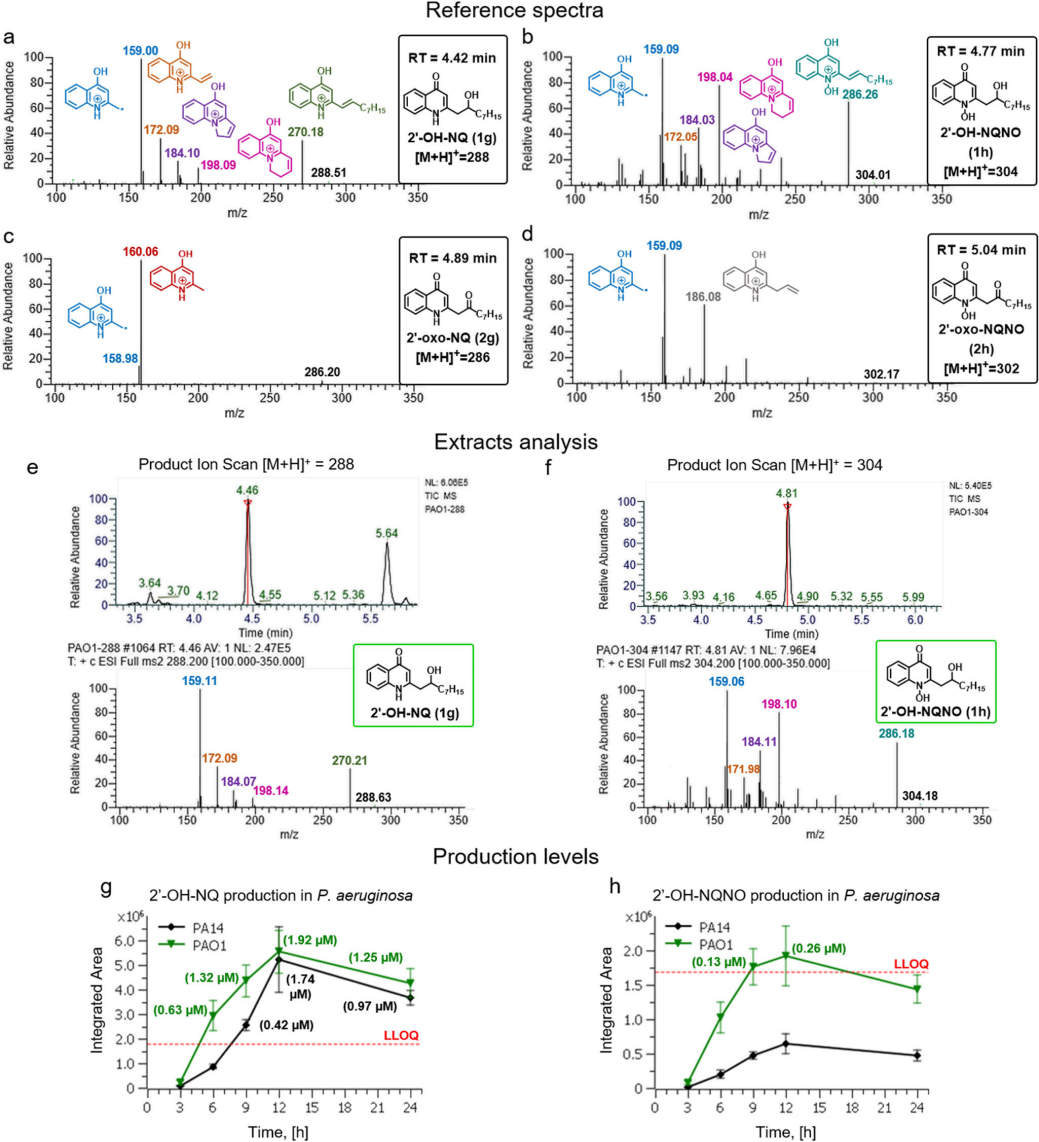
MS^2^ reference spectra with fragmentation of parent ions (product ion scan) of synthesized quinolones and analysis of extracted culture supernatants of *P. aeruginosa*. **a**–**d** MS^2^ fragmentation pattern and proposed structures of fragment ions of synthetic standards. **e**, **f** MS^2^ spectra of detected 2′-OH-NQ and 2′-OH-NQNO in extracts of *P. aeruginosa* culture supernatants. **g**, **h** Integrated area of monitored mass transitions of 2′-OH-NQ and 2′-OH-NQNO produced by *P. aeruginosa* PA14 (black) and *P. aeruginosa* PAO1 (green) during 24 h of incubation. Quantified concentrations are shown in brackets. LLOQ lower limit of quantification. Mean values are given for biological triplicates with the error bars representing the population standard deviation.

We subsequently used the synthesized quinolones as standards for the identification and quantification of 2′-hydroxy and 2′-oxo-4(1*H*)-quinolones and their *N*-oxides using LC–MS/MS (Fig. 3 and Table S3). Remarkably, the MS^2^ fragmentation pattern of the quasi-molecular ions of 2′-OH-NQ and 2′-OH-NQNO showed a unique fragment [M + H-18]^+^, which is most likely due to the elimination of water from the 2′-hydroxyalkyl side chain (Fig. 3a, b). Interestingly, all other detected fragments are typical for Δ^1^-unsaturated AQs and their *N*-oxides^7,14^, which supports the proposed structure of the unique [M + H-18]^+^ fragment.

While 2′-OH-NQ has the same parental mass as previously detected NQNO, our LC–MS/MS method allows us to confidently distinguish between these two isomeric quinolones via significantly different MS^2^ fragmentation and retention time (RT) (Table S3a, b). Moreover, we could exclude the possibility of the detection of isomeric quinolones with the different position of the hydroxylation in the side chain, as not only RT of natively produced quinolones fit our synthetic standards, but also the fragmentation pattern precisely matches (including fragment ions distribution) (Fig. 3a, b and Fig. 3e, f, see Supplementary Information MS^2^ Assignments).

In order to investigate the native production of the new quinolones via their retention times and unique fragmentation fingerprints, we selected *P. aeruginosa* strains PAO1 and PA14 and analyzed extracted culture supernatants. Additionally, we investigated culture supernatants of *Burkholderia thailandensis* and *Burkholderia ambifaria* AMMD, which are known to produce a large variety of 2-alkyl-4(1*H*)-quinolones^14,15^. Indeed, both strains of *P. aeruginosa* produced 2′-OH-NQ and 2′-OH-NQNO (Fig. 3e, f). In contrast, 2′-oxo-NQ and corresponding *N*-oxide were not detected at any time point during 24 h of incubation (Tables S8 and S9). Interestingly, none of the new quinolones were found in the two *Burkholderia* strains at any time or growth condition tested (Tables S10-S13). These results indicate that at least *P. aeruginosa* is able to produce the compound classes of 2′-hydroxy-4(1*H*)-quinolones and their *N*-oxides but not the corresponding 2′-oxo classes. In order to quantify the detected quinolones, mass transitions of the most intense or unique ions of the MS^2^ spectra were selected. External calibration was performed using concentration series of standard compounds (Table S2). *P. aeruginosa* strains produced 2′-OH-NQ and 2′-OH-NQNO while grown for 3-24 h, peaking at 12 h of incubation (Figs. 3g, h, S1, S2, and Tables S4, S5). NQ and NQNO were also quantified in this experiment for direct comparison with production levels of new quinolones (Tables S6 and S7). In both *P. aeruginosa* PAO1 and PA14 strains, 2′-OH-NQ was produced in higher concentrations (max. 0.55 mg L^−1^ or 1.9 µM) than the corresponding *N*-oxide (max. 0.08 mg L^−1^ or 0.3 µM), while maximum NQ and NQNO levels were 0.63 mg L^−1^ and 3.1 mg L^−1^, respectively.

### Insights into substrate utilization and biosynthesis of hydroxylated quinolones

**Fig. 4:**
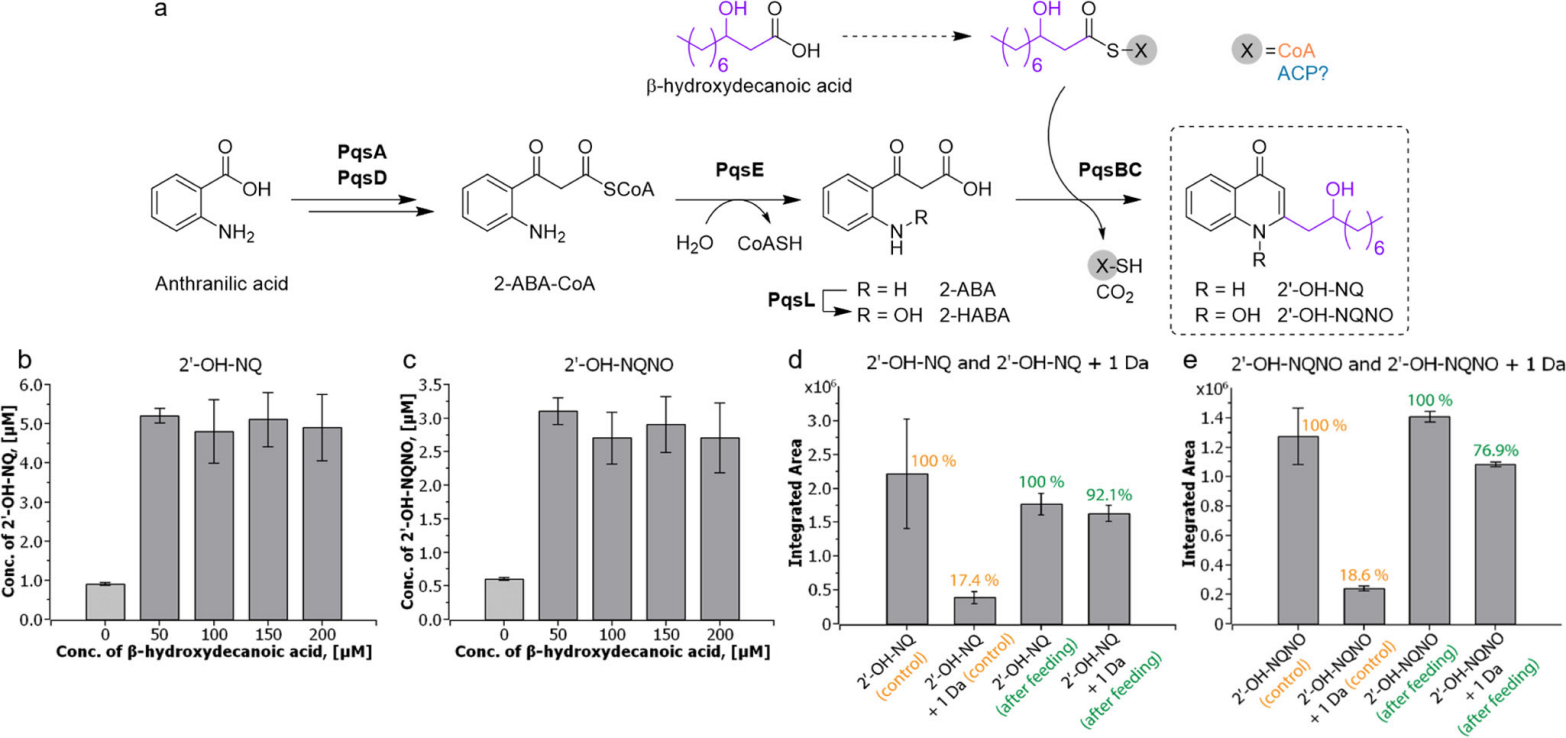
Quinolone production in dependence of fatty acid availability. **a** β-Hydroxydecanoic acid incorporation in 4(1*H*)-quinolone biosynthesis in *P. aeruginosa*. Production levels of 2′-OH-NQ (**b**) and 2′-OH-NQNO (**c**) in *P. aeruginosa* PAO1 after feeding β-hydroxydecanoic acid. Production levels of **d** 2′-OH-NQ and 2′-OH-NQ + 1 Da; **e** 2′-OH-NQNO and 2′-OH-NQNO + 1 Da measured in *P. aeruginosa* PAO1 in control sample (orange labels) and after feeding 100 µM deuterated β-hydroxydecanoic acid (**3d**) to bacterial cultures (green labels). Predicted m/z distribution for 2′-OH-NQ [M + H]^+^: 288.1959 (100.0%), 289.1992 (19.5%), 290.2026 (1.8%). Predicted m/z distribution for 2′-OH-NQNO [M + H]^+^: 304.1908 (100.0%), 305.1941 (19.5%), 306.1975 (1.8%). Mean values are given for biological triplicates with the error bars representing the population standard deviation.

To test if exogenous addition of hydroxylated fatty acid can boost the production of hydroxylated quinolones, we performed a feeding experiment with synthetic β-hydroxydecanoic acid, the proposed biosynthetic precursor of 2′-OH-NQ and 2′-OH-NQNO. Native levels of 2′-OH-NQ and 2′-OH-NQNO production in *P. aeruginosa* PAO1 were quantified by LC–MS/MS and compared to those after the fatty acid feeding. Changes in NQNO levels were analyzed as well for comparison (Fig. 4).

Remarkably, the external addition of β-hydroxydecanoic acid to *P. aeruginosa* PAO1 cultures led to a significant boost in 2′-OH-NQ and 2′-OH-NQNO production (Fig. 4b, c, S5a-S6a, and Table S15). 2′-OH-NQ concentrations increased 5.8-fold from native 0.26 mg L^−1^ (0.9 µM) up to 1.51 mg L^−1^ (5.3 µM) after feeding 50 µM of the fatty acid, and for 2′-OH-NQNO 5.5-fold from 0.17 mg L^-1^ (0.6 µM) up to 0.93 mg L^−1^ (3.2 µM). These results demonstrate that the production of 2′-OH-NQ and 2′-OH-NQNO can be strongly increased by exogenic supplementation of β-hydroxydecanoic acid. Feeding higher concentrations of β-hydroxydecanoic acid, however, did not further increase production levels of 2′-OH-NQ and 2′-OH-NQNO. In contrast, NQNO production considerably decreased by β-hydroxydecanoic acid supplementation (Fig. S7a). This may be explained by substrate competition of added β-hydroxydecanoic acid with native decanoic acid for utilization in quinolone biosynthesis by the enzyme complex PqsBC. Strikingly, feeding of synthetic β-oxodecanoic acid to *P. aeruginosa* PAO1 did not lead to a detectable production of 2′-oxo-NQ and 2′-oxo-NQNO, which suggests that direct incorporation of β-oxo fatty acids in 4-quinolone biosynthesis is not possible. However, as β-oxodecanoic acid is a precursor of β-hydroxydecanoic acid in the fatty acid biosynthesis, we still observed a β-oxodecanoic acid dependent increase in the concentrations of 2′-OH-NQ and 2′-OH-NQNO (Figs. S5b and S6b). Surprisingly, NQNO concentration was nearly two times decreased after β-oxodecanoic acid addition and remained unaffected with the increase of fatty acid concentration (Fig. S7b). To investigate the possibility of a direct incorporation of β-hydroxydecanoic acid, we performed an additional feeding experiment in *P. aeruginosa* PAO1 with 100 µM of the deuterated analog β-hydroxydecanoic acid-d (**3d**). LC–MS/MS analysis of the [M + 1 Da + H]^+^ signal for 2′-OH-NQ and 2′-OH-NQNO revealed a 4.2-fold and 4.6-fold increase in production of the parent mass + 1 Da, respectively, compared to the DMSO control (Figs. 4d, e, S12, and Table S14) suggesting that hydroxylated fatty acids can be utilized as direct substrates of the quinolone biosynthesis machinery.

### Investigation of biological activities

**Fig. 5:**
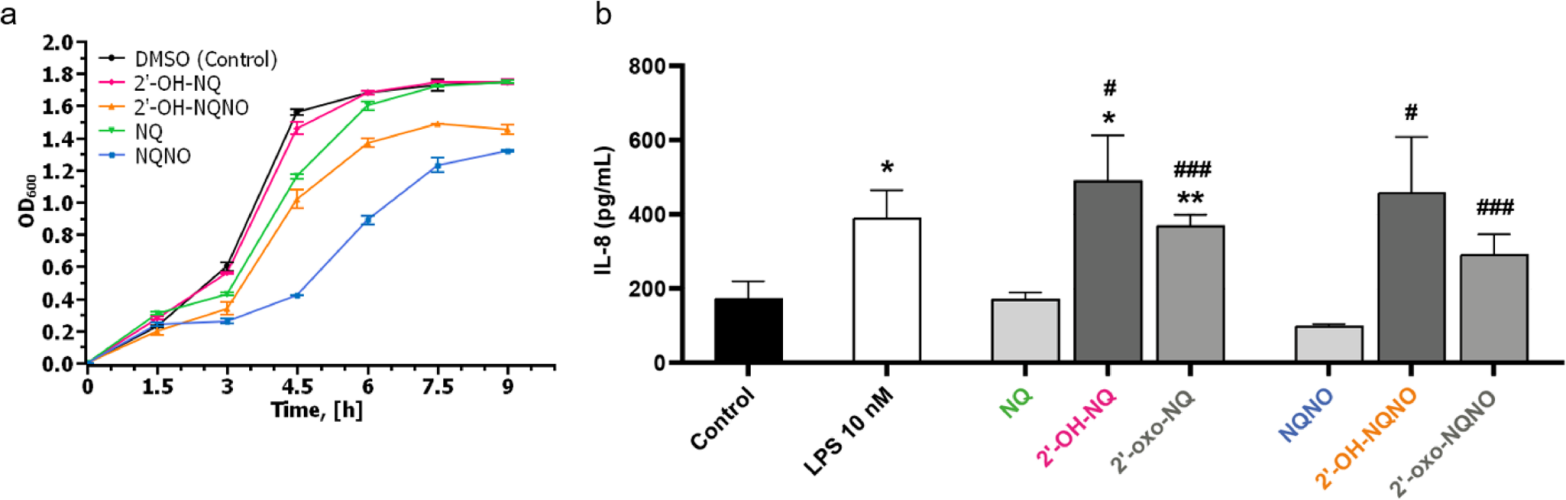
Influence of quinolones on *S. aureus* growth and host immune response. **a** Growth curves of *S. aureus* USA300 in the presence of 50 µM 2′-OH-NQ (pink), 2′-OH-NQNO (orange), NQ (green), NQNO (blue), and DMSO as a control (black). Mean values are given for biological triplicates with the error bars representing the population standard deviation. **b** IL-8 release from differentiated Caco-2 cells after 24 h of treatment with medium (Control), 10 nM LPS, 100 nM NQ, 100 nM 2′-OH-NQ, 100 nM 2′-oxo-NQ, 100 nM NQNO, 100 nM 2′-OH-NQNO, and 100 nM 2′-oxo-NQNO. Data are presented as Mean ± SEM of five independent biological repetitions in triplicates. * (*p* < 0.05) or ** (*p* < 0.01) indicates statistical significance compared with the control group. # (*p* < 0.05) or ### (*p* < 0.001) indicates statistical significance compared with NQ or NQNO.

Some quinolone *N*-oxides exhibit strong antimicrobial effects^6,14^ and we were thus interested to examine the antibiotic activity of the new quinolones **1g,** **1h,** **2g**, and **2h** against the methicillin-resistant *S. aureus* (MRSA) strain USA300. Since an endpoint assay testing metabolic activity did not indicate inhibition after 8 h at up to 200 µM (Fig. S3), we performed growth curve measurements for a more detailed assessment (Supplementary Data 2). Hereby, NQ derivatives were always less active than the corresponding NQNOs (Figs. 5a and S4). Interestingly, 2′-OH-NQ and 2′-OH-NQNO were both considerably less active than their unsubstituted counterparts, which suggests that the overall antimicrobial effect of this new class of quinolones against *S. aureus* is even lower compared to 2-alkyl-4(1*H*)-quinolones and their *N*-oxides. Quinolones produced by *P*. *aeruginosa* exhibit great functional diversity ranging from quorum sensing signals, antibiotics, and iron traps to modulating the innate immune response of their hosts^16^. We thus compared the effects of our new quinolones on cytokine response with that of NQ and NQNO. While none of the compounds (1–500 nM) exhibited cytotoxicity (Figs. S8 and S10), we observed a very potent effect on the production of pro-inflammatory interleukine-8 (IL-8) by Caco-2 cells already at 100 nM with all 2′-hydroxy and 2′-oxo substituted NQs and NQNOs (Fig. 5b, S9, S11, and Supplementary Data 3). At 100 nM and 500 nM, 2′-OH-NQ showed hereby the strongest effect, comparable with the stimulation by 10 nM LPS. In contrast, neither NQ nor NQNO showed any stimulation of IL-8 even at 500 nM (Fig. 5b and S11). Chronic lung inflammation mediated by the neutrophil recruiting chemokine IL-8 plays an important role in cystic fibrosis and is stimulated by infections with *P*. *aeruginosa*^17,18^. Stimulation of proinflammatory IL-8 by *P*. *aeruginosa* is also observed during infection of intestinal epithelial cells^19^. Surprisingly, the effect of AQs and AQNOs on IL-8 production has not been investigated before. However, it has been shown that the quorum sensing signal 3-oxo-dodecanoyl-ʟ-homoserine lactone of *P*. *aeruginosa* can induce IL-8 production, although its activity was reported in the upper micromolar range, i.e. at 300- to 1000-fold higher concentrations compared to 2′-OH-NQ^20,21^. Regarding 2-alkyl-4(1*H*)-quinolones, immune modulating effects have only been studied on the example of various other cytokines with the quorum sensing signals HHQ and the Pseudomonas quinolone signal (PQS). These studies show that HHQ and PQS at high concentrations exhibit activities suppressing the host innate immune response by down-regulating the NF-κB pathway^22^, inhibiting mitogen-stimulated IL-2 release^23^, or decreasing the LPS-stimulated production of IL-12^24^. These effects were observed in the micromolar concentration range and are hence significantly less potent than the nanomolar immune modulatory effects by 2′-OH-NQ and 2′-OH-NQNO described in the present work. Interestingly, 2′-OH-NQ also has been recently reported as a metabolite of a *Pseudomonas* strain isolated from a Chinese soil sample^25^. Additionally, this metabolite class was putatively identified using feature-based molecular networking analysis for secondary metabolites of *P. aeruginosa*, although the positioning of the hydroxylation in the side chain could not be resolved^26^.

Our results suggest that with native concentrations of up to 0.55 mg L^−1^ (1.9 µM), 2′-OH-NQ is one of the main 4(1*H*)-quinolones of *P*. *aeruginosa* strains. Already at 20-fold lower concentrations, we have shown potent stimulation of cytokine IL-8 by 2′-OH-NQ, indicating that host immune modulation could be achieved under physiologically relevant concentrations. While the biosynthesis of 2-alkyl-4(1*H*)-quinolones via the PqsBC complex is well established, it remained unclear if and how side-chain oxidized compounds could be produced. Our results with isotope labeled β-hydroxydecanoic acid suggest that oxidized fatty acids can be used even directly as substrates of this promiscuous biosynthetic pathway.

## Conclusions

We have reported the first synthesis of 2′-hydroxy and 2′-oxo substituted 2-alkyl-4(1*H*)-quinolones and demonstrated that 2′-OH-NQ and 2′-OH-NQNO but not the corresponding 2′-oxo-NQ and 2′-oxo-NQNO are naturally produced by *P*. *aeruginosa* strains PAO1 and PA14. We have shown that 2′-OH-NQ occurs in remarkably high concentrations and is together with NQ one of the major 4(1*H*)-quinolones of *P*. *aeruginosa*. Importantly, our results indicate that exogenous fatty acids may greatly boost product concentration and that 2′-OH-NQ has strong immunomodulatory activity with nanomolar potency.

## Methods

### Materials

See Supplementary Information (page 2).

### Preparation of overnight cultures

A small amount of a bacterial cryo-stock (20% glycerol, stored at −80 °C) was inoculated in 3 mL LB-(lysogenic broth)-Lennox medium (LB medium) in sterile 13 mL polypropylene tubes (Sarstedt, ref 62.515.028), and allowed to grow for 16–18 h at indicated growth conditions (Table S1).

### Growth curves

An overnight culture was diluted 1:1000 in 3 mL of LB medium in a sterile 13 mL polypropylene tube. Quinolones were added from DMSO stocks, to reach their final concentration of 50 µM. The cultures were incubated at 37 °C and 180 rpm. The growth was monitored by every 1.5 h measurement of the OD_600_ value (Fisher Scientific cell density meter model 40) of 100 μL culture in plastic cuvettes (Sarstedt UV-transparent cuvettes for use >220 nm, 12.5 × 12.5 × 45 mm). The experiment was performed in triplicates.

### Feeding experiments

An overnight culture (45 µL) of *P. aeruginosa* PAO1 was inoculated into 3 mL of LB medium in sterile 15 mL polypropylene centrifugal tubes with screw caps (VWR). A respective DMSO stock solution of fatty acid (or DMSO as a control) (3 µL) was added to reach its final concentration of 50, 100, 150, and 200 µM. Caps of the tubes were loosely opened by a 180 degree turn and fixed in this position to ensure equal oxygen supply. Cultures were incubated for 9 h at 37 °C in a shaking incubator at 180 rpm. After incubation, samples were centrifuged at 4500 rpm for 5 min and supernatants were sterile filtrated. Three hundred microliters of culture supernatant were added in 1.5 mL glass vials (LABSOLUTE, Art. Nr. 7612960) with caps containing a PTFE membrane (LABSOLUTE, Art. Nr. 7623097). Three hundred microliters of EtOAc was added and immediately vortexed for 5 s. After the separation of organic and water phases, 100 μL of the EtOAc layer was transferred via pipetting into mass spec vials containing a glass insert (MACHEREY-NAGEL, Art. Nr. 702007). EtOAc was evaporated by a gentle stream of nitrogen. For LC–MS/MS analysis, 100 μL of sample solvent (MeOH/H_2_O 1:1) was added into glass inserts and the residue redissolved. The experiment was performed in triplicates.

### Cell culture and treatment

Caco-2 cells were maintained at 37 °C and 5% CO_2_ in Dulbecco’s modified Eagle medium (DMEM) supplemented with 10% fetal bovine serum, 2% l-glutamine and 1% penicillin/streptomycin under a humidified atmosphere. For differentiation into an enterocyte cell model, cells were seeded in 96-well plates with the density of 3 × 10^4^ cells/well, and the medium was replaced with fresh culture medium every 2–3 days until differentiation was complete (after 21 days). For treatment, the differentiated Caco-2 cells were incubated with fresh medium alone (control) and fresh medium containing 10 nM LPS (lipopolysaccharide from *Escherichia coli*), 1, 10, 50, 100, 200 and 500 nM quinolones. After 24 h of incubation, the supernatants were collected for cytokine analysis, and cells were used for cell viability estimation based on MTT assay.

### Cell viability

Caco-2 cells in each well were incubated with 100 µL MTT working solution (0.83 mg mL^−1^) diluted in serum-free medium for 10 min. By replacing the MTT working solution with 150 µL DMSO, the formazan product, formed during the incubation, was dissolved. The absorbance was measured at 570 nm with 650 nm as a reference wavelength.

### Measurement of Interleukin-8 (IL-8) level

The concentration of IL-8 was evaluated by ELISA kits (BD Biosciences) based on the manufacturer’s instructions. Data are expressed in pg mL^-1^.

### Quantification of quinolones in bacterial cultures

An overnight culture (60 µL) was inoculated into 4 mL of LB medium in a sterile 15 mL polypropylene centrifugal tubes with screw caps (VWR). Caps of the tubes were loosely opened by a 180° turn and fixed in this position to ensure equal oxygen supply. Cultures were incubated for 3, 6, 9, 12, and 24 h at 37 °C in a shaking incubator at 180 rpm. After incubation, samples were centrifuged at 4500 rpm for 5 min and supernatants were sterile filtrated. Three hundred microliters of culture supernatant were added in 1.5 mL glass vials (LABSOLUTE, Art. Nr. 7612960) with caps containing a PTFE membrane (LABSOLUTE, Art. Nr. 7623097). Three hundred microliters of EtOAc was added and immediately vortexed for 5 s. After the separation of organic and water phases, 100 μL of the EtOAc layer was transferred via pipetting into mass spec vials containing a glass insert (MACHEREY-NAGEL, Art. Nr. 702007). The EtOAc was evaporated by a gentle stream of nitrogen. For LC–MS/MS analysis, 100 μL of sample solvent (MeOH/H_2_O 1:1) was added into glass inserts and the residue redissolved. The experiment was performed in triplicates.

### LC–MS/MS analysis

Ultra-high performance liquid chromatography was performed on a Dionex Ultimate 3000 UHPLC (Thermo Fisher Scientific) and Vanquish™ UHPLC system (Thermo Fisher Scientific) using a Nucleodur C18 Gravity-SB 100 ×2 mm, 3 μm column (Macherey-Nagel). The flow rate was 0.5 mL min^−1^ and the column temperature was kept at 40 °C. The injection volume was 10 μL. Eluent A was 0.1% formic acid in water and eluent B was 0.1% formic acid in acetonitrile. The gradient was set to 20–100% B in 10 min, 100% B for 2 min, 100–20% B in 1 min, and 20% B for 2 min. MS/MS analysis was performed by Finnigan™ TSQ Quantum (Thermo Scientific) and TSQ Series II Quantum (Thermo Fisher Scientific) mass spectrometers. A heated-electrospray ionization (HESI-II probe, Thermo Scientific) was used as an ion source. In the optimized conditions the ion spray voltage was 3500 V, vaporizer temperature 300 °C, capillary temperature 380 °C, sheath gas pressure 60 psi, ion sweep gas pressure 2 psi, and aux gas 10 psi. The fragmentation pattern of quinolone standards was acquired in a Product Ion Scan mode using a fixed collision energy of 30 eV to fragment the corresponding precursor ion before recording the fragments in a mass range of m/z 130–350. Quinolones we quantified in Selected Reaction Monitoring scan mode. MS/MS spectra were acquired in a positive mode. The software Quan Browser Thermo Xcalibur was used for quantitative analysis. The peak area of the respective product ion was fitted by linear regression versus the known concentrations to generate a standard curve. Note: Analysis of extracts after feeding of deuterated β-hydroxydecanoic acid (3d) were performed on Vanquish™ UHPLC system (Thermo Fisher Scientific) in combination with TSQ Series II Quantum (Thermo Fisher Scientific), while all other LC–MS/MS experiments on Dionex Ultimate 3000 UHPLC (Thermo Fisher Scientific) in combination with Finnigan™ TSQ Quantum (Thermo Fisher Scientific).

### MS^2^ assignments

See Supplementary Information (page 6).

### Synthesis

See Supplementary Information (page 15).

### Reporting summary

Further information on research design is available in the Nature Portfolio Reporting Summary linked to this article.

## Supplementary information

Supplemental Information

Description of Additional Supplementary Files

Supplementary Data 1

Supplementary Data 2

Supplementary Data 3

Reporting Summary

## Data Availability

LC–MS/MS spectra are deposited in the GNPS database under the spectra identifiers given in the Supplementary Information. NMR data are provided in Supplementary Data 1 file. Optical density values from growth curve experiment are provided in Supplementary Data 2. IL-8 release data are provided in Supplementary Data 3. All relevant data are also available from the authors.
