# Supplementary material for: Biosynthetic flexibility of Pseudomonas aeruginosa leads to hydroxylated 2-alkylquinolones with proinflammatory host response
Source: Commun Chem. 2023 Jul 3;6:138. doi: 10.1038/s42004-023-00937-y (PMC10318067; doi:10.1038/s42004-023-00937-y)
Supplement: Supplementary file 1 — Supplemental Information [file 42004_2023_937_MOESM1_ESM.pdf]

Supporting Information

Biosynthetic flexibility of *Pseudomonas aeruginosa* leads to hydroxylated 2-Alkylquinolones with proinflammatory host response

Viktoriia Savchenko[a,b], Dávid Szamosvári[a], Yifan Bao[b,c], Marc Pignitter[c] and Thomas Böttcher*[a]

[a] Faculty of Chemistry, Institute for Biological Chemistry & Centre for Microbiology and Environmental Systems Science, Department of Microbiology and Ecosystems Science, University of Vienna, Josef-Holaubek-Platz 2 (UZA II), 1090 Vienna, Austria

[b] Vienna Doctoral School in Chemistry (DoSChem), University of Vienna, Währinger Str. 42, 1090 Vienna, Austria

[c] Faculty of Chemistry, Institute of Physiological Chemistry, University of Vienna, Josef-Holaubek-Platz 2, 1090 Vienna, Austria

E-mail: thomas.boettcher@univie.ac.at

Table of Contents

1. Supplementary Methods 2
    a. Materials and Methods 2
    b. Biological experiments 2
    c. Analytical methods 4
2. MS^2 Assignments 6
3. Synthesis 15
4. Supplementary Tables 28
5. Supplementary Figures 36
6. Supplementary References 45

1. Supplementary Methods

a. Materials and Methods

Chemicals and solvents for the synthesis were purchased from Sigma-Aldrich, Carl Roth, Acros Organics or VWR Chemicals and were used without further purification. Distilled technical grade solvents and silica gel 60 A (Carl Roth) were used for silica gel chromatography. Thin layer chromatography (TLC) was performed using aluminium sheets "TLC Silica gel 60 F254" from Merck Millipore® and analysed with short/long wave UV-light or by permanganate staining. NMR spectra were obtained on Bruker Avance-III 400 and Bruker Avance-III 600 NMR spectrometers at ambient temperature. Multiplicities are given as follows: s - singlet, d - doublet, t - triplet, q - quartet, quint. - quintet, m - multiplet. Chemical shifts (δ) are given in parts per million (ppm) relative to the solvent residual signal with $CDCl_3$ δ_H = 7.26 ppm and δ_C = 77.16 ppm, DMSO-d_6 δ_H = 2.50 ppm and δ_C = 39.52 ppm, CD_3OD δ_H = 3.31 ppm and δ_C = 49.00 ppm.[1]. The obtained data were processed and analysed with MestReNova 14.2.0-26256 software. High-resolution mass spectrometry data were obtained on a Hybrid FT Mass Spectrometer LTQ Orbitrap Velos (Thermo Scientific). LC-MS/MS analysis was performed on Dionex Ultimate 3000 UHPLC (Thermo Fisher Scientific) in combination with Finnigan™ TSQ® Quantum (Thermo Fisher Scientific) and on Vanquish™ Horizon/Flex UHPLC system (Thermo Fisher Scientific) in combination with TSQ® Series II Quantum (Thermo Fisher Scientific).

b. Biological experiments

Preparation of overnight cultures
A small amount of a bacterial cryo-stock (20% glycerol, stored at -80 °C) was inoculated in 3 mL LB-(lysogenic broth)-Lennox medium (LB medium) in sterile 13 mL polypropylene tubes (Sarstedt, ref 62.515.028), and allowed to grow for 16-18 h at indicated growth conditions (**Table S1**).

Growth curves
An overnight culture was diluted 1:1000 in 3 mL of LB medium in a sterile 13 mL polypropylene tube. Quinolones were added from DMSO stocks, to reach their final concentration of 50 µM. The cultures were incubated at 37°C and 180 rpm. The growth was monitored by every 1.5 h measurement of the OD_{600} value (Fisher Scientific cell density meter model 40) of 100 µL culture in plastic cuvettes (Sarstedt UV-transparent cuvettes for use >220 nm, 12.5 x 12.5 x 45mm). The experiment was performed in triplicates.

Antibiotic activity assay
An overnight culture of *S. aureus* USA300 was diluted with LB medium (1:1000) in a sterile 13 mL polypropylene tube. 99 µl of the bacteria suspension and 1 µL of a respective DMSO stock solution of quinolone (or DMSO for medium control) were added subsequently in a round bottom 96-well plate. The plate was incubated at 37°C and 180 rpm for 8 h. After incubation, 100 µL of a 0.02% resazurin solution* were added and the plate was incubated for 2 h at 37°C and 180 rpm. An inhibition was considered positive if the solution remained blue. Wells that

showed a pink or violet colour or were colourless were considered negative for inhibition. The experiment was performed in duplicates.

*20 mg of resazurin were added to 100 mL of LB media. After sonication, the solution was sterile filtered and stored for up to 2 weeks at 4°C.

Feeding experiments

An overnight culture (45 µL) of *P. aeruginosa* PAO1 was inoculated into 3 mL of LB medium in sterile 15 mL polypropylene centrifugal tubes with screw caps (VWR). A respective DMSO stock solution of fatty acid (or DMSO as a control) (3 µL) was added to reach its final concentration of 50, 100, 150, and 200 µM. Caps of the tubes were loosely opened by a 180 degree turn and fixed in this position to ensure equal oxygen supply. Cultures were incubated for 9 h at 37˚C in a shaking incubator at 180 rpm. After incubation, samples were centrifuged at 4500 rpm for 5 min and supernatants were sterile filtrated. 300 µL of culture supernatant were added in 1.5 mL glass vials (LABSOLUTE, Art. Nr. 7612960) with caps containing a PTFE membrane (LABSOLUTE, Art. Nr. 7623097). 300 µL of EtOAc was added and immediately vortexed for 5 sec. After the separation of organic and water phases, 100 µL of the EtOAc layer was transferred via pipetting into mass spec vials containing a glass insert (MACHEREY-NAGEL, Art. Nr. 702007). EtOAc was evaporated by a gentle stream of nitrogen. For LC-MS/MS analysis, 100 µL of sample solvent (MeOH/H_2O 1:1) was added into glass inserts and the residue redissolved. The experiment was performed in triplicates.

Cell culture and treatment

Caco-2 cells (obtained from ATCC) were maintained at 37°C and 5% CO_2 in Dulbecco's modified Eagle medium (DMEM) supplemented with 10% fetal bovine serum, 2% L-glutamine and 1% penicillin/streptomycin under a humidified atmosphere. For differentiation into an enterocyte cell model, cells were seeded in 96-well plates with the density of 3×10^4 cells/well, and the medium was replaced with fresh culture medium every 2 to 3 days until differentiation was complete (after 21 days).

For treatment, the differentiated Caco-2 cells were incubated with fresh medium alone (control) and fresh medium containing 10 nM LPS (lipopolysaccharide from *Escherichia coli*), 1, 10, 50, 100, 200 and 500 nM quinolones. After 24 h of incubation, the supernatants were collected for cytokine analysis, and cells were used for cell viability estimation based on MTT assay.

Cell viability

Caco-2 cells in each well were incubated with 100 µL MTT working solution (0.83 mg/mL) diluted in serum-free medium for 10 min. By replacing the MTT working solution with 150 µL DMSO, the formazan product, formed during the incubation, was dissolved. The absorbance was measured at 570 nm with 650 nm as a reference wavelength.

Measurement of Interleukin-8 (IL-8) level

The concentration of IL-8 was evaluated by ELISA kits (BD Biosciences) based on the manufacturer's instructions. Data are expressed in pg/mL.

c. Analytical methods

Quantification of quinolones in bacterial cultures

An overnight culture (60 µL) was inoculated into 4 mL of LB medium in a sterile 15 mL polypropylene centrifugal tubes with screw caps (VWR). Caps of the tubes were loosely opened by a 180 degree turn and fixed in this position to ensure equal oxygen supply. Cultures were incubated for 3, 6, 9, 12, and 24 h at 37˚C in a shaking incubator at 180 rpm. After incubation, samples were centrifuged at 4500 rpm for 5 min and supernatants were sterile filtrated. 300 µL of culture supernatant were added in 1.5 mL glass vials (LABSOLUTE, Art. Nr. 7612960) with caps containing a PTFE membrane (LABSOLUTE, Art. Nr. 7623097). 300 µL of EtOAc was added and immediately vortexed for 5 sec. After the separation of organic and water phases, 100 µL of the EtOAc layer was transferred via pipetting into mass spec vials containing a glass insert (MACHEREY-NAGEL, Art. Nr. 702007). The EtOAc was evaporated by a gentle stream of nitrogen. For LC-MS/MS analysis, 100 µL of sample solvent (MeOH/H_2O 1:1) was added into glass inserts and the residue redissolved. The experiment was performed in triplicates.

Calibration curves for quantification

MeOH stocks of all calibration standards were prepared at 1 mg/mL in glass vials and stored at -80°C for up to 3 months. Calibration standard samples for quantification were prepared in triplicates by serial dilution in sample solvent (MeOH/H_2O 1:1) (**Table S2**). Calibration equations and R-values were obtained using the Thermo Xcalibur Quan Browser.

LC-MS/MS analysis

Ultra-high performance liquid chromatography was performed on a Dionex Ultimate 3000 UHPLC (Thermo Fisher Scientific) and Vanquish™ UHPLC system (Thermo Fisher Scientific) using a Nucleodur C18 Gravity-SB 100 x 2 mm, 3 µm column (Macherey-Nagel). The flow rate was 0.5 mL min^{-1} and the column temperature was held at 40°C. The injection volume was 10 µL. Eluent A was 0.1% formic acid in water and eluent B was 0.1% formic acid in acetonitrile. The gradient was 20-100 % B in 10 min, 100 % B for 2 min, 100-20 % B in 1 min, and 20 % B for 2 min. MS/MS analysis was performed by Finnigan™ TSQ® Quantum (Thermo Scientific) and TSQ® Series II Quantum (Thermo Fisher Scientific) mass spectrometers. A heated-electrospray ionization (HESI-II probe, Thermo Scientific) was used as an ion source. In the optimized conditions the ion spray voltage was 3500 V, vaporizer temperature 300°C, capillary temperature 380°C, sheath gas pressure 60 psi, ion sweep gas pressure 2 psi, and aux gas 10 psi. The fragmentation pattern of quinolone standards was acquired in a Product Ion Scan mode using a fixed collision energy of 30 eV to fragment the corresponding precursor ion before recording the fragments in a mass range of m/z 130-350. Quinolones we quantified in Selected Reaction Monitoring scan mode. MS/MS spectra were acquired in a positive mode. The software Quan Browser Thermo Xcalibur was used for quantitative analysis. The peak area of the respective product ion was fitted by linear regression versus the known concentrations to generate a standard curve.

The LC-MS/MS spectra were made available in the GNPS database under the following spectrum identifiers:

CCMSLIB00011427575 (2'-OH-NQ synthetic standard)

CCMSLIB00011427576 (2'-OH-NQNO synthetic standard)

CCMSLIB00011427581 (2'-oxo-NQ synthetic standard)
CCMSLIB00011427582 (2'-oxo-NQNO synthetic standard)
CCMSLIB00011427574 (NQ synthetic standard)
CCMSLIB00011427583 (NQNO synthetic standard)

CCMSLIB00011427580 (2'-OH-NQ in P. aeruginosa PAO1 supernatant)
CCMSLIB00011427579 (2'-OH-NQ in P. aeruginosa PA14 supernatant)
CCMSLIB00011427578 (2'-OH-NQNO in P. aeruginosa PAO1 supernatant)
CCMSLIB00011427577 (2'-OH-NQNO in P. aeruginosa PA14 supernatant)

Note: extracts analysis after feeding of deuterated β-Hydroxydecanoic acid (**3d**) were performed on Vanquish™ UHPLC system (Thermo Fisher Scientific) in combination with TSQ® Series II Quantum (Thermo Fisher Scientific), while all other LC-MS/MS experiments on Dionex Ultimate 3000 UHPLC (Thermo Fisher Scientific) in combination with Finnigan™ TSQ® Quantum (Thermo Fisher Scientific).

2. MS^2 Assignments

Reference spectra (on Vanquish™ UHPLC + TSQ® Series II Quantum)

2’-OH-NQ (RT = 4.42; Parental mass = 288)

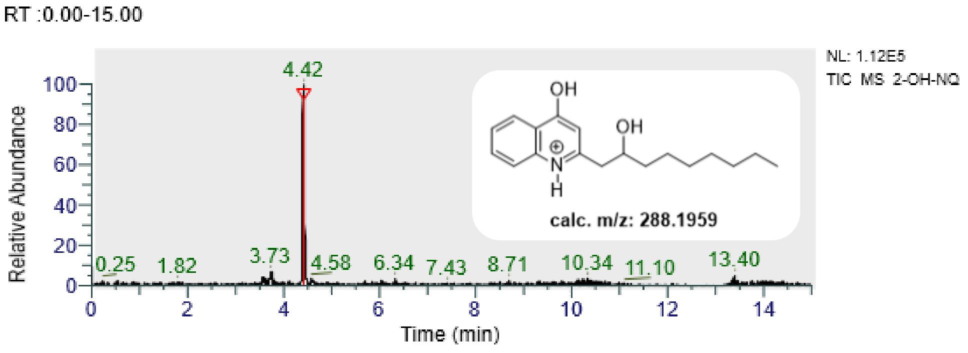

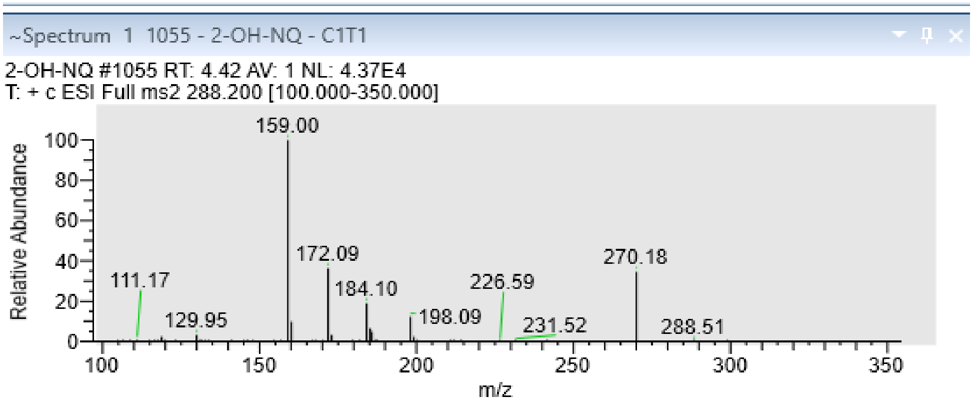

Extracts analysis (on Vanquish™ UHPLC + TSQ® Series II Quantum). Example spectra of detected 2’-OH-NQ and 2’-OH-NQNO in *P. aeruginosa* PAO1 extracts

2’-OH-NQ (RT = 4.46; Parental mass = 288)

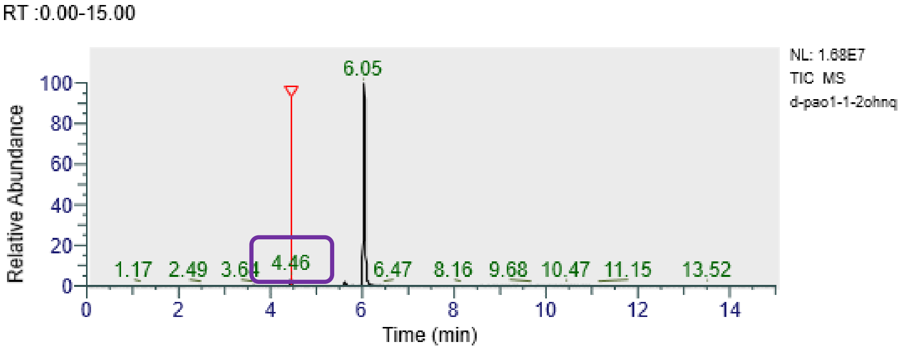

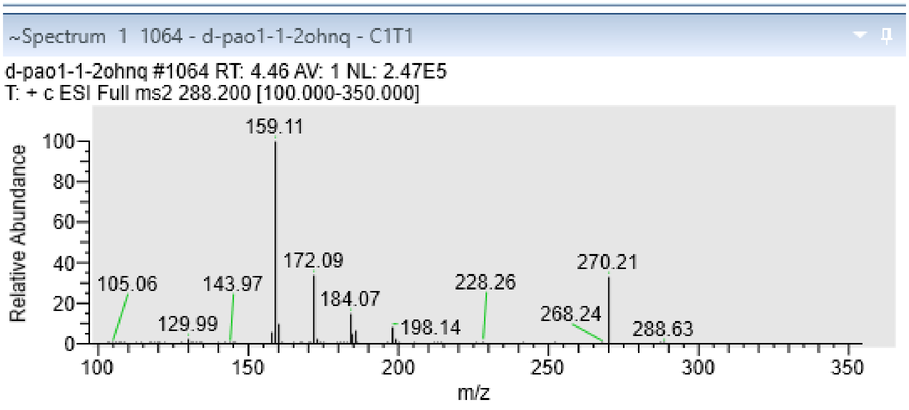

Extracts analysis (on Vanquish™ UHPLC + TSQ® Series II Quantum). Example spectra of detected 2’-OH-NQ + 1 Da in *P. aeruginosa* PAO1 extracts

2’-OH-NQ + 1 Da (RT = 4.46; Parental mass = 289)

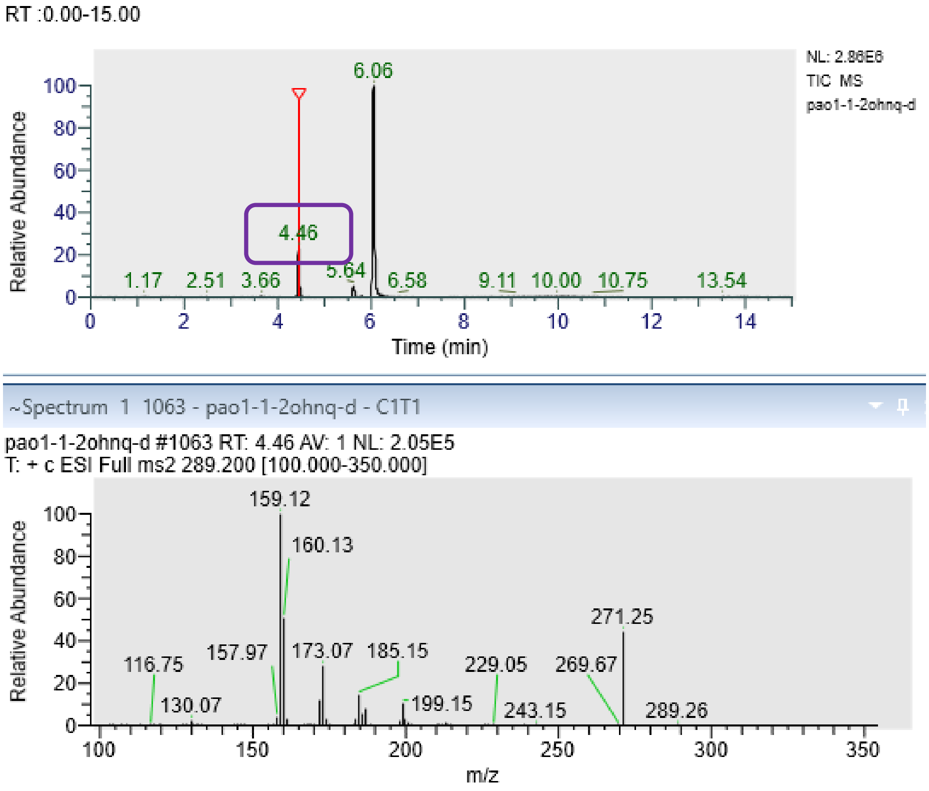

Reference spectra (on Vanquish™ UHPLC + TSQ® Series II Quantum)

2’-OH-NQNO (RT = 4.77; Parental mass = 304)

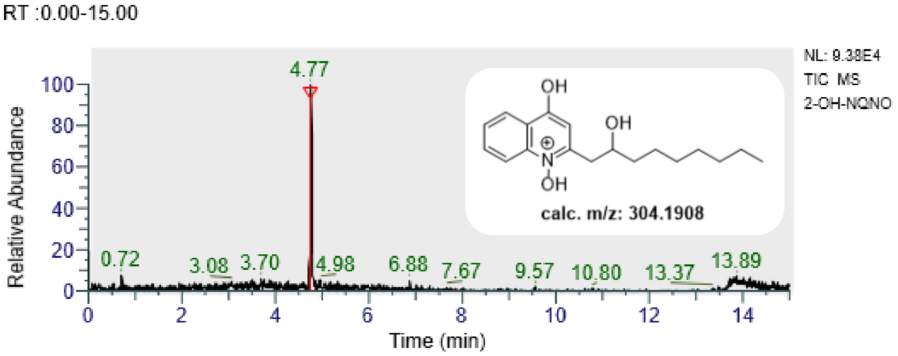

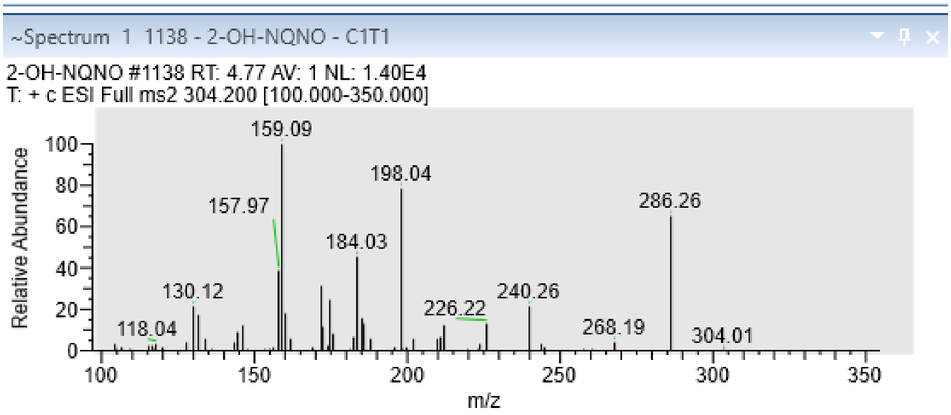

Extracts analysis (on Vanquish™ UHPLC + TSQ® Series II Quantum). Example spectra of detected 2’-OH-NQ and 2’-OH-NQNO in *P. aeruginosa* PAO1 extracts

2’-OH-NQNO (RT = 4.81; Parental mass = 304)

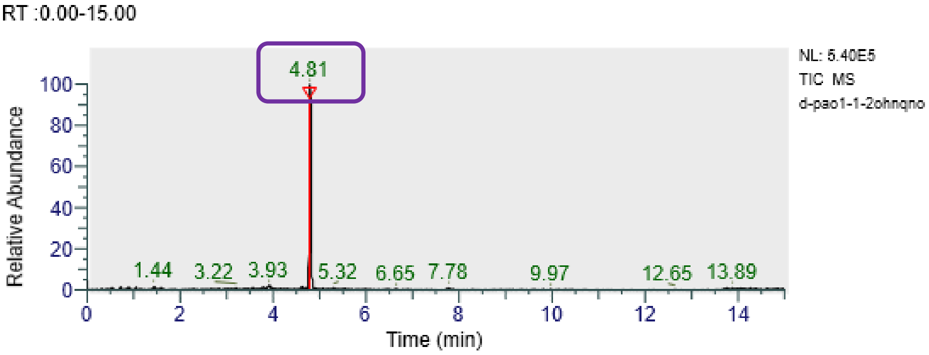

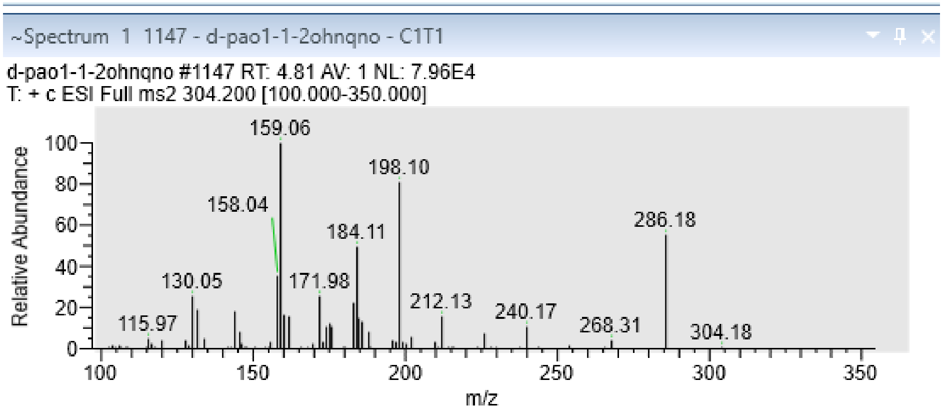

Extracts analysis (on Vanquish™ UHPLC + TSQ® Series II Quantum). Example spectra of detected 2'-OH-NQNO + 1 Da in *P. aeruginosa* PAO1 extracts

2'-OH-NQNO + 1 Da (RT = 4.81; Parental mass = 305)

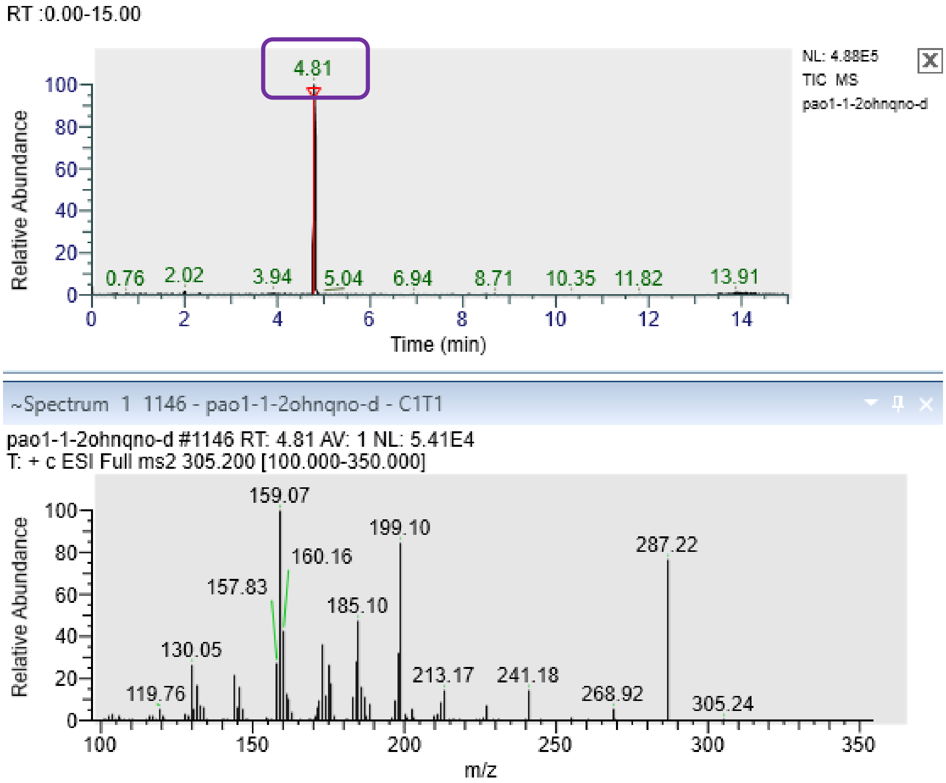

Reference spectra (on Dionex Ultimate 3000 UHPLC + Finnigan™ TSQ® Quantum)

2’-OH-NQ (RT = 5.72; Parental mass = 288)

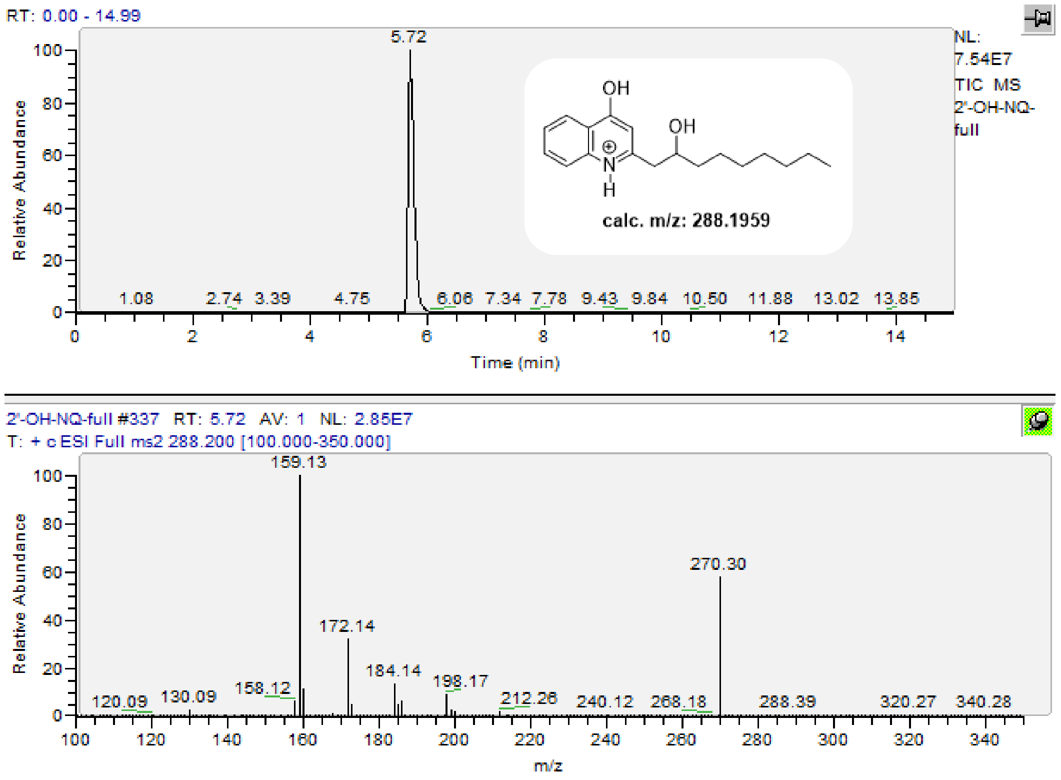

Extracts analysis (on Dionex Ultimate 3000 UHPLC + Finnigan™ TSQ® Quantum). Example spectra of detected 2’-OH-NQ and 2’-OH-NQNO in *P. aeruginosa* PA14 extracts

2’-OH-NQ (RT = 5.75; Parental mass = 288)

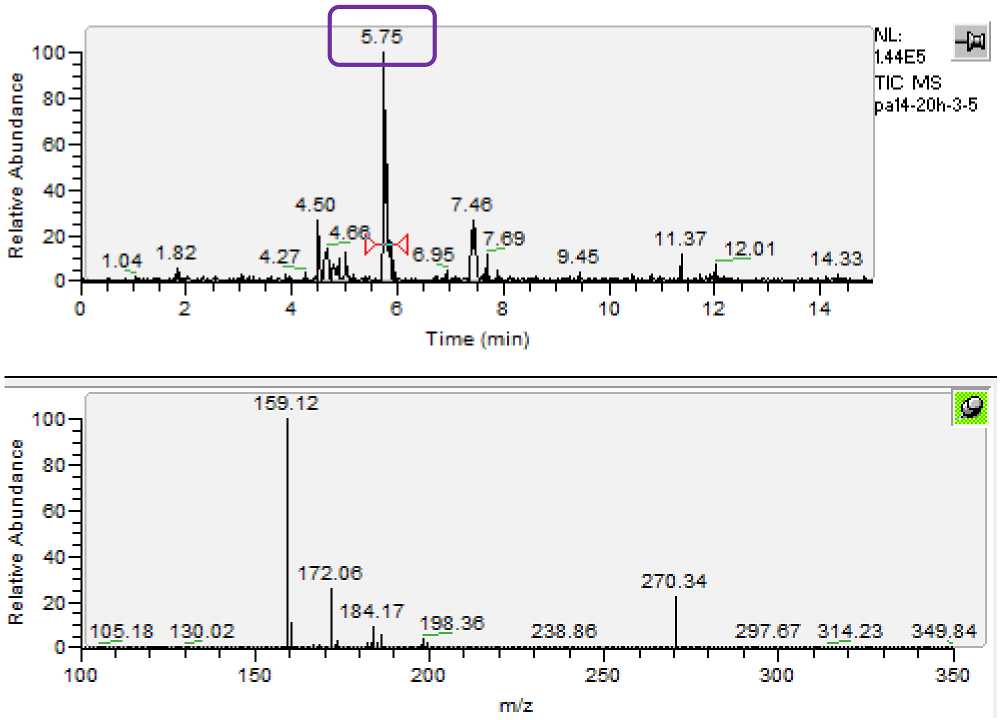

Reference spectra (on Dionex Ultimate 3000 UHPLC + Finnigan™ TSQ® Quantum)

2’-OH-NQNO (RT = 5.98; Parental mass = 304)

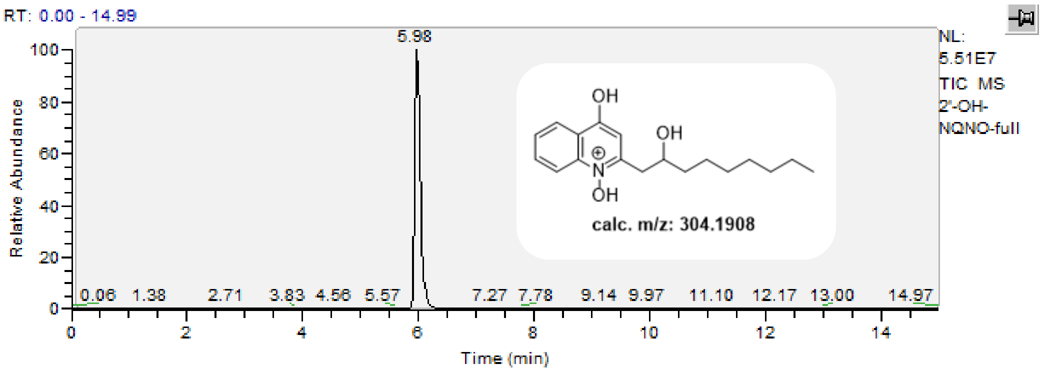

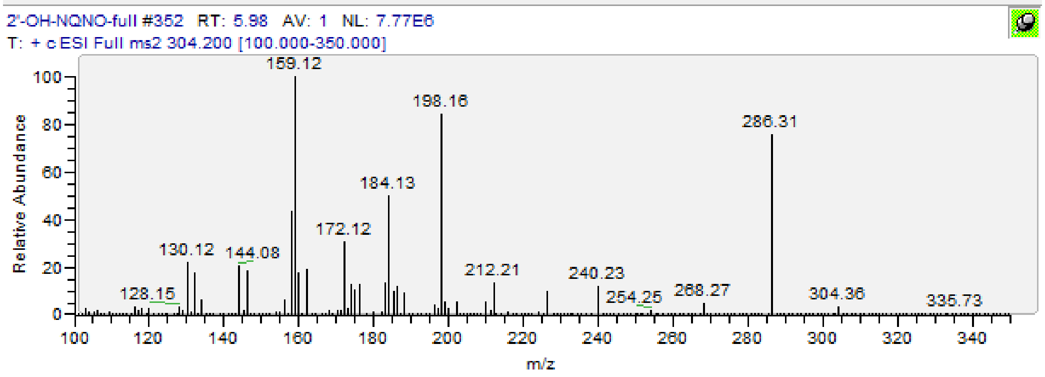

Extracts analysis (on Dionex Ultimate 3000 UHPLC + Finnigan™ TSQ® Quantum). Example spectra of detected 2’-OH-NQ and 2’-OH-NQNO in *P. aeruginosa* PAO1 extracts

2’-OH-NQNO (RT = 5.99; Parental mass = 304)

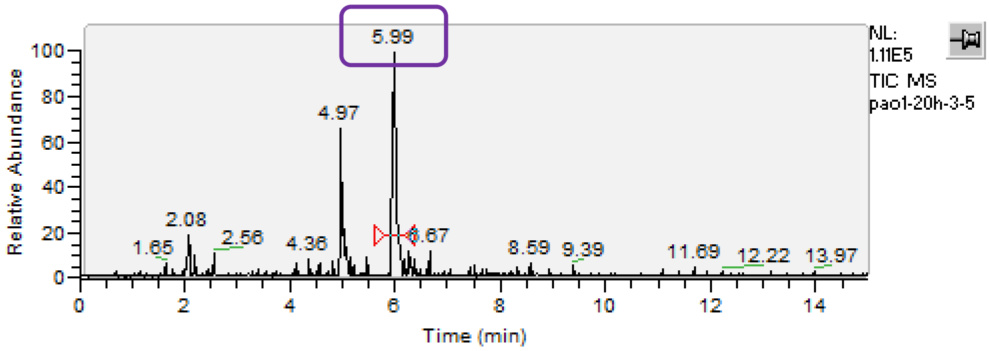

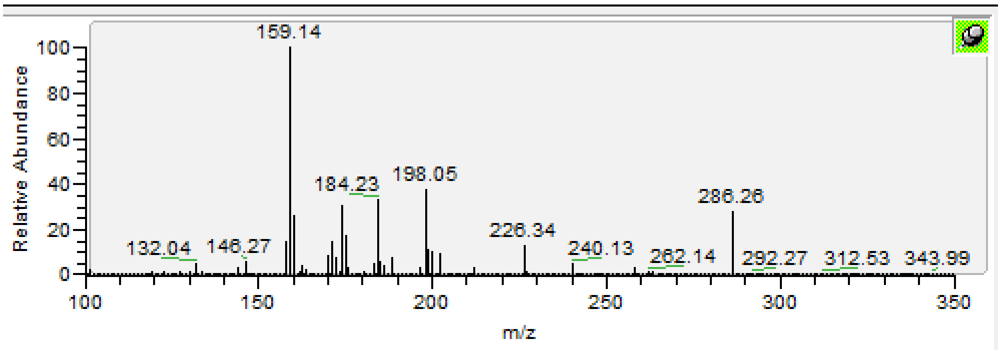

Reference spectra (on Dionex Ultimate 3000 UHPLC + Finnigan™ TSQ® Quantum)

2’-oxo-NQ (RT = 6.16; Parental mass = 286)

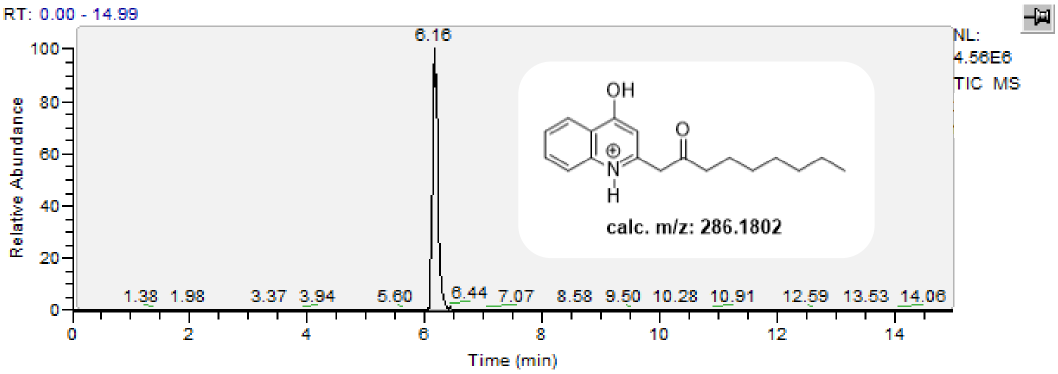

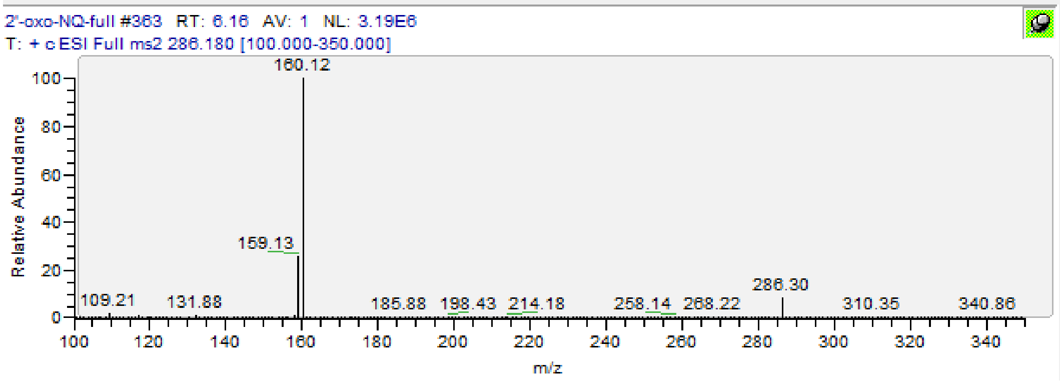

2’-oxo-NQNO (RT = 6.23; Parental mass = 302)

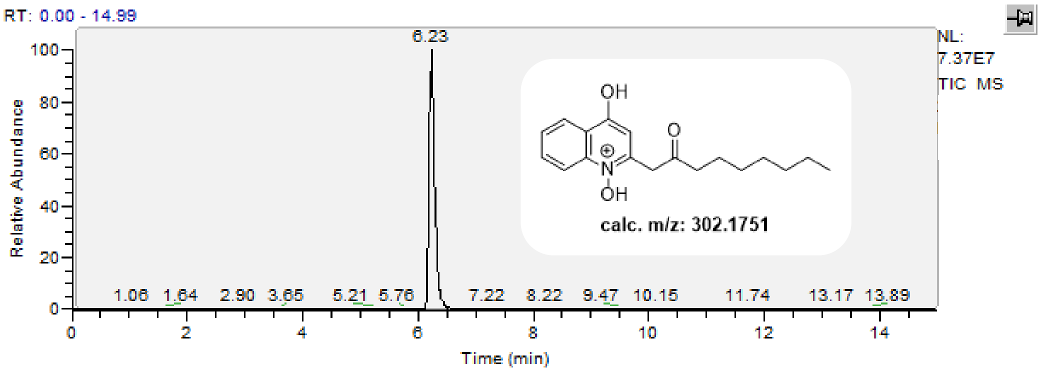

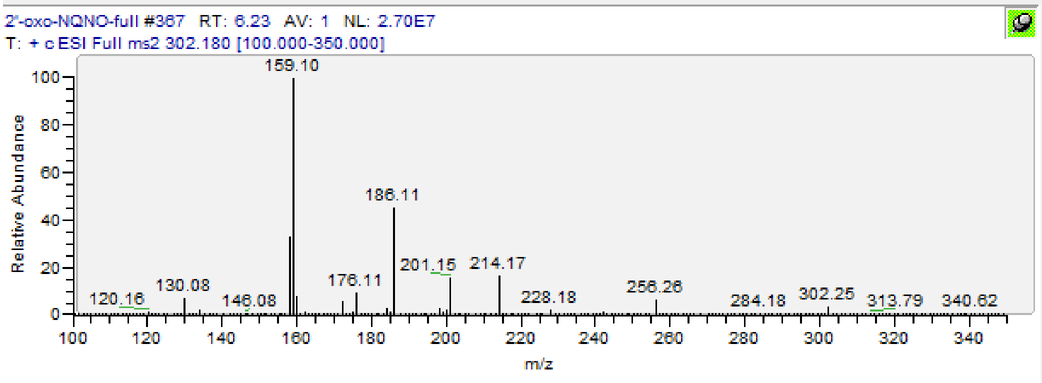

NQ (RT = 7.63; Parental mass = 272)

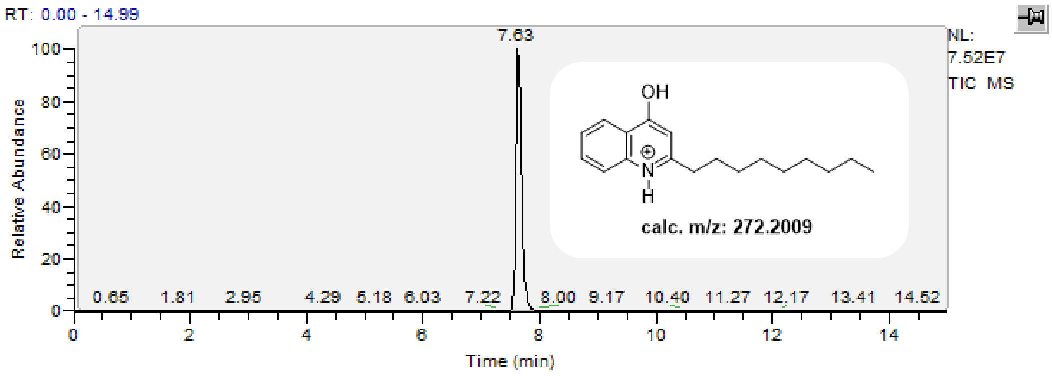

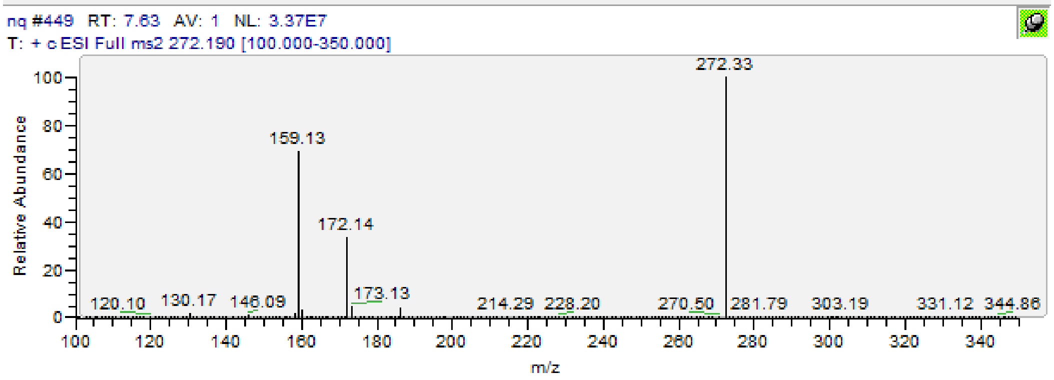

NQNO (RT = 7.39; Parental mass = 288)

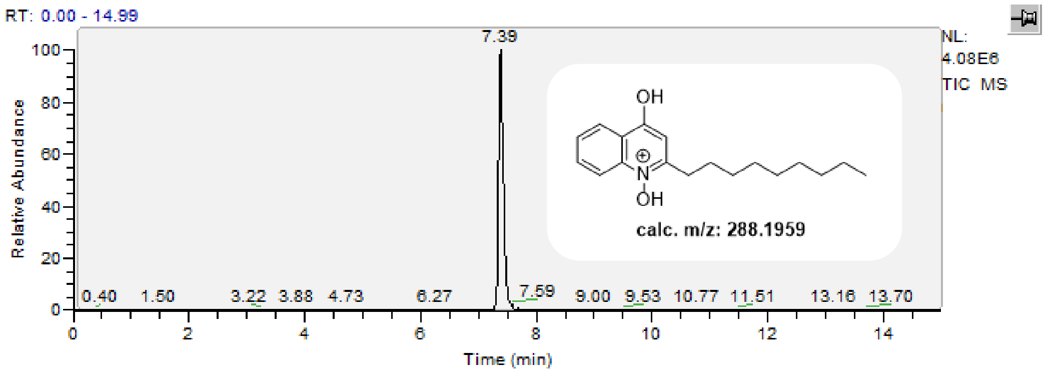

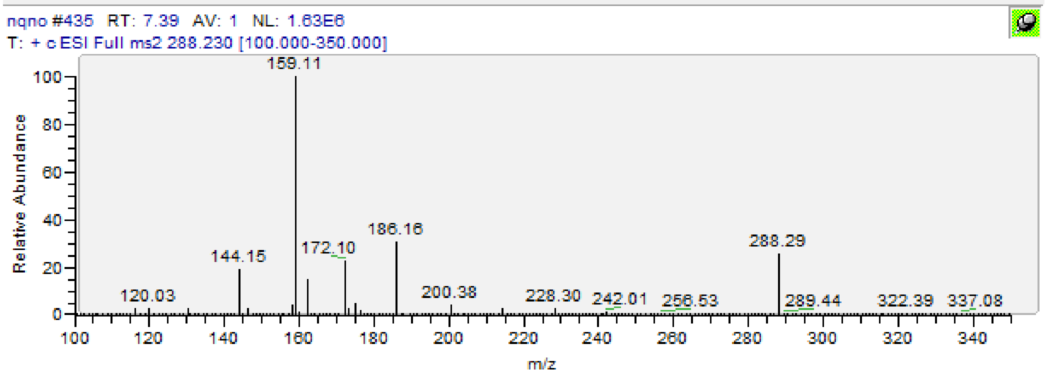

Proposed structures of fragment ions*

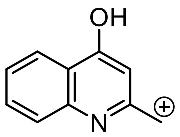

calc. m/z: 158.0601

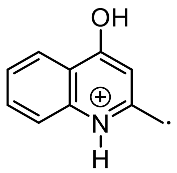

calc. m/z: 159.0679

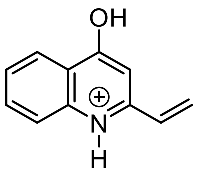

calc. m/z: 172.0757

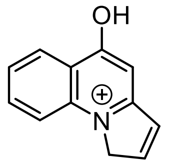

calc. m/z: 184.0757

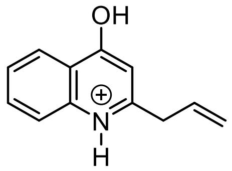

calc. m/z: 186.0914

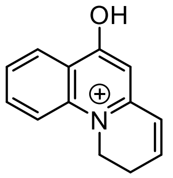

calc. m/z: 198.0914

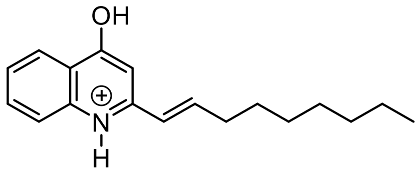

calc. m/z: 270.1853

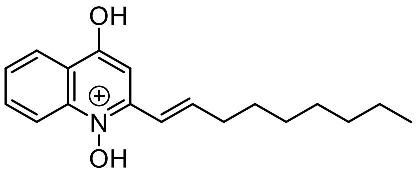

calc. m/z: 286.1802

*For relative abundance of detected fragments see **Table S3**.

3. Synthesis

Methyl 3-oxodecanoate (**a**)

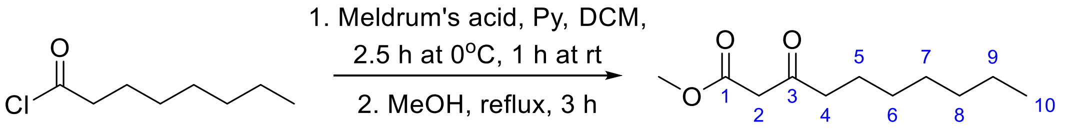

Compound **a** was synthesized based on procedures described by Zheng *et. al.* [2] and Vleeschouwer *et. al.* [3].

1. 2,2-dimethyl-1,3-dioxane-4,6-dione (Meldrum's acid) (17.72 g, 122.96 mmol) was dissolved in DCM (200 mL) and cooled to 0˚C. Pyridine (19.81 ml, 245.92 mmol) was added to this solution. After stirring the mixture for 40 min at 0˚C, octanoyl chloride (21.00 mL, 122.96 mmol) was added dropwise. The resulting orange solution was stirred at 0˚C for 2 h and at room temperature for 1 h. The obtained mixture was washed with 5% aq. HCl solution, distilled water, and Brine solution. The organic phase was dried over $MgSO_4$, filtered, and concentrated *in vacuo*.

2. The resulting orange oil was dissolved in dry MeOH (150 mL) and refluxed for 3 h. The solvent was removed under reduced pressure. The residue was purified by silica column chromatography (petroleum ether/EtOAc 9:1). Product **a** was obtained as a slightly yellow oil (16.57 g, 82.73 mmol, 67 %). R_f = 0.53 (petrol ether/EtOAc 9:1).

^{1}H-NMR ($CDCl_3$ 400 MHz) δ (ppm): 0.85-0.88 (m, 3H, H-10), 1.22-1.31 (m, 8H, H-6 – H-9), 1.54-1.61 (m, 2H, H-5), 2.52 (t, 2H, *J* = 7.4 Hz, H-4), 3.43 (s, 2H, H-2), 3.72 (s, 3H, O-CH_3).

^{13}C-NMR ($CDCl_3$ 101 MHz) δ (ppm): 14.16 (C-10), 22.70, 29.08, 29.12, 31.75 (C-9 – C-6), 23.59 (C-5), 43.20 (C-4), 49.13 (C-2), 52.42 (O-CH_3), 167.84 (C-1), 202.98 (C-3).

ESI-HRMS: m/z = 223.1305 $[M+Na]^+$, calc. for $C_{11}H_{20}O_3 + Na^+$ = 223.1305.

Methyl 3-hydroxydecanoate (**b**)

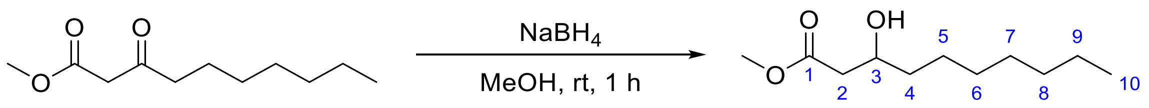

Methyl 3-oxodecanoate (**a**) (4.07 g, 20.30 mmol) was dissolved in dry MeOH (25 mL) and added slowly to a suspension of $NaBH_4$ (921.0 mg, 24.36 mmol) in dry MeOH (60 mL). The obtained mixture was stirred for 1 h at room temperature and quenched with 1 M aq. HCl solution. MeOH was evaporated and the remaining water mixture was extracted with diethyl ether 2 times. The combined organic phases were washed with brine solution, dried over $MgSO_4$, filtered, and concentrated *in vacuo*. The residue was purified by silica column chromatography (petrol ether/EtOAc 3:1) to yield product **b** as a colorless oil (m = 3.30 g, 16.30 mmol, 80 %). R_f = 0.58 (petrol ether/EtOAc 3:1).

^{1}H-NMR ($CDCl_3$ 400 MHz) δ (ppm): 0.86-0.89 (m, 3H, H-10), 1.21-1.37 (m, 10H, H-5 – H-9), 1.39-1.47 (m, 2H, H-4), 2.41 (dd, 1H, *J* = 16.3, 9.2 Hz, H-2), 2.51 (dd, 1H, *J* = 16.3, 3.2 Hz, H-2), 3.71 (s, 3H, O-CH_3), 3.97-4.03 (m, 1H, H-3).

^{13}C-NMR ($CDCl_3$ 101 MHz) δ (ppm): 14.22 (C-10), 22.78, 25.62, 29.36, 29.61, 31.93 (C-9 – C-5), 36.67 (C-4), 41.24 (C-2), 51.87 (O-CH_3), 68.18 (C-3), 173.67 (C-1).

ESI-HRMS: m/z = 225.1460 $[M+Na]^+$, calc. for $C_{11}H_{22}O_3 + Na^+$ = 225.1462.

Methyl 3-((*tert*-butyldimethylsilyl)oxy)decanoate (**1a**)

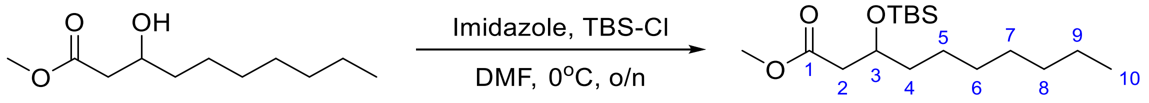

To the solution of methyl 3-hydroxydecanoate (**b**) (619.0 mg, 3.06 mmol) in DMF (2.5 mL) were added imidazole (417.0 mg, 6.12 mmol) and TBS-Cl (554.0 mg, 3.67 mmol) at room temperature. The mixture was stirred overnight at 0˚C. The resulting reaction mixture was poured into sat. aq. NH_4Cl solution and extracted with diethyl ether. The combined organic layers were washed with brine solution and dried over $MgSO_4$. The organic phase was evaporated to dryness to yield product **1a** as a colorless oil (m = 851.0 mg, 2.69 mmol, 88 %). Compound **1a** was used in the next step without further purification.

^{1}H-NMR ($CDCl_3$ 400 MHz) δ (ppm): 0.03 (s, 3H, Si-CH_3), 0.06 (s, 3H, Si-CH_3), 0.86 (s, 9H, $C(CH_3)_3$), 0.86-0.90 (m, 3H, H-10), 1.22-1.36 (m, 10H, H-5 – H-9), 1.45-1.51 (m, 2H, H-4), 2.43 (dd, 2H, *J* = 6.3, 1.8 Hz, H-2), 3.66 (s, 3H, O-CH_3), 4.09-4.15 (m, 1H, H-3).

^{13}C-NMR ($CDCl_3$ 101 MHz) δ (ppm): (-4.69) (Si-CH_3), (-4.36) (Si-CH_3), 14.23 (C-10), 18.14 (**C**$(CH_3)_3$), 22.79, 25.11, 29.39, 29.77, 31.94 (C-9 – C-5), 25.92 (C(**C**$H_3)_3$), 37.80 (C-4), 42.70 (C-2), 51.56 (O-CH_3), 69.68 (C-3), 172.52 (C-1).

ESI-HRMS: m/z = 317.2508 $[M+H]^+$, calc. for $C_{17}H_{36}O_3Si + H^+$ = 317.2507.

Methyl 3-(1,3-dioxolane)decanoate (**2a**)

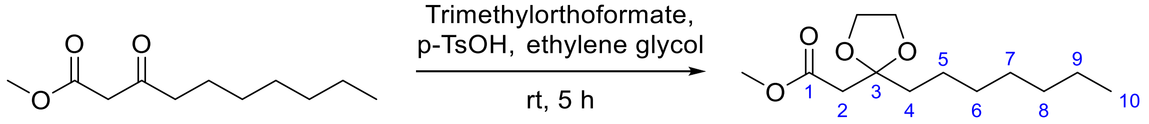

Compound **2a** was synthesized based on the procedure described by Hodgkinson *et. al.* [4].
Methyl 3-oxodecanoate (**a**) (1.39 g, 6.94 mmol) was mixed with *para*-toluene sulfonic acid (132.0 mg, 694.0 µmol), trimethylorthoformate (1.37 mL, 12.49 mmol), and ethylene glycol (1.55 ml, 27.76 mmol) at room temperature under nitrogen atmosphere. The resulting mixture was stirred for 5 h at room temperature under nitrogen atmosphere. 10 mL of 5% aq. Na_2HPO_4 solution was added to the mixture and allowed to stir for 15 min. 15 mL of diethyl ether was added and the mixture was stirred vigorously for 20 min. Organic and aqueous phases were separated. The organic phase was washed with sat. aq. $NaHCO_3$ solution 3 times, dried over $MgSO_4$, filtered, and concentrated *in vacuo*. Product **2a** was obtained as a slightly yellow oil (m = 1.46 g, 5.98 mmol, 85.9 %) and used in the next step without further purification.

^{1}H-NMR ($CDCl_3$ 400 MHz) δ (ppm): 0.87 (m, 3H, H-10), 1.20-1.33 (m, 8H, H-6 – H-9), 1.38 (m, 2H, H-5), 1.78 (m, 2H, H-4), 2.66 (s, 2H, H-2), 3.68 (s, 3H, CH_3), 3.93-4.01 (m, 4H, O-CH_2-CH_2-O).

^{13}C-NMR ($CDCl_3$ 101 MHz) δ (ppm): 14.21 (C-10), 22.77, 29.36, 29.78, 31.91 (C-6 – C-9), 23.65 (C-5), 37.90 (C-4), 42.58 (C-2), 51.86 (O-CH_3), 65.25 (O-CH_2-CH_2-O), 109.57 (C-3), 170.18 (C-1).

General procedure 1: Generation of aldehydes

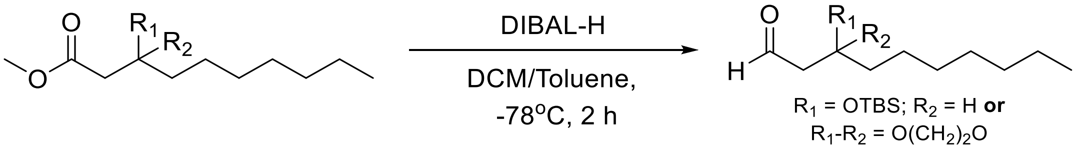

Protected methyl ester (1 eq.) was dissolved in dry DCM (4 mL/1 mmol) and cooled to -78°C. DIBAL-H solution (1 M in toluene, 1.15 eq.) was slowly added and the resulting mixture was stirred at -78°C for 2 h. The reaction was quenched by the addition of MeOH (1.5 mL/1 mmol) and sat. Rochelle salt solution (7 mL/1 mmol). The mixture was allowed to reach room temperature. Organic and aqueous layers were separated. The aqueous phase was extracted 2 times with DCM. The combined organic layers were washed with brine solution and dried over $MgSO_4$. The organic phase was evaporated to dryness. The residue was purified by silica column chromatography (petrol ether/EtOAc 3:1) to yield the product**.**

3-((*tert*-Butyldimethylsilyl)oxy)decanal (**1b**)

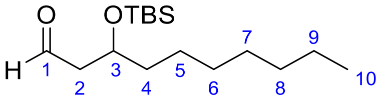

Compound **1b** was synthesized according to the general procedure 1 using 786.5 mg (2.48 mmol) of **1a** and 2.86 mL (2.86 mmol) of DIBAL-H solution. Product **1b** was obtained as a colorless oil (m = 593.4 mg, 2.07 mmol, 83 %) and used in the next step without further purification.

^{1}H-NMR ($CDCl_3$ 400 MHz) δ (ppm): 0.05 (s, 3H, Si-CH_3), 0.07 (s, 3H, Si-CH_3), 0.87 (s, 9H, $C(CH_3)_3$), 0.88 (m, 3H, H-10), 1.24-1.33 (m, 10H, H-5 – H-9), 1.46-1.52 (m, 2H, H-4), 2.50-2.52 (m, 2H, H-2), 4.17 (m, 1H, H-3), 9.81 (t, 1H, *J* = 2.5 Hz, H-1).

^{13}C-NMR ($CDCl_3$ 101 MHz) δ (ppm): (-4.55) (Si-CH_3), (-4.27) (Si-CH_3), 14.23 (C-10), 18.14 (**C**$(CH_3)_3$), 22.78, 25.28, 29.36, 29.71, 31.93 (C-9 – C-5), 25.91 (C(**C**$H_3)_3$), 38.01 (C-4), 50.98 (C-2), 68.46 (C-3), 202.69 (C-1).

3-(1,3-Dioxolane)decanal (**2b**)

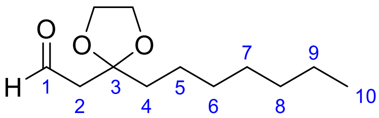

Compound **2b** was synthesized according to the general procedure 1 using 280.0 mg (1.15 mmol) of **2a** and 1.32 mL (1.32 mmol) of DIBAL-H solution. The residue was purified by silica column chromatography (petrol ether/EtOAc 3:1) to yield product **2b** as a colorless oil (m = 502.6 mg, 2.34 mmol, 71.5 %). R_f = 0.54 (petrol ether/EtOAc 3:1).

^{1}H-NMR ($CDCl_3$ 400 MHz) δ (ppm): 0.87 (m, 3H, H-10), 1.23-1.30 (m, 8H, H-6 – H-9), 1.33-1.39 (m, 2H, H-5), 1.67 (m, 2H, H-4), 2.67 (d, 2H, *J* = 2.9 Hz, H-2), 3.99 (m, 4H, O-CH_2-CH_2-O), 9.72 (t, 1H, *J* = 3.0 Hz, H-1).

^{13}C-NMR ($CDCl_3$ 101 MHz) δ (ppm): 14.19 (C-10), 22.75, 29.31, 29.75, 31.87 (C-6 – C-9), 23.67 (C-5), 38.64 (C-4), 50.76 (C-2), 65.20 (O-CH_2-CH_2-O), 109.68 (C-3), 200.79 (C-1).

ESI-HRMS: m/z = 451.3034 $[2M+Na]^+$, calc. for $2C_{24}H_{44}O_6 + Na^+$ = 451.3031.

General procedure 2: Aldol condensation

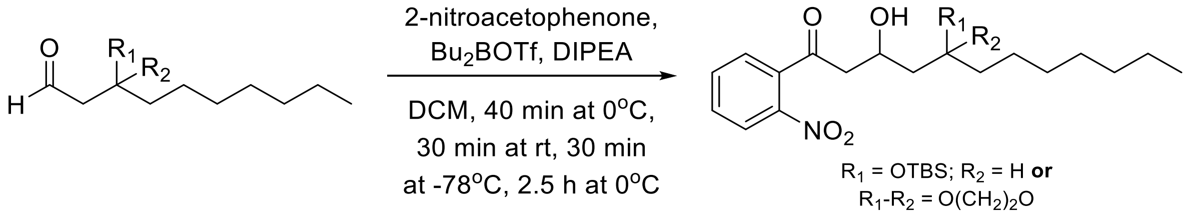

2-Nitroacetophenone (1.15 eq.) was dissolved in dry DCM (4.9 mL/1 mmol of an aldehyde) and cooled to 0°C. Bu_2BOTf solution (1 M in DCM, 2.05 eq.) was added and the mixture stirred for 10 min at 0°C. DIPEA (2.25 eq.) was added as a solution in dry DCM (0.4 mL/1 mmol) and the resulting dark-red mixture was stirred for 30 min at 0°C. The reaction was warmed to room temperature and stirred for 30 min. The mixture was cooled to -78°C and aldehyde (1 eq.) was added slowly as a solution in dry DCM (2.33 mL/1 mmol). The mixture was stirred at -78°C for 30 min, warmed to 0°C, and stirred for 2.5 h at this temperature. The reaction was quenched at 0°C by the addition of pH 7 phosphate buffer (9.3 mL/1 mmol), MeOH (14 mL/1 mmol), and 30% H_2O_2 (9.3 mL/1 mmol). This mixture was allowed to reach room temperature and stirred vigorously for 5 min. Organic and aqueous phases were separated and the aqueous layer was extracted 2 times with DCM. The combined extracts were washed successively with sat. aq. NH_4Cl solution and brine solution. The organic phase was dried over $MgSO_4$ and concentrated *in vacuo*. The residue was purified by silica column chromatography (petrol ether/EtOAc 2:1) to yield the product.

5-((*tert*-Butyldimethylsilyl)oxy)-3-hydroxy-1-(2'-nitrophenyl)dodecan-1-one (**1c**)

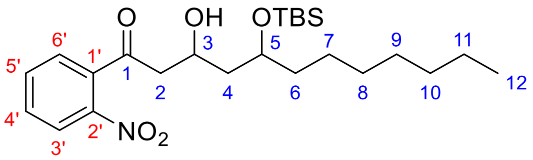

Compound **1c** was synthesized according to the general procedure 2 using 298.0 µL (2.22 mmol) of 2-nitroacetophenone, 3.96 ml (3.96 mmol) of Bu_2BOTf solution, 757.0 µL (4.35 mmol) of DIPEA, and 553.4 mg (1.93 mmol) of aldehyde **1b**. Product **1c** was obtained as an orange oil (m = 680.8 mg, 1.51 mmol, 78.0 %). R_f = 0.55 (petrol ether/EtOAc 2:1).
^{1}H-NMR (MeOH-d_4 400 MHz) δ (mixture of 2 diastereomers (9:11), ppm): 0.06-0.09 (m, 6H, $Si(CH_3)_2$), 0.88 (s, 9H, $C(CH_3)_3$), 0.94 (m, 3H, H-12), 1.24-1.42 (m, 10H, H-7 – H-11), 1.45-1.56 (m, 2H, H-6), 1.59-1.64 (m, 1H, H-4), 1.65-1.77 (m, 1H, H-4), 2.92-3.10 (m, 2H, H-2), 3.91 (p, 0.45H, *J* = 5.9 Hz, H-5), 3.98 (p, 0.55H, *J* = 5.7 Hz, H-5), 4.29 (ddt, 1H, *J* = 15.6, 12.3, 6.7 Hz, H-3), 7.61 (dt, 1H, *J* = 7.6, 1.3 Hz, H-6'), 7.69 (td, 1H, *J* = 7.9, 1.5 Hz, H-4'), 7.81 (tt, 1H, *J* = 7.6, 1.4 Hz, H-5'), 8.12 (dd, 1H, *J* = 8.2, 1.2 Hz, H-3').
^{13}C-NMR (MeOH-d_4 101 MHz) δ (mixture of 2 diastereomers (9:11), ppm): (-4.37), (-4.26), (-4.15), (-4.06) ($Si(CH_3)_2$), 14.42 (C-12), 18.89, 18.93 (**C**$(CH_3)_3$), 23.71, (25.92, 26.03), (30.39, 30.40), (30.84, 30.88), 32.96 (C-11 – C-7), 26.40, 26.45 (C(**C**$H_3)_3$), 37.96, 39.11 (C-6), 45.02, 45.51 (C-4), 51.29, 51.95 (C-2), 65.91, 66.46 (C-3), 70.66, 71.28 (C-5), 125.26, 125.38 (C-3'), 129.15, 129.21 (C-6'), 132.06, 132.10 (C-4'), 135.34 (C-5'), 138.71 (C-1'), 138.89 (C-2'), 202.87, 202.95 (C-1).
ESI-HRMS: m/z = 474.2648 $[M+Na]^+$, calc. for $C_{24}H_{41}NO_5Si + Na^+$ = 474.2647.

5-(1,3-Dioxolane)-3-hydroxy-1-(2'-nitrophenyl)dodecan-1-one (**2c**)

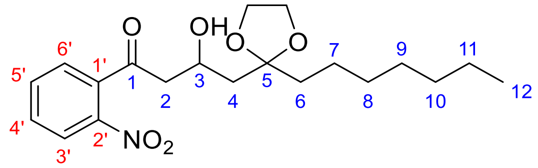

Compound **2c** was synthesized according to the general procedure 2 using 362.0 µL (2.70 mmol) of 2-nitroacetophenone, 4.81 mL (4.81 mmol) of Bu_2BOTf solution, 919.0 µL (5.28 mmol) of DIPEA, and 502.6 mg (2.35 mmol) of aldehyde **2b**. Product **2c** was obtained as a yellow oil (m = 722.0 mg, 1.90 mmol, 81.1 %). R_f = 0.31 (petrol ether/EtOAc 2:1).
^{1}H-NMR (MeOH-d_4 400 MHz) δ (ppm): 0.88-0.92 (m, 3H, H-12), 1.27-1.34 (m, 8H, H-8 – H-11), 1.34-1.41 (m, 2H, H-7), 1.64-1.67 (m, 2H, H-6), 1.87 (dd, 1H, *J* = 14.5, 7.1 Hz, H-4), 1.93 (dd, 1H, *J* = 14.5, 4.9 Hz, H-4), 2.98 (dd, 1H, *J* = 16.7, 7.9 Hz, H-2), 3.13 (dd, 1H, *J* = 16.7, 4.5 Hz, H-2), 3.92-3.96 (m, 4H, O-CH_2-CH_2-O), 4.36 (ddt, 1H, *J* = 7.9, 7.1, 4.7 Hz, H-3), 7.62 (dd, 1H, *J* = 7.6, 1.4 Hz, H-6'), 7.69 (ddd, 1H, *J* = 8.2, 7.4, 1.4 Hz, H-4'), 7.81 (td, 1H, *J* = 7.5, 1.1 Hz, H-5'), 8.11 (dd, 1H, *J* = 8.3, 1.1 Hz, H-3').
^{13}C-NMR (MeOH-d_4 101 MHz) δ (ppm): 14.42 (C-12), 23.71, 30.38, 30.92, 32.97 (C-8 – C-11), 24.84 (C-7), 38.55 (C-6), 44.07 (C-4), 51.36 (C-2), 65.62 (C-3), 65.70 (O-CH_2-CH_2-O), 112.21 (C-5), 125.33 (C-3'), 129.20 (C-6'), 132.08 (C-4'), 135.30 (C-5'), 137.39 (C-1'), 138.82 (C-2'), 202.96 (C-1).
ESI-HRMS: m/z = 402.1878 $[M+Na]^+$, calc. for $C_{20}H_{29}NO_6 + Na^+$ = 402.1888.

General procedure 3: Alcohol oxidation

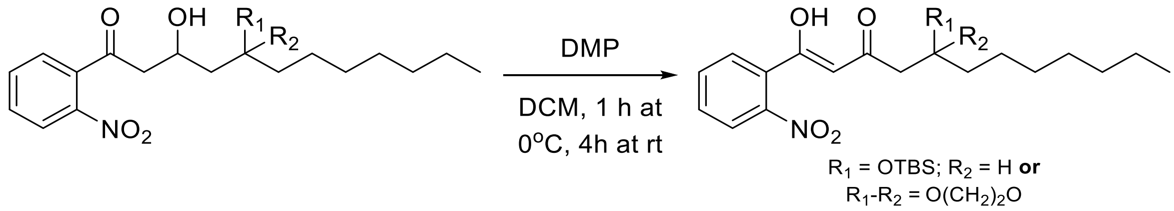

Alcohol (1 eq.) and DMP (1.5 eq.) were dissolved in dry DCM (4.5 mL/1 mmol of alcohol) under nitrogen atmosphere at 0˚C. The resulting mixture was stirred under nitrogen atmosphere for 1 h at 0˚C and for 4 h at room temperature. The mixture was diluted with DCM, washed with sat. aq. $Na_2S_2O_3$ solution and distilled water. The organic phase was dried over $MgSO_4$ and concentrated *in vacuo*. The residue was purified by prep. RP-HPLC (A: H_2O, B: MeOH; 5% B to 100% B) (SPOT PREP Liquid Chromatography by Armen Instrument; Kinetex 5µm C18 100 Å LC Column 250 x 21.2 mm, AXIA).

5-((*tert*-Butyldimethylsilyl)oxy)-3-oxo-1-(2'-nitrophenyl)dodecan-1-one (**1d**)

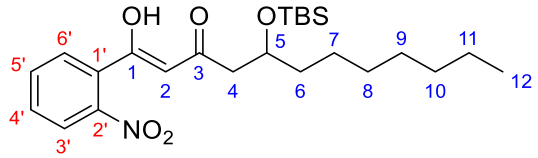

Compound **1d** was synthesized according to the general procedure 3 using 248.0 mg (549.1 µmol) of alcohol **1c** and 349.3 mg (823.6 µmol) of DMP. The product was obtained as an orange oil (m = 113.8 mg, 253.1 µmol, 46.1 %).
^{1}H-NMR (DMSO-d$_6$ 400 MHz) δ (mixture of enol-/keto-tautomers (4:1), ppm): (-0.03) – 0.05 (m, 6H, Si(CH$_3$)$_2$), 0.82 (s, 9H, C(CH$_3$)$_3$), 0.77-087 (m, 3H, H-12), 1.19-1.33 (m, 10H, H-7 – H-11), 1.46-1.51 (m, 2H, H-6), 2.44-2.70 (m, 2H, H-4), 4.10-4.17 (m, 1H, H-5), 4.21 (d, 0.4H, *J* = 2.7 Hz, H-2, keto-from), 6.26 (s, 0.8 H, H-2, enol-form), 7.71-7.89 (m, 3H, H-4'-H-6'), 8.00-8.15 (m, 1H, H-3').
^{13}C-NMR (DMSO-d$_6$ 101 MHz) δ (mixture of enol-/keto-tautomers (4:1), ppm): (-5.01), (-4.67) (Si(CH$_3$)$_2$), 13.90 (C-12), 17.67 (**C**(CH$_3$)$_3$), 22.02, 24.14, 28.54, 28.92, 31.10 (C-11 – C-7), 25.63 (C(**C**H$_3$)$_3$), 37.18 (C-6), 45.10 (C-4), 55.56 (C-2, keto-form, visible in HSQC-NMR), 69.53 (C-5), 100.86 (C-2, enol-form), 124.40 (C-3'), 129.27, 131.07, 133.21 (C-4' – C-6'), 147.93 (C-1'), 186.06, 189.75 (C-1, C-3).
ESI-HRMS: m/z = 450.2664 [M+H]$^+$, calc. for $C_{24}H_{39}NO_5Si + H^+$ = 450.2671.

5-(1,3-Dioxolane)-3-oxo-1-(2'-nitrophenyl)dodecan-1-one (**2d**)

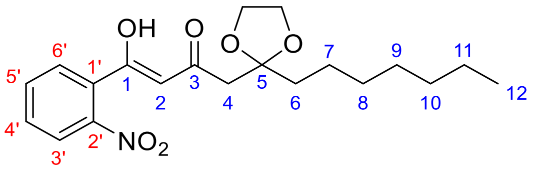

Compound **2d** was synthesized according to the general procedure 3 using 123.5 mg (325.5 µmol) of alcohol **2c** and 207.1 mg (488.2 µmol) of DMP. The product was obtained as an orange oil (m = 60.9 mg, 161.4 µmol, 49.6 %).
^{1}H-NMR (DMSO-d$_6$ 400 MHz) δ (mixture of enol-/keto-tautomers (7:3), ppm): 0.82-086 (m, 3H, H-12), 1.17-1.37 (m, 10H, H-7 – H-11), 1.54-1.69 (m, 2H, H-6), (2.71, 2.81) (s, 2H, H-4), 3.84-3.96 (m, 4H, O-CH$_2$-CH$_2$-O), 4.23 (s, 0.6H, H-2, keto-form, partially in exchange with solvent), 6.20 (s, 0.7H, H-2, enol-form), 7.64-7.95 (m, 3H, H-4'-H-6'), 7.95-8.13 (m, 1H, H-3').
^{13}C-NMR (DMSO-d$_6$ 101 MHz) δ (mixture of enol-/keto-tautomers (7:3), ppm): 13.92 (C-12), 22.05, 22.89, 28.59, 29.05, 31.16 (C-11 – C-7), 37.39 (C-6), 44.91 (C-4, enol form), 49.59 (C-4, keto-form), 55.70 (C-2, keto-form), 64.50 (O-CH$_2$-CH$_2$-O), 101.48 (C-2, enol-form), 109.38 (C-5), 124.44 (C-3'), 129.33, 132.36, 133.42 (C-4'-C-6'), 147.79 (C-1'), 186.04, 188.77 (C-1, C-3).
ESI-HRMS: m/z = 400.1725 [M+Na]$^+$, calc. for $C_{20}H_{27}NO_6 + Na^+$ = 400.1731.

General procedure 4: Reductive cyclization to form 4-quinolone-*N*-oxide

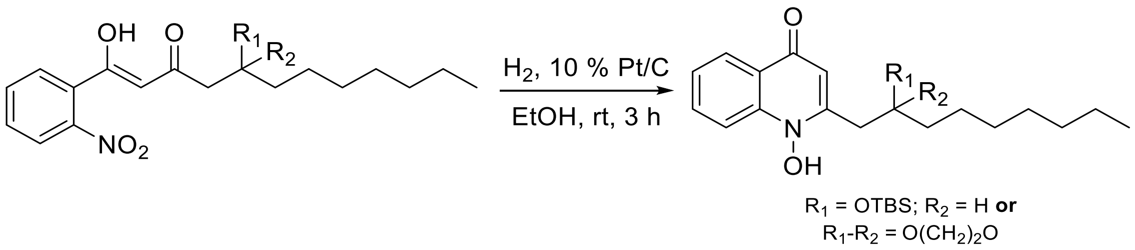

Ketone was dissolved in EtOH (42 mL/1 mmol) and 10 % Pt/C catalyst (0.3 mg/1 mg of ketone) was added. The resulting mixture was placed under nitrogen atmosphere and hydrogen gas was supplied from a H$_2$-filled balloon. The mixture was allowed to stir for 3 h at room

temperature. The catalyst was removed by filtration and the filtrate was evaporated to dryness. The residue was purified by silica column chromatography to yield the product**.**

2-(2′-((*tert*-Butyldimethylsilyl)oxy)nonyl)-1-hydroxyquinolin-4(1*H*)-one (2′-OTBS-NQNO) (**1f**)

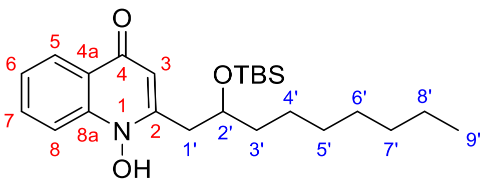

Compound **1f** was synthesized according to the general procedure 4 using 26.4 mg (58.7 µmol) of ketone **1d** and 8.3 mg of 10 % Pt/C catalyst. The residue was purified by silica column chromatography with EtOAc to yield product **1f**, with compounds **1h** and **1e** as minor impurities (m = 4.5 mg). R_f = 0.20 (EtOAc). The product was used in the next step without further purification.

^{1}H-NMR (MeOH-d_4 400 MHz) δ (ppm): (-0.33) (s, 3H, Si-CH_3), (-0.04) (s, 3H, Si-CH_3), 0.82 (s, 9H, C(CH_3)$_3$), 0.90-0.92 (m, 3H, H-9′), 1.29-1.35 (m, 8H, H-5′– H-8′), 1.42-1.52 (m, 2H, H-4′), 1.56-1.66 (m, 2H, H-3′), 2.87 (dd, 1H, *J* = 13.0, 8.5 Hz, H-1′), 3.21 (ddd, 1H, *J* = 15.4, 11.1, 4.0 Hz, H-1′), 4.34 (dq, 1H, *J* = 9.8, 5.3 Hz, H-2′), 6.40 (s, 1H, H-3), 7.53 (ddd, 1H, *J* = 8.3, 6.9, 1.2 Hz, H-6), 7.84 (ddd, 1H, *J* = 8.7, 6.9, 1.6 Hz, H-7), 8.14 (dd, 1H, *J* = 8.7, 4.2 Hz, H-8), 8.27 (dd, 1H, *J* = 8.2, 1.5 Hz, H-5).

^{13}C-NMR (MeOH-d_4 101 MHz) δ (ppm): (-4.90) (Si-CH_3), (-4.53) (Si-CH_3), 14.41 (C-9′), 18.76 (**C**(CH_3)$_3$), 23.70, 30.37, 30.73, 32.93 (C-5′ – C-8′), 25.99 (C-4′), 26.30 (C(**C**H_3)$_3$), 39.23 (C-3′), 41.28 (C-1′), 71.45 (C-2′), 110.13 (C-3), 116.99 (C-8), 125.77 (C-5), 126.29 (C-6), 133.67 (C-7), 141.96 (C-8a), 153.34 (C-2). C-4 is not visible in ^{13}C-NMR for *N*-oxides due to the electronic effects.

ESI-HRMS: m/z = 418.2762 [M+H]$^+$, calc. for $C_{24}H_{39}NO_3Si + H^+$ = 418.2772.

2-(2′-(1,3-Dioxolane)-1-hydroxyquinolin-4(1*H*)-one (2′-(1,3-dioxolane)-NQNO) (**2f**)

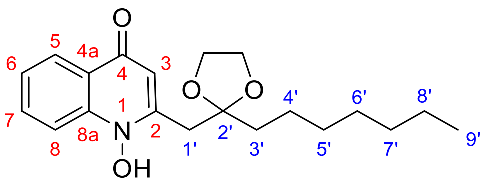

Compound **2f** was synthesized according to the general procedure (described above) using 61.6 mg (163.2 µmol) of ketone **2d** and 19.0 mg of 10 % Pt/C catalyst. The residue was purified by silica column chromatography with MeOH/EtOAc (1:9) to yield product **2f** as a slightly yellow solid (m = 31.4 mg, 90.9 µmol, 55.7 %). Rf = 0.40 (methanol/EtOAc 1:9).

^{1}H-NMR (MeOH-d_4 399.79 MHz) δ (ppm): 0.86-0.91 (m, 3H, H-9′), 1.24-1.34 (m, 8H, H-5′ – H-8′), 1.42-1.48 (m, 2H, H-4′), 1.72-1.76 (m, 2H, H-3′), 3.38 (s, 2H, H-1′), 3.79-3.85 (m, 2H, O-CH_2), 3.88-3.95 (m, 2H, O-CH_2), 6.53 (s, 1H, H-3), 7.51 (ddd, 1H, *J* = 8.2, 7.0, 1.1 Hz, H-6), 7.80 (ddd, 1H, *J* = 8.6, 7.0, 1.5 Hz, H-7), 8.14 (dt, 1H, *J* = 8.7, 0.8 Hz, H-8), 8.26 (ddd, 1H, *J* = 8.2, 1.5, 0.6 Hz, H-5).

^{13}C-NMR (MeOH-d_4 100.52 MHz) δ (ppm): 14.40 (C-9′), 23.69, 30.36, 30.80, 32.93 (C-5′ – C-8′), 24.61 (C-4′), 38.98 (C-1′), 39.39 (C-3′), 66.42 (O-CH_2-CH_2-O), 110.13 (C-3), 112.15 (C-2′),

117.26 (C-8), 125.51 (C-4a), 125.82 (C-5), 126.22 (C-6), 133.59 (C-7), 141.80 (C-8a), 151.45 (C-2). C-4 is not visible in ^{13}C-NMR for *N*-oxides due to the electronic effects. ESI-HRMS: m/z = 346.2003 $[M+H]^+$, calc. for $C_{20}H_{27}NO_4 + H^+$ = 346.2013.

General procedure 5: Reductive cyclization to form 4-quinolone

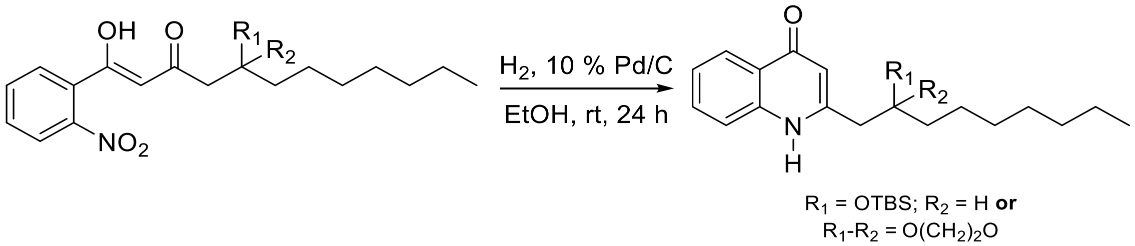

The ketone was dissolved in EtOH (42 mL/1 mmol) and 10 % Pd/C catalyst (0.3 mg/1 mg of ketone) was added. The resulting mixture was placed under nitrogen atmosphere and hydrogen gas was supplied from a H_2-filled balloon. The mixture was allowed to stir for 24 h at room temperature. The reaction mixture was filtered from the catalyst and evaporated to dryness. The residue was purified by silica column chromatography to yield the product**.**

2-(2'-((*tert*-Butyldimethylsilyl)oxy)nonyl)quinolin-4(1*H*)-one (2'-OTBS-NQ) (**1e**)

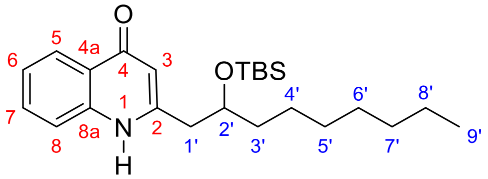

Compound **1e** was synthesized according to the general procedure 5 using 45.8 mg (101.9 µmol) of ketone **1d** and 14.4 mg of 10 % Pd/C catalyst. The residue was purified by silica column chromatography with EtOAc to yield product **1e** as a white solid (m = 24.1 mg, 60.0 µmol, 58.9 %). R_f = 0.56 (EtOAc).
^{1}H-NMR (MeOH-d$_4$ 400 MHz) δ (ppm): (-0.35) (s, 3H, Si-CH_3), (-0.02) (s, 3H, Si-CH_3), 0.77 (s, 9H, $C(CH_3)_3$), 0.87-0.93 (m, 3H, H-9'), 1.29-1.38 (m, 8H, H-5'-H-8'), 1.42-1.50 (m, 2H, H-4'), 1.55-1.67 (m, 2H, H-3'), 2.71 (dd, 1H, *J* = 13.5, 9.4 Hz, H-1'), 2.93 (dd, 1H, *J* = 13.5, 3.5 Hz, H-1'), 4.13 (dq, 1H, *J* = 9.6, 5.3 Hz, H-2'), 6.26 (s, 1H, H-3), 7.40 (ddd, 1H, *J* = 8.1, 6.9, 1.1 Hz, H-6), 7.57 (d, 1H, *J* = 8.3 Hz, H-8), 7.69 (ddd, 1H, *J* = 8.5, 7.0, 1.5 Hz, H-7), 8.22 (dd, 1H, *J* = 8.2, 1.5 Hz, H-5).
^{13}C-NMR (MeOH-d$_4$ 101 MHz) δ (ppm): (-4.97) (Si-CH_3), (-4.44) (Si-CH_3), 14.44 (C-9'), 18.73 (**C**$(CH_3)_3$), 23.73, 30.38, 30.84, 32.95 (C-5' – C-8'), 25.73 (C-4'), 26.21 (C(**C**$H_3)_3$), 39.29 (C-3'), 42.69 (C-1'), 73.68 (C-2'), 110.27 (C-3), 119.11 (C-8), 125.15 (C-6), 125.64 (C-4a), 126.00 (C-5), 133.38 (C-7), 141.48 (C-8a), 154.94 (C-2), 180.45 (C-4).
ESI-HRMS: m/z = 402.2813 $[M+H]^+$, calc. for $C_{24}H_{39}NO_2Si + H^+$ = 402.2823.

2-(2'-(1,3-Dioxolane))quinolin-4(1*H*)-one (2'-(1,3-dioxolane)-NQ (**2e**)

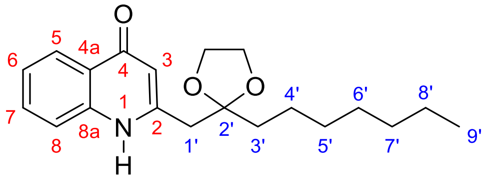

Compound **2e** was synthesized according to the general procedure 5 using 17.5 mg (46.4 µmol) of ketone **2d** and 6.0 mg of 10 % Pd/C catalyst. The residue was purified by silica column chromatography MeOH/EtOAc 5:95 to yield product **2e** as a white solid (m = 11.8 mg, 35.8 µmol, 77.3 %). R_f = 0.32 (MeOH/EtOAc 5:95).
^{1}H-NMR (MeOH-d_4 400 MHz) δ (ppm): 0.87-0.90 (m, 3H, H-9'), 1.24-1.34 (m, 8H, H-5'-H-8'), 1.41-1.49 (m, 2H, H-4'), 1.67-1.71 (m, 2H, H-3'), 3.03 (s, 2H, H-1'), 3.73-3.81 (m, 2H, O-CH_2), 3.87-3.95 (m, 2H, O-CH_2), 6.33 (s, 1H, H-3), 7.40 (ddd, 1H, *J* = 8.2, 6.8, 1.2 Hz, H-6), 7.62-7.64 (m, 1H, H-8), 7.70 (ddd, 1H, *J* = 8.4, 6.8, 1.5 Hz, H-7), 8.22 (dd, 1H, *J* = 8.3, 1.4 Hz, H-5).
^{13}C-NMR (MeOH-d_4 101 MHz) δ (ppm): 14.39 (C-9'), 24.68 (C-4'), 23.68, 30.34, 30.80, 32.92 (C-5' – C-8'), 39.48 (C-3'), 43.00 (C-1'), 66.52 (O-CH_2-CH_2-O), 111.67 (C-3), 119.26 (C-8), 125.17 (C-6), 125.55 (C-4a), 125.99 (C-5), 133.40 (C-7), 141.51 (C-8a), 151.85 (C-2), 180.21 (C-4).
ESI-HRMS: m/z = 330.2053 $[M+H]^+$, calc. for $C_{20}H_{27}NO_3 + H^+$ = 330.2064.

General procedure 6: TBS-deprotection

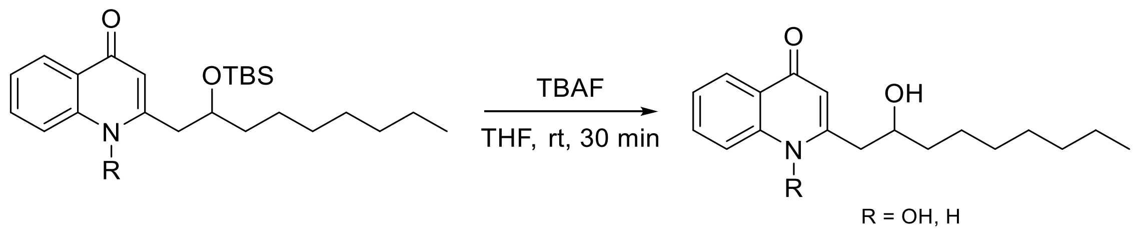

Starting material was dissolved in dry THF (18.5 mL/1 mmol) under nitrogen atmosphere. 1 M TBAF solution in THF (18.5 mL/1 mmol) was added and the mixture was allowed to stir at room temperature for 30 min. Then THF was evaporated under reduced pressure. The residue was dissolved in EtOAc and washed with distilled water 3 times. The organic phase was dried over $MgSO_4$ and concentrated *in vacuo*. The residue was purified by prep. RP-HPLC (A: H_2O, B: MeOH; 5% B to 95% B) to yield the product.

1-Hydroxy-2-(2'-hydroxynonyl)quinolin-4(1*H*)-one (2'-OH-NQNO) (**1h**)

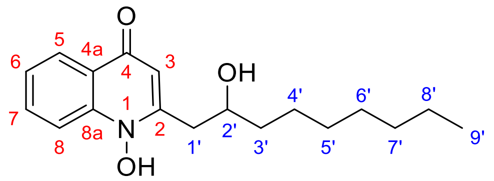

Compound **1h** was synthesized according to the general procedure 6 using 4.5 mg (10.8 µmol) of starting material **1f** and 0.2 ml of 1 M TBAF solution in THF. The product was obtained as a slightly yellow solid (m = 2.9 mg, 9.6 µmol, 88.7 %).
^{1}H-NMR (MeOH-d_4 400 MHz) δ (ppm): 0.87-0.92 (m, 3H, H-9'), 1.25-1.45 (m, 10H, H-4' – H-8'), 1.55-1.62 (m, 2H, H-3'), 2.97 (dd, 1H, *J* = 13.8, 8.7 Hz, H-1'), 3.19 (dd, 1H, *J* = 13.8, 3.9 Hz, H-1'), 4.10 (dddd, 1H, *J* = 8.9, 7.0, 5.3, 3.9 Hz, H-2'), 6.46 (s, 1H, H-3), 7.53 (ddd, 1H, *J* = 8.1, 7.0, 1.1 Hz, H-6), 7.83 (ddd, 1H, *J* = 8.6, 7.0, 1.5 Hz, H-7), 8.16 (d, 1H, *J* = 8.7 Hz, H-8), 8.27 (dd, 1H, *J* = 8.2, 1.5 Hz, H-5).
^{13}C-NMR (MeOH-d_4 101 MHz) δ (ppm): 14.41 (C-9'), 23.71, 30.41, 30.63, 32.98 (C-5'– C-8'), 26.72 (C-4'), 38.77 (C-3'), 40.71 (C-1'), 71.08 (C-2'), 109.07 (C-3), 117.13 (C-8), 125.21 (C-4a), 125.71 (C-5), 126.29 (C-6), 133.63 (C-7), 141.79 (C-8a), 153.40 (C-2), 172.09 (C-4) (visible in HSQC-NMR).
ESI-HRMS: m/z = 304.1896 $[M+H]^+$, calc. for $C_{18}H_{25}NO_3 + H^+$ = 304.1908.

2-(2′-Hydroxynonyl)quinolin-4(1*H*)-one (2′-OH-NQ) (**1g**)

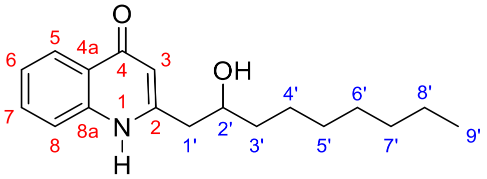

Compound **1g** was synthesized according to the general procedure (described above) using 13.5 mg (33.6 µmol) of starting material **1e** and 0.3 ml of 1 M TBAF solution in THF. The product was obtained as a white solid (m = 8.0 mg, 27.8 µmol, 82.8 %).
^{1}H-NMR (MeOH-d$_4$ 400 MHz) δ (ppm): 0.88-0.91 (m, 3H, H-9′), 1.26-1.44 (m, 10H, H-4′ – H-8′), 1.49-1.59 (m, 2H, H-3′), 2.74 (dd, 1H, *J* = 14.0, 8.6 Hz, H-1′), 2.88 (dd, 1H, *J* = 14.0, 4.3 Hz, H-1′), 3.96 (tt, 1H, *J* = 8.7, 3.7 Hz, H-2′), 6.28 (s, 1H, H-3), 7.39 (ddd, 1H, *J* = 8.1, 6.9, 1.1 Hz, H-6), 7.60 (m, 1H, H-8), 7.69 (ddd, 1H, *J* = 8.4, 6.9, 1.5 Hz, H-7), 8.22 (dd, 1H, *J* = 8.2, 1.5 Hz, H-5).
^{13}C-NMR (MeOH-d$_4$ 101 MHz) δ (ppm): 14.40 (C-9′), 23.71, 30.40, 30.64, 32.98 (C-5′ – C-8′), 26.73 (C-4′), 38.50 (C-3′), 43.01 (C-1′), 71.64 (C-2′), 110.12 (C-3), 119.20 (C-8), 125.09 (C-6), 125.60 (C-4a), 125.97 (C-5), 133.36 (C-7), 141.51 (C-8a), 154.55 (C-2), 180.52 (C-4).
ESI-HRMS: m/z = 288.1951 [M+H]$^+$, calc. for $C_{18}H_{25}NO_2 + H^+$ = 288.1959.

General procedure 7: Ketal-deprotection

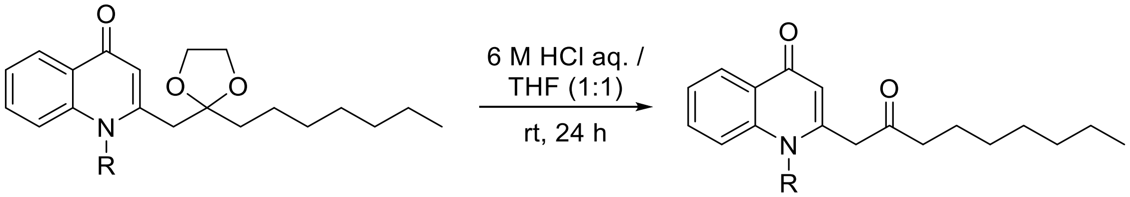

Starting material was dissolved in THF/6 M HCl aq. solution (1:1) (46.5 mL/1 mmol). The resulting mixture was allowed to stir at room temperature for 24 h. The reaction mixture was diluted with distilled water and extracted 2 times with EtOAc. The combined organic layers were washed with sat. aq. $NaHCO_3$ and Brine solutions. The organic phase was dried over $MgSO_4$ and concentrated *in vacuo*. The residue was purified by prep. RP-HPLC or by silica column chromatography to yield the product.

1-Hydroxy-2-(2′-oxononyl)quinolin-4(1*H*)-one (2′-oxo-NQNO) (**2h**)

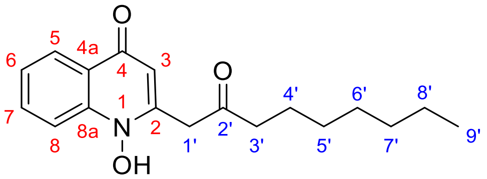

Compound **2h** was synthesized according to the general procedure 7 using 28.3 mg (85.9 µmol) of starting material **2f** and 4.0 ml of THF/6 M HCl aq. solution (1:1) mixture. The residue was purified by prep. RP-HPLC (A: H_2O, B: MeOH; 5% B to 95% B) (SPOT PREP Liquid Chromatography by Armen Instrument; Kinetex 5 µm C18 100 Å LC Column 250 x 21.2 mm, AXIA) to yield product **2h** as a slightly yellow solid (m = 12.7 mg, 44.5 µmol, 52 %).

^{1}H-NMR (MeOH-d$_4$ 400 MHz) δ (ppm): 0.89-0.92 (m, 3H, H-9'), 1.27-1.42 (m, 8H, H-5' – H-8'), 1.60-1.67 (m, 2H, H-4'), 2.42-2.61 (bs, 2H, H-3'), 6.40 (s, 1H, H-3), 7.52 (t, 1H, *J* = 7.7 Hz, H-6), 7.82 (ddd, 1H, *J* = 8.5, 7.0, 1.4 Hz, H-7), 7.95-8.09 (bs, 1H, H-8), 8.29 (dd, 1H, *J* = 8.3, 1.4 Hz, H-5).
^{13}C-NMR (MeOH-d$_4$ 101 MHz) δ (ppm): 14.33 (C-9'), 23.63, 30.17, 30.29, 32.85 (C-5' – C-8'), 24.94 (C-4') (visible in HSQC-NMR), 43.59 (C-3') (visible in HSQC-NMR), 125.38, 126.07 (C-5 – C-6), 133.70 (C-7), C-4 is not visible in ^{13}C-NMR for *N*-oxides due to the electronic effects.

Note: Signals H-1', C-2, C-3, C-4, C-4a, C-7, C-8, C-8a, C-1', and C-2' couldn't be detected by NMR using different NMR-solvents (MeOH-d$_4$, DMSO-d$_6$, MeCN-d$_3$) at room temperature or 40˚C. Low solubility of the compound in common solvents prevented an adequate concentration for measurements.

ESI-HRMS: m/z = 302.1739 [M+H]$^+$, calc. for $C_{18}H_{23}NO_3 + H^+$ = 302.1751.

2-(2'-Oxononyl)quinolin-4(1*H*)-one (2'-oxo-NQ) (**2g**)

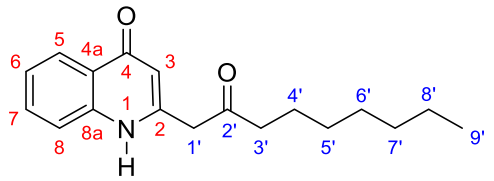

Compound **2g** was synthesized according to the general procedure 7 using 11.6 mg (35.2 µmol) of starting material **2e** and 3.0 mL of THF/6 M HCl aq. solution (1:1) mixture. The residue was purified by silica column chromatography with MeOH/EtOAc (5:95) to yield product **2g** as a white solid (m = 4.8 mg, 16.8 µmol, 47.8 %). R$_f$ = 0.29 (MeOH/EtOAc 5:95).
^{1}H-NMR (MeOH-d$_4$ 400 MHz) δ (in keto-form, ppm): 0.89-0.93 (m, 3H, H-9'), 1.26-1.37 (m, 8H, H-5' – H-8'), 1.60-1.66 (m, 2H, H-4'), 2.64 (t, 2H, *J* = 7.3 Hz, H-3'), 6.23 (s, 1H, H-3), 7.41 (ddd, 1H, *J* = 8.1, 7.0, 1.1 Hz, H-6), 7.56 (dt, 1H, *J* = 8.4, 0.8 Hz, H-8), 7.70 (ddd, 1H, *J* = 8.4, 7.0, 1.5 Hz, H-7), 8.23 (ddd, 1H, *J* = 8.2, 1.5, 0.6 Hz, H-5). H-1' is in exchange with solvent.
^{13}C-NMR (MeOH-d$_4$ 101 MHz) δ (in keto-form, ppm): 14.38 (C-9'), 24.58 (C-4'), 23.66, 30.14, 30.20, 32.86 (C-5'- C-8'), 43.51 (C-3'), 47.36 (C-1') (visible in HMBC-NMR), 110.87 (C-3), 119.18 (C-8), 125.21 (C-6), 125.57 (C-4a), 126.04 (C-5), 133.57 (C-7), 141.67 (C-8a), 149.40 (C-2), 180.70 (C-4), 206.74 (C-2').
ESI-HRMS: m/z = 286.1793 [M+H]$^+$, calc. for $C_{18}H_{23}NO_2 + H^+$ = 286.1802.

β-Oxodecanoic acid (**3a**)

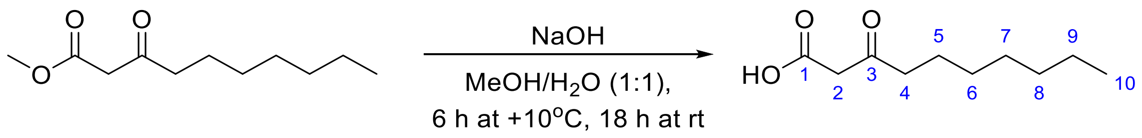

Methyl ester **a** (617.0 mg, 3.08 mmol) was dissolved in 4.4 mL of MeOH at +10˚C. 4.4 mL of 0.7 M aqueous NaOH solution (3.08 mmol) was added. The resulting mixture was allowed to stir at +10˚C for 6 h and at room temperature for 18 h. MeOH was evaporated under reduced pressure (≤+30˚C for water bath) and the residue was diluted with cooled water (5˚C). The resulting mixture was acidified to pH 1-2 with 1 M HCl and product **3a** precipitated. The

product was separated from water by filtration, dried under reduced pressure, and obtained as a white solid (m = 461.7 mg, 2.48 mmol, 80.5 %).
^{1}H-NMR ($CDCl_3$ 400 MHz) δ (mixture of enol-/keto-tautomers (1:5), ppm): 0.86-0.90 (m, 3H, H-10), 1.21-1.36 (m, 8H, H-6 - H-9), 1.57-1.66 (m, 2H, H-5), 2.23 (t, 2H, *J* = 7.6 Hz, H-4, enol-form), 2.56 (t, 2H, *J* = 7.4 Hz, H-4, keto-form), 3.52 (s, 2H, H-2, keto-form), 5.04 (s, 1H, H-2, enol-form), 11.86 (s, 1H, COOH).
^{13}C-NMR ($CDCl_3$ 101 MHz) δ (ppm): 14.02 (C-10), 22.56, 28.90, 28.94, 31.58 (C-9 – C-6), 23.36 (C-5), 43.31 (C-4), 46.72 (C-2, keto-form), 87.84 (C-2, enol-form), 169.06 (C-1), 206.11 (C-3).
ESI-HRMS: m/z = 231.0967 $[M\text{-}H\text{+}2Na]^+$, calc. for $C_{10}H_{17}NaO_3 + Na^+$ = 231.0968.

β-Hydroxydecanoic acid (**3b**)

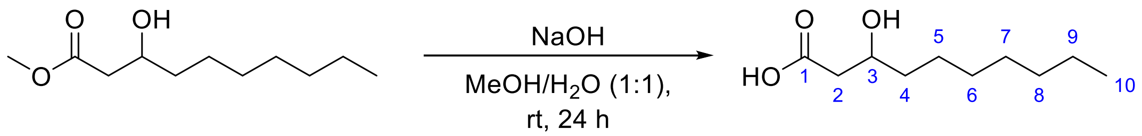

Methyl ester **b** (619.4 mg, 3.06 mmol) was dissolved in 30 mL of MeOH/water (1:1) mixture and NaOH (244.9 mg, 6.12 mmol) was added. The resulting mixture was allowed to stir at room temperature for 24 h. MeOH was evaporated under reduced pressure and the residue was diluted with distilled water. The resulting mixture was acidified to pH 1-2 with 1 M HCl and product **3b** was precipitated at 0˚C. The product was separated from water by filtration, dried under reduced pressure, and obtained as a white solid (m = 382.0 mg, 2.03 mmol, 66.3 %).
^{1}H-NMR (DMSO-d_6 400 MHz) δ (ppm): 0.85-0.87 (m, 3H, H-10), 1.19-1.32 (m, 10H, H-5 – H-9), 1.32-1.39 (m, 2H, H-4), 2.21 (ddd, 1H, *J* = 14.8, 7.9, 1.0 Hz, H-2), 2.29 (ddd, 1H, *J* = 14.8, 5.1, 1.0 Hz, H-2), 3.78 (m, 1H, H-3), 4.57 (bs, 1H, OH), 11.97 (bs, 1H, COOH).
^{13}C-NMR (DMSO-d_6 101 MHz) δ (ppm): 13.95 (C-10), 22.08, 25.05, 28.69, 29.00, 31.25 (C-9 – C-5), 36.90 (C-4), 42.72 (C-2), 67.04 (C-3), 173.02 (C-1).
ESI-HRMS: m/z = 233.1123 $[M\text{-}H\text{+}2Na]^+$, calc. for $C_{10}H_{19}NaO_3 + Na^+$ = 233.1125.

Methyl 3-hydroxydecanoate-d (**3c**)

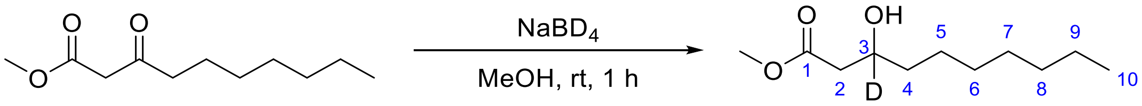

Methyl 3-oxodecanoate (**a**) (1.10 g, 5.49 mmol) was dissolved in dry MeOH (6.8 mL) and added slowly to a suspension of $NaBD_4$ (298.9 mg, 7.14 mmol) in dry MeOH (19.5 mL). The obtained mixture was stirred for 1 h at room temperature and quenched with 1 M aq. HCl solution. MeOH was evaporated and the remaining water mixture was extracted with diethyl ether 2 times. The combined organic phases were washed with brine solution, dried over $MgSO_4$, filtered, and concentrated *in vacuo*. The residue was purified by silica column chromatography (petrol ether/EtOAc 3:1) to yield product **3c** as a colorless oil (m = 497.5 mg, 2.42 mmol, 44 %). R_f = 0.58 (petrol ether/EtOAc 3:1).
^{1}H-NMR ($CDCl_3$ 400 MHz) δ (ppm): 0.85-0.89 (m, 3H, H-10), 1.21-1.54 (m, 12H, H-4 – H-9), 2.37-2.54 (m, 2H, H-2), 3.71 (s, 3H, O-CH_3).

^{13}C-NMR ($CDCl_3$ 101 MHz) δ (ppm): 14.20 (C-10), 22.77, 25.59, 29.35, 29.61, 31.93 (C-9 – C-5), 36.58 (C-4), 41.18 (C-2), 51.84 (O-CH_3), 67.77 (C-3), 173.62 (C-1).
ESI-HRMS: m/z = 226.1524 $[M+Na]^+$, calc. for $C_{11}H_{21}DO_3 + Na^+$ = 226.1524.

β-Hydroxydecanoic acid-d (**3d**)

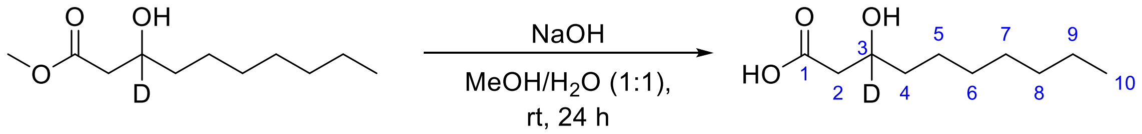

Methyl ester **3c** (98.0 mg, 482 µmol) was dissolved in 5 mL of MeOH/water (1:1) mixture and NaOH (38.6 mg, 964 µmol) was added. The resulting mixture was allowed to stir at room temperature for 24 h. MeOH was evaporated under reduced pressure and the residue was diluted with distilled water. The resulting mixture was acidified to pH 1-2 with 1 M HCl and product **3d** was precipitated at 0˚C. The product was separated from water by filtration, dried under reduced pressure, and obtained as a white solid (m = 61.1 mg, 325 µmol, 67.5 %).
^{1}H-NMR (DMSO-d_6 400 MHz) δ (ppm): 0.84-0.88 (m, 3H, H-10), 1.17-1.39 (m, 12H, H-4 – H-9), 2.24 (q, 2H, *J* = 14.8 Hz, H-2), 4.55 (s, 1H, OH), 11.97 (s, 1H, COOH).
^{13}C-NMR (DMSO-d_6 101 MHz) δ (ppm): 13.89 (C-10), 22.03, 24.97, 28.64, 28.96, 31.20 (C-9 – C-5), 36.75 (C-4), 42.59 (C-2), 66.53 (C-3) (visible in HMBC-NMR), 172.96 (C-1).
ESI-HRMS: m/z = 212.1369 $[M+Na]^+$, calc. for $C_{10}H_{19}DO_3 + Na^+$ = 212.1368.

4. Supplementary Tables

Table S1. Bacterial strains and their growth conditions.

Strain	Growth conditions
Burkholderia ambifaria AMMD	LB medium; 28˚C, 37˚C; 180 rpm
Burkholderia thailandensis DSM13276	LB medium; 28˚C, 37˚C; 180 rpm
Staphylococcus aureus USA300	LB medium; 37˚C; 180 rpm
Pseudomonas aeruginosa PAO1	LB medium; 37˚C; 180 rpm
Pseudomonas aeruginosa PA14	LB medium; 37˚C; 180 rpm

Table S2. Calibration curves for quantification of quinolones. LLOQ = Lower Limit of Quantification. LOD = Limit of Detection (on Dionex Ultimate 3000 UHPLC + Finnigan™ TSQ® Quantum).

Calibration standard	RT	Mass Transitions	Calibration equation; R-value; Concentrations	LLOQ [mg/L]	LOD [mg/L]
2’-OH-NQ	5.71	288/270	Y = 1.68983e+006+7.04084e+006*X; R^2 = 0.9883; C = 0.010, 0.025, 0.050, 0.10, 0.25, 0.50, 0.75, 1.00, 2.50, 5.00, 7.50, 10.00 mg/L	0.010	0.001
2’-OH-NQNO	5.96	304/286	Y = 1.56524e+006+4.63466e+006*X; R^2 = 0.9867; C = 0.025, 0.10, 0.25, 0.50, 0.75, 1.00, 2.50, 5.00, 7.50, 10.00 mg/L	0.025	0.003
NQ	7.65	272/159	Y = 9.49363e+007+1.96682e+008*X; R^2 = 0.9782; C = 0.025, 0.25, 0.75, 1.00, 2.50, 5.00, 7.50, 10.00 mg/L	0.025	0.003
NQNO	7.39	288/159	Y = 3.7495e+007+1.02015e+008*X; R^2 = 0.9852; C = 0.025, 0.25, 0.75, 1.00, 2.50, 5.00, 7.50, 10.00 mg/L	0.025	0.003

Table S3a. Relative abundance of detected fragments of each standard after fragmentation by CID at 30 V (on Dionex Ultimate 3000 UHPLC + Finnigan™ TSQ® Quantum).

Standard	RT	Parental mass	Fragments (m/z and relative abundance)
2’-OH-NQ	5.71	288	159 (100%), 270 (55%), 172 (35%), 184 (15%), 198 (10%)
2’-OH-NQNO	5.96	304	159 (100%), 198 (85%), 286 (75%), 184 (50%), 158 (45%), 172 (30%)
2’-oxo-NQ	6.16	286	160 (100%), 159 (25%)
2’-oxo-NQNO	6.23	302	159 (100%), 186 (50%), 158 (55%)
NQ	7.65	272	159 (70%), 172 (35%)

NQNO	7.39	288	159 (100%), 186 (35%), 172 (25%)
Δ^1-NQ	7.59	270	184 (100%), 159 (30%), 172 (20%), 198 (6%)
Δ^1-NQNO	6.91	286	198 (100%), 184 (76%), 159 (38%), 158 (28%), 172 (22%)
Δ^2-NQ	7.27	270	159 (100%), 172 (28%), 184 (16%), 198 (14%)
Δ^2-NQNO	7.03	286	159 (100%), 158 (88%), 184 (74%), 198 (54%), 172 (50%)

Table S3b. Relative abundance of detected fragments of each standard after fragmentation by CID at 30 V (on Vanquish™ UHPLC + TSQ® Series II Quantum).

**Standard**	**RT**	**Parental mass**	**Fragments (m/z and relative abundance)**
2’-OH-NQ	4.42	288	159 (100%), 270 (37%), 172 (35%), 184 (20%), 198 (15%)
2’-OH-NQNO	4.77	304	159 (100%), 198 (80%), 286 (65%), 184 (45%), 158 (40%), 172 (32%), 240 (22%), 226 (15%)
2’-oxo-NQ	4.89	286	160 (100%), 159 (15%)
2’-oxo-NQNO	5.04	302	159 (100%), 186 (62%), 158 (38%), 214 (20%), 201 (15%), 176 (12%)
NQ	5.99	272	159 (100%), 172 (42%)
NQNO	6.01	288	159 (100%), 186 (30%), 172 (20%)
Δ^1-NQ	5.95	270	184 (100%), 159 (25%), 172 (16%), 198 (6%)
Δ^1-NQNO	5.60	286	198 (100%), 184 (68%), 159 (34%), 158 (24%)
Δ^2-NQ	5.79	270	159 (100%), 172 (25%), 184 (15%), 198 (12%)
Δ^2-NQNO	5.75	286	158 (100%), 159 (90%), 184 (70%), 198 (63%), 172 (45%), 212 (18%), 240 (12%)

Table S4. 2’-OH-NQ quantification in *P. aeruginosa* PA14 and PAO1 strains.

**Sample**	**Integrated Area**	**Sample**	**Integrated Area**
PA14-3h-1	84100	PAO1-3h-1	116317
PA14-3h-2	58009	PAO1-3h-2	304891
PA14-3h-3	81001	PAO1-3h-3	191163
PA14-6h-1	791530	PAO1-6h-1	3504122
PA14-6h-2	831066	PAO1-6h-2	3207211
PA14-6h-3	986640	PAO1-6h-3	2095445
PA14-9h-1	2295687	PAO1-9h-1	5219176
PA14-9h-2	2813816	PAO1-9h-2	3629966
PA14-9h-3	2576780	PAO1-9h-3	4213426
PA14-12h-1	3548713	PAO1-12h-1	5528172

PA14-12h-2	5353730	PAO1-12h-2	4497147
PA14-12h-3	6787559	PAO1-12h-3	6622890
PA14-24h-1	3764432	PAO1-24h-1	3821309
PA14-24h-2	3965460	PAO1-24h-2	3845877
PA14-24h-3	3271938	PAO1-24h-3	5088473

Table S5. 2’-OH-NQNO quantification in *P. aeruginosa* PA14 and PAO1 strains.

Sample	Integrated Area	Sample	Integrated Area
PA14-3h-1	18613	PAO1-3h-1	47554
PA14-3h-2	8439	PAO1-3h-2	118025
PA14-3h-3	16051	PAO1-3h-3	72927
PA14-6h-1	153016	PAO1-6h-1	1309690
PA14-6h-2	169866	PAO1-6h-2	988177
PA14-6h-3	287696	PAO1-6h-3	765605
PA14-9h-1	551553	PAO1-9h-1	1945562
PA14-9h-2	449644	PAO1-9h-2	1398121
PA14-9h-3	423467	PAO1-9h-3	1948738
PA14-12h-1	552234	PAO1-12h-1	2410936
PA14-12h-2	544770	PAO1-12h-2	1345731
PA14-12h-3	853214	PAO1-12h-3	2002962
PA14-24h-1	439808	PAO1-24h-1	1282271
PA14-24h-2	586253	PAO1-24h-2	1320051
PA14-24h-3	396548	PAO1-24h-3	1719640

Table S6. NQ quantification in *P. aeruginosa* PA14 and PAO1 strains.

Sample	Integrated Area	Sample	Integrated Area
PA14-3h-1	501848	PAO1-3h-1	366262
PA14-3h-2	496682	PAO1-3h-2	691942
PA14-3h-3	381478	PAO1-3h-3	564122
PA14-6h-1	27292364	PAO1-6h-1	15042567
PA14-6h-2	30610390	PAO1-6h-2	14316099
PA14-6h-3	39152826	PAO1-6h-3	9160270
PA14-9h-1	207887296	PAO1-9h-1	14851050
PA14-9h-2	228071486	PAO1-9h-2	14349381
PA14-9h-3	218898729	PAO1-9h-3	14760414
PA14-12h-1	145790218	PAO1-12h-1	26663682
PA14-12h-2	184756234	PAO1-12h-2	29582702
PA14-12h-3	202022388	PAO1-12h-3	34178842
PA14-24h-1	115574217	PAO1-24h-1	39157521
PA14-24h-2	174106562	PAO1-24h-2	37789855
PA14-24h-3	176192623	PAO1-24h-3	46101755

Table S7. NQNO quantification in *P. aeruginosa* PA14 and PAO1 strains.

Sample	Integrated Area	Sample	Integrated Area
PA14-3h-1	1971240	PAO1-3h-1	3816139

PA14-3h-2	1020809	PAO1-3h-2	9115471
PA14-3h-3	1559278	PAO1-3h-3	5586308
PA14-6h-1	21267448	PAO1-6h-1	105014203
PA14-6h-2	19080779	PAO1-6h-2	65826758
PA14-6h-3	39783465	PAO1-6h-3	56181928
PA14-9h-1	55971590	PAO1-9h-1	130961624
PA14-9h-2	46297781	PAO1-9h-2	104467959
PA14-9h-3	51258513	PAO1-9h-3	142361611
PA14-12h-1	67280119	PAO1-12h-1	217840225
PA14-12h-2	59189333	PAO1-12h-2	143899586
PA14-12h-3	110715899	PAO1-12h-3	178472465
PA14-24h-1	143371405	PAO1-24h-1	311097746
PA14-24h-2	199897873	PAO1-24h-2	321063006
PA14-24h-3	147173304	PAO1-24h-3	430203776

Table S8. 2’-oxo-NQ quantification in *P. aeruginosa* PA14 and PAO1 strains. ND = Not Detected.

Sample	Integrated Area	Sample	Integrated Area
PA14-3h-1	ND	PAO1-3h-1	ND
PA14-3h-2	ND	PAO1-3h-2	ND
PA14-3h-3	ND	PAO1-3h-3	ND
PA14-6h-1	ND	PAO1-6h-1	ND
PA14-6h-2	ND	PAO1-6h-2	ND
PA14-6h-3	ND	PAO1-6h-3	ND
PA14-9h-1	ND	PAO1-9h-1	ND
PA14-9h-2	ND	PAO1-9h-2	ND
PA14-9h-3	ND	PAO1-9h-3	ND
PA14-12h-1	ND	PAO1-12h-1	ND
PA14-12h-2	ND	PAO1-12h-2	ND
PA14-12h-3	ND	PAO1-12h-3	ND
PA14-24h-1	ND	PAO1-24h-1	ND
PA14-24h-2	ND	PAO1-24h-2	ND
PA14-24h-3	ND	PAO1-24h-3	ND

Table S9. 2’-oxo-NQNO quantification in *P. aeruginosa* PA14 and PAO1 strains. ND = Not Detected.

Sample	Integrated Area	Sample	Integrated Area
PA14-3h-1	ND	PAO1-3h-1	ND
PA14-3h-2	ND	PAO1-3h-2	ND
PA14-3h-3	ND	PAO1-3h-3	ND
PA14-6h-1	ND	PAO1-6h-1	ND
PA14-6h-2	ND	PAO1-6h-2	ND
PA14-6h-3	ND	PAO1-6h-3	ND
PA14-9h-1	ND	PAO1-9h-1	ND

PA14-9h-2	ND	PAO1-9h-2	ND
PA14-9h-3	ND	PAO1-9h-3	ND
PA14-12h-1	ND	PAO1-12h-1	ND
PA14-12h-2	ND	PAO1-12h-2	ND
PA14-12h-3	ND	PAO1-12h-3	ND
PA14-24h-1	ND	PAO1-24h-1	ND
PA14-24h-2	ND	PAO1-24h-2	ND
PA14-24h-3	ND	PAO1-24h-3	ND

Table S10. 2’-OH-NQ quantification in *B. ambifaria* and *B. thailandensis*. ND = Not Detected.

Sample	Integrated Area	Sample	Integrated Area
BA-9h-1	ND	BA-18h-2	ND
BA-9h-2	ND	BA-21h-1	ND
BA-11h-1	ND	BA-21h-2	ND
BA-11h-2	ND	BA-24h-1	ND
BA-12h-1	ND	BA-24h-2	ND
BA-12h-2	ND	BT-7h-1	ND
BA-13h-1	ND	BT-7h-2	ND
BA-13h-2	ND	BT-14h-1	ND
BA-14h-1	ND	BT-14h-2	ND
BA-14h-2	ND	BT-16h-1	ND
BA-18h-1	ND	BT-16h-2	ND

Table S11. 2’-OH-NQNO quantification in *B. ambifaria* and *B. thailandensis*. ND = Not Detected.

Sample	Integrated Area	Sample	Integrated Area
BA-9h-1	ND	BA-18h-2	ND
BA-9h-2	ND	BA-21h-1	ND
BA-11h-1	ND	BA-21h-2	ND
BA-11h-2	ND	BA-24h-1	ND
BA-12h-1	ND	BA-24h-2	ND
BA-12h-2	ND	BT-7h-1	ND
BA-13h-1	ND	BT-7h-2	ND
BA-13h-2	ND	BT-14h-1	ND
BA-14h-1	ND	BT-14h-2	ND
BA-14h-2	ND	BT-16h-1	ND
BA-18h-1	ND	BT-16h-2	ND

Table S12. 2’-oxo-NQ quantification in *B. ambifaria* and *B. thailandensis*. ND = Not Detected.

Sample	Integrated Area	Sample	Integrated Area
BA-9h-1	ND	BA-18h-2	ND
BA-9h-2	ND	BA-21h-1	ND
BA-11h-1	ND	BA-21h-2	ND
BA-11h-2	ND	BA-24h-1	ND
BA-12h-1	ND	BA-24h-2	ND

BA-12h-2	ND	BT-7h-1	ND
BA-13h-1	ND	BT-7h-2	ND
BA-13h-2	ND	BT-14h-1	ND
BA-14h-1	ND	BT-14h-2	ND
BA-14h-2	ND	BT-16h-1	ND
BA-18h-1	ND	BT-16h-2	ND

Table S13. 2’-oxo-NQNO quantification in *B. ambifaria* and *B. thailandensis*. ND = Not Detected.

Sample	Integrated Area	Sample	Integrated Area
BA-9h-1	ND	BA-18h-2	ND
BA-9h-2	ND	BA-21h-1	ND
BA-11h-1	ND	BA-21h-2	ND
BA-11h-2	ND	BA-24h-1	ND
BA-12h-1	ND	BA-24h-2	ND
BA-12h-2	ND	BT-7h-1	ND
BA-13h-1	ND	BT-7h-2	ND
BA-13h-2	ND	BT-14h-1	ND
BA-14h-1	ND	BT-14h-2	ND
BA-14h-2	ND	BT-16h-1	ND
BA-18h-1	ND	BT-16h-2	ND

Table S14. 2’-OH-NQ and 2’-OH-NQ + 1 Da; 2’-OH-NQNO and 2’-OH-NQNO + 1 Da quantification in *P. aeruginosa* PAO1 after β-hydroxydecanoic acid-d (**3d**) feeding.

Sample	Integrated Area	Sample	Integrated Area
Control-2’-OH-NQ-1	1440413	Control-2’-OH-NQNO-1	1223304
Control-2’-OH-NQ-2	1884944	Control-2’-OH-NQNO-2	1526312
Control-2’-OH-NQ-3	3333398	Control-2’-OH-NQNO-3	1064222
Control-2’-OH-NQ+1Da-1	299708	Control-2’-OH-NQNO+1Da-1	238723
Control-2’-OH-NQ+1Da-2	358985	Control-2’-OH-NQNO+1Da-2	256667
Control-2’-OH-NQ+1Da-3	500500	Control-2’-OH-NQNO+1Da-3	214404

AfterFeeding-2'-OH-NQ-1	1630731	AfterFeeding-2'-OH-NQNO-1	1368420
AfterFeeding-2'-OH-NQ-2	1687804	AfterFeeding-2'-OH-NQNO-2	1393864
AfterFeeding-2'-OH-NQ-3	1993007	AfterFeeding-2'-OH-NQNO-3	1454276
AfterFeeding-2'-OH-NQ+1Da-1	1604961	AfterFeeding-2'-OH-NQNO+1Da-1	1101990
AfterFeeding-2'-OH-NQ+1Da-2	1500030	AfterFeeding-2'-OH-NQNO+1Da-2	1070906
AfterFeeding-2'-OH-NQ+1Da-3	1784619	AfterFeeding-2'-OH-NQNO+1Da-3	1068054

Table S15. 2'-OH-NQ and 2'-OH-NQNO quantification in *P. aeruginosa* PAO1 after β-hydroxydecanoic acid (**3b**) feeding.

Sample	Integrated Area	Sample	Integrated Area
Control-2'-OH-NQ-1	3609549	Control-2'-OH-NQNO-1	2392019
Control-2'-OH-NQ-2	3603880	Control-2'-OH-NQNO-2	2388870
Control-2'-OH-NQ-3	3441893	Control-2'-OH-NQNO-3	2329880
AfterFeeding50μM-2'-OH-NQ-1	12143160	AfterFeeding50μM -2'-OH-NQNO-1	5480479
AfterFeeding50μM -2'-OH-NQ-2	12823687	AfterFeeding50μM -2'-OH-NQNO-2	6123735
AfterFeeding50μM -2'-OH-NQ-3	11926100	AfterFeeding50μM -2'-OH-NQNO-3	6006622
AfterFeeding100μM-2'-OH-NQ-1	9028906	AfterFeeding100μM-2'-OH-NQNO-1	4635033
AfterFeeding100μM-2'-OH-NQ-2	12358925	AfterFeeding100μM-2'-OH-NQNO-2	5885229

AfterFeeding100µM-2’-OH-NQ-3	12673985	AfterFeeding100µM-2’-OH-NQNO-3	5658018
AfterFeeding150µM-2’-OH-NQ-1	13917867	AfterFeeding150µM-2’-OH-NQNO-1	6378512
AfterFeeding150µM-2’-OH-NQ-2	10679772	AfterFeeding150µM-2’-OH-NQNO-2	4974616
AfterFeeding150µM-2’-OH-NQ-3	11387135	AfterFeeding150µM-2’-OH-NQNO-3	5437844
AfterFeeding200µM-2’-OH-NQ-1	13737617	AfterFeeding200µM-2’-OH-NQNO-1	6219857
AfterFeeding200µM-2’-OH-NQ-2	11347482	AfterFeeding200µM-2’-OH-NQNO-2	5337976
AfterFeeding200µM-2’-OH-NQ-3	9542953	AfterFeeding200µM-2’-OH-NQNO-3	4430976

5. Supplementary Figures

a)

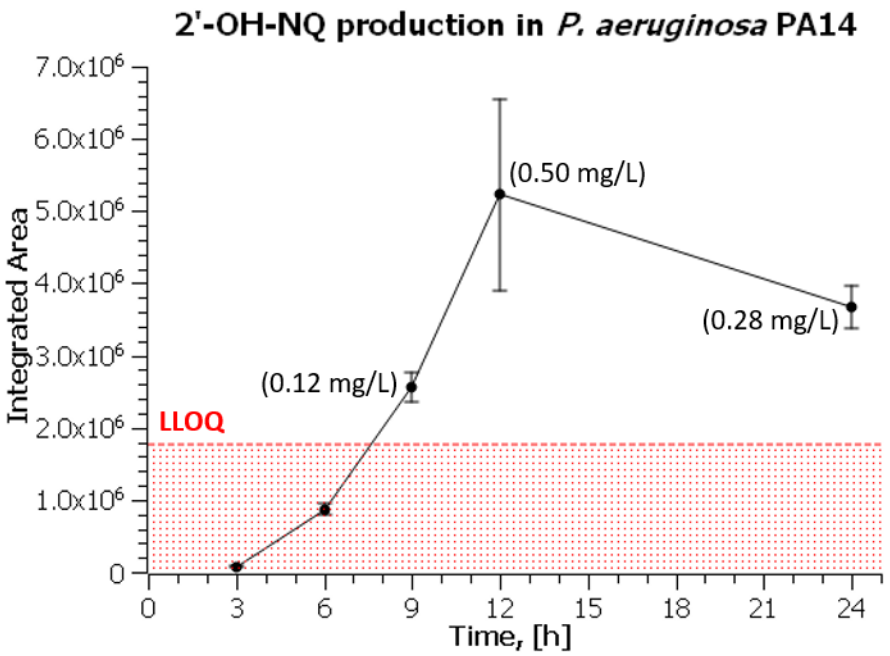

b)

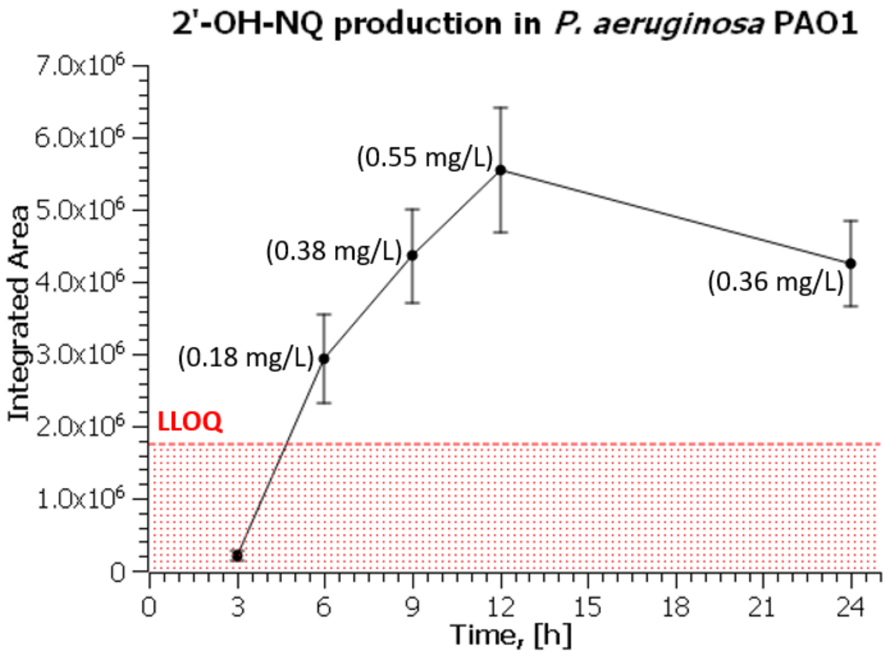

Figure S1. Integrated area of monitored mass transitions of 2’-OH-NQ produced by: **a)** *P. aeruginosa* PA14; **b)** *P. aeruginosa* PAO1 after different incubation times. Quantified concentrations are given in brackets. LLOQ = Lower Limit of Quantification. The error bars represent the population standard deviation.

a)

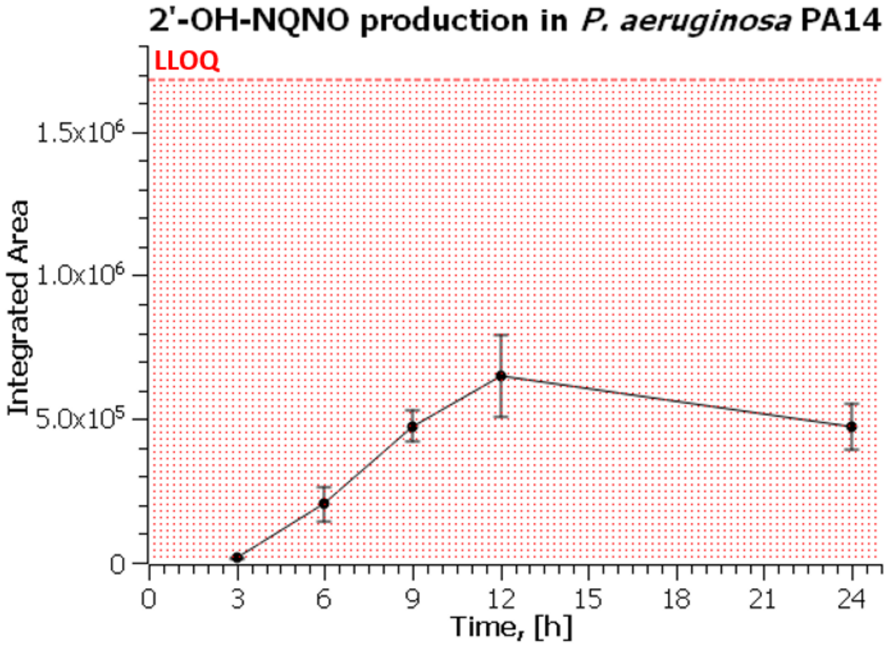

b)

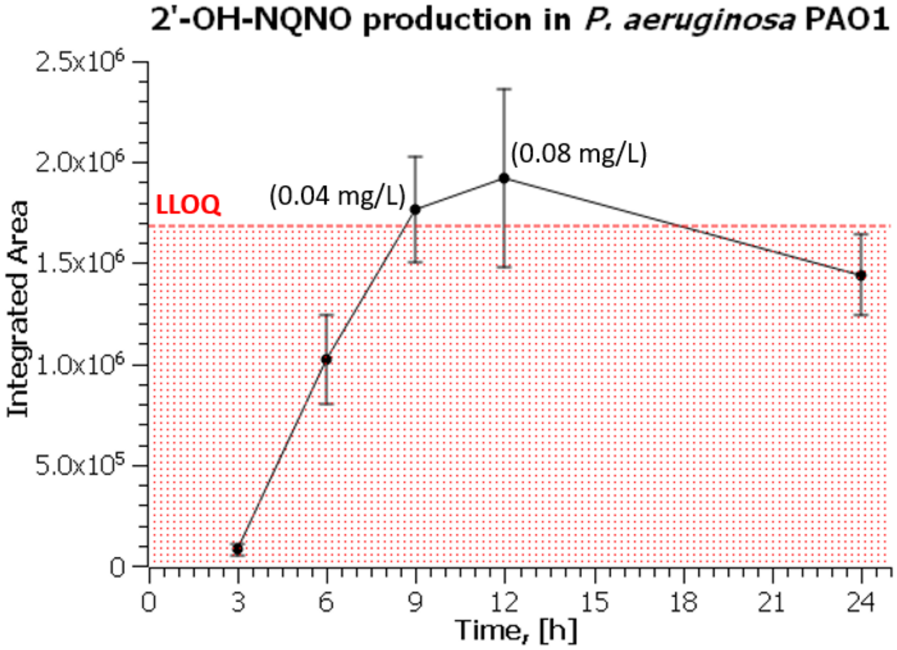

Figure S2. Integrated area of monitored mass transitions of 2'-OH-NQNO produced by: **a)** *P. aeruginosa* PA14; **b)** *P. aeruginosa* PAO1 after different incubation times. Quantified concentrations are shown in brackets. LLOQ = Lower Limit of Quantification. The error bars represent the population standard deviation.

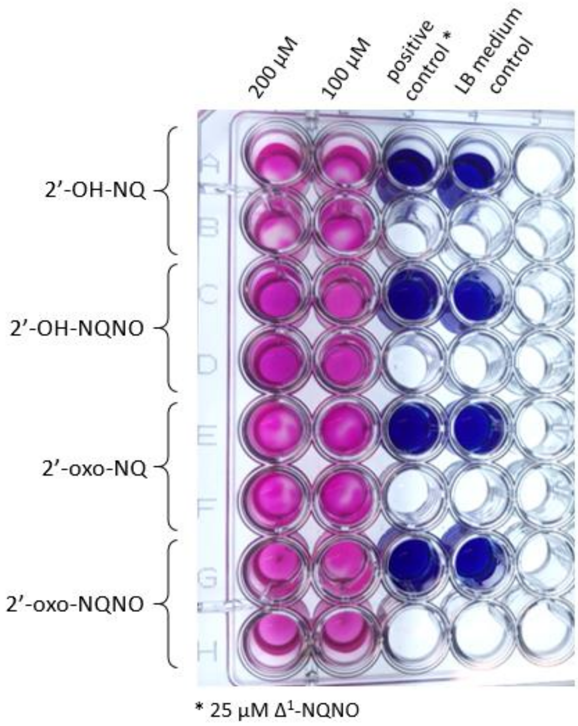

Figure S3. Resazurin assay of *S. aureus* USA300 over an incubation time of 8 h. Δ^1-NQNO = (E)-Δ^1-2-(non-1-enyl)-4(1*H*)-quinolone *N*-oxide.

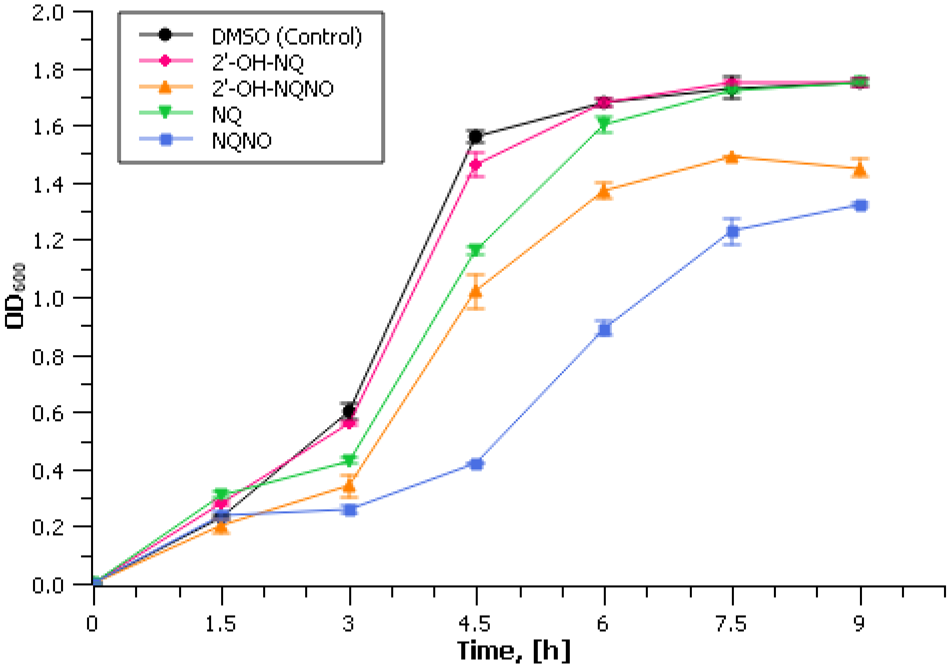

Figure S4. Growth curves of *S. aureus* USA300 in presence of different quinolones (50 µM) and DMSO as a control. The error bars represent the population standard deviation.

a)

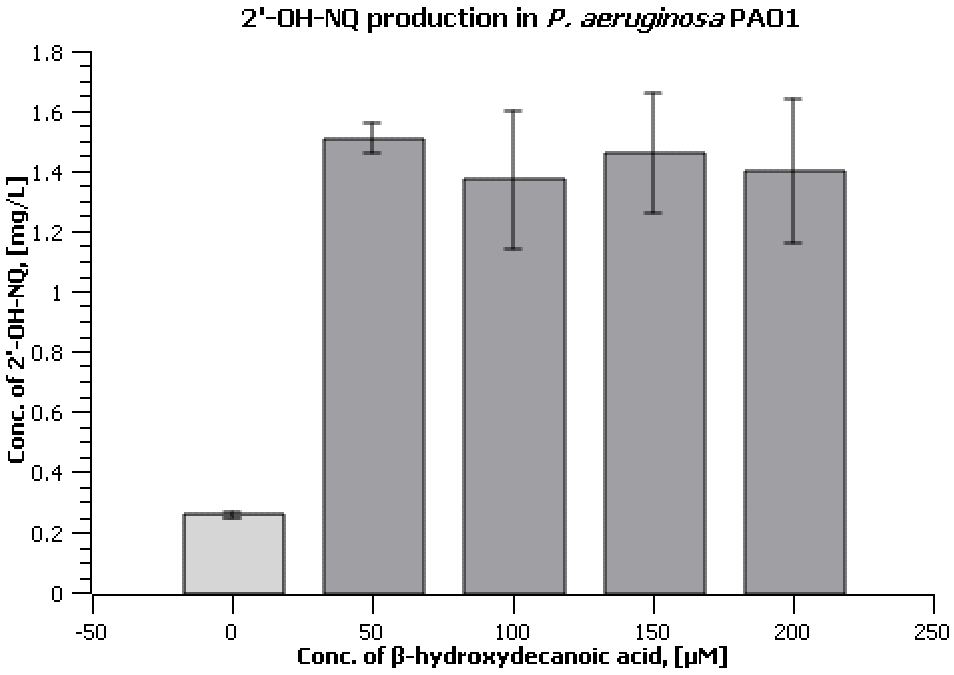

b)

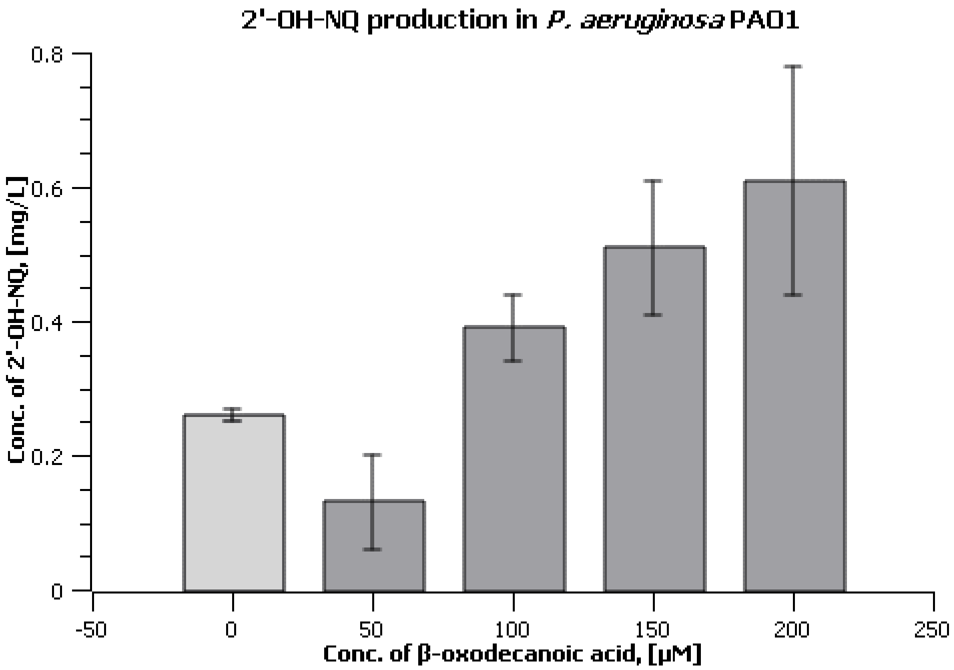

Figure S5. Levels of 2’-OH-NQ production in *P. aeruginosa* PAO1 while feeding:
a) β-hydroxydecanoic acid; **b)** β-oxodecanoic acid to cultures. The error bars represent the population standard deviation.

a)

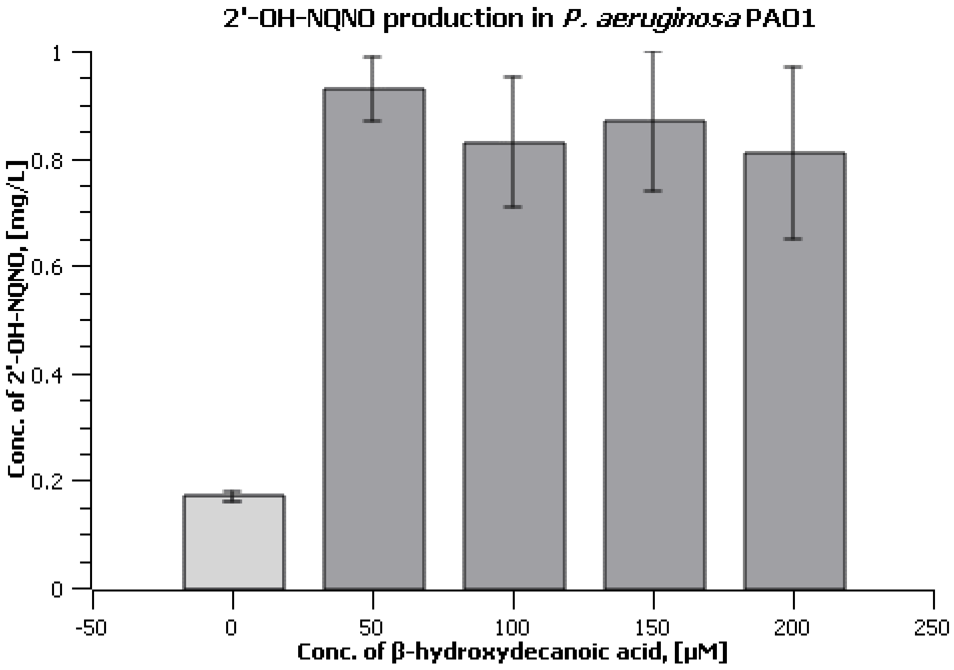

b)

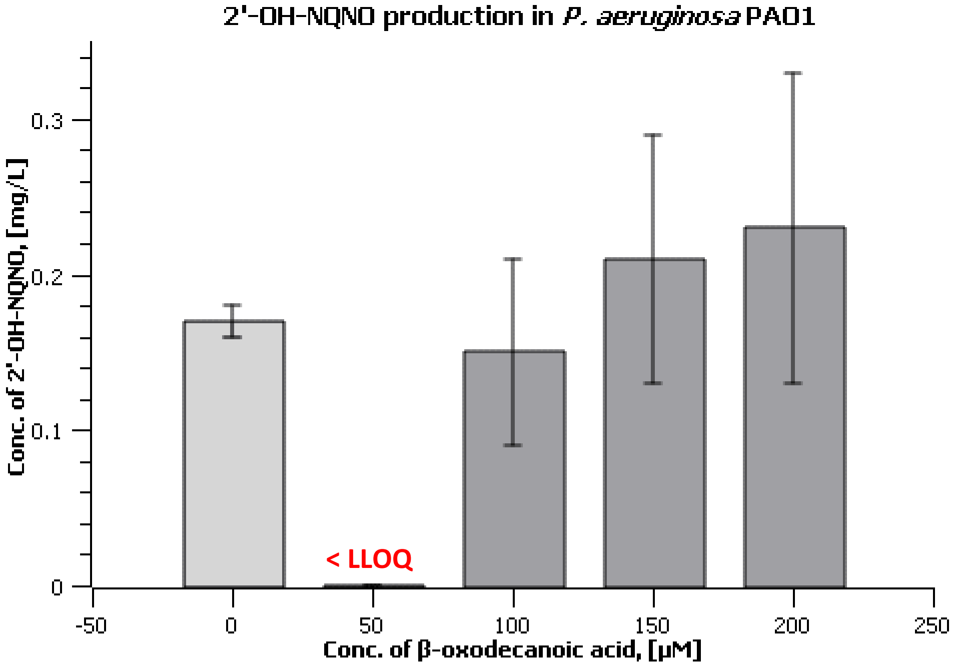

Figure S6. Levels of 2'-OH-NQNO production in *P. aeruginosa* PAO1 while feeding: **a)** β-hydroxydecanoic acid; **b)** β-oxodecanoic acid to cultures. LLOQ = Lower Limit of Quantification. The error bars represent the population standard deviation.

a)

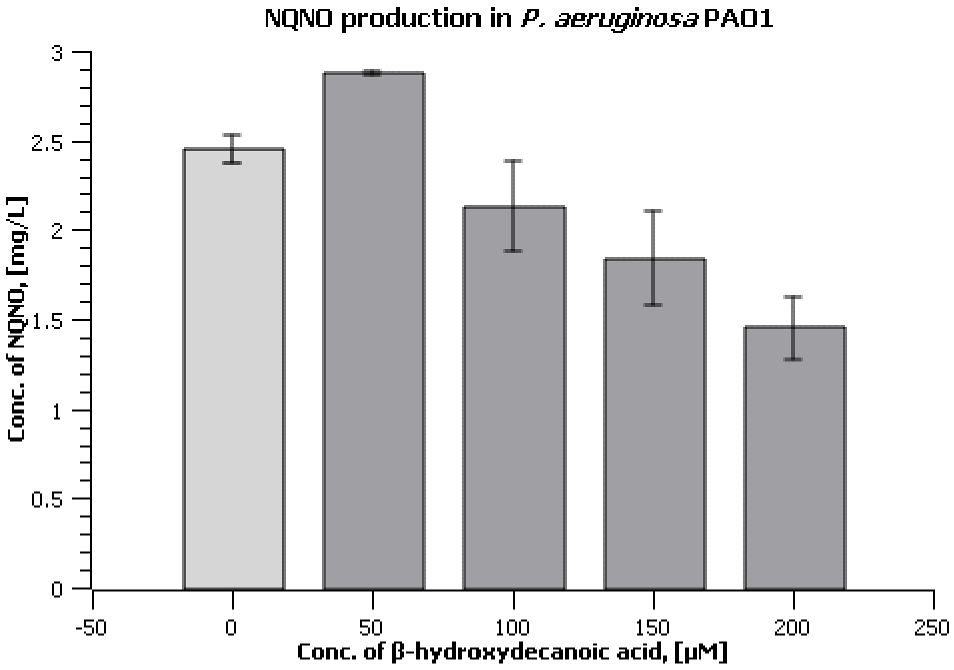

b)

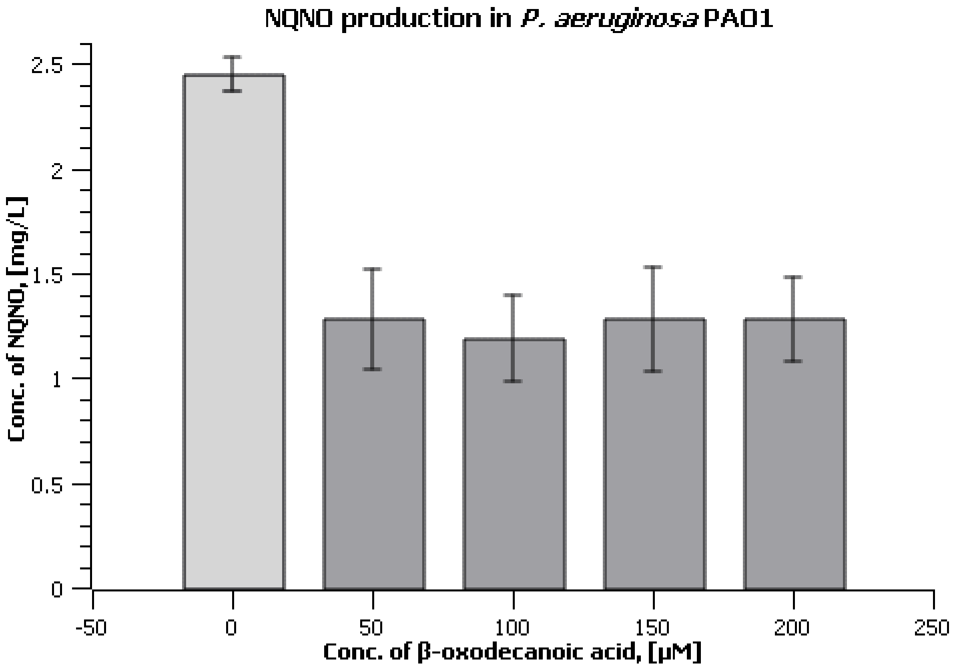

Figure S7. Levels of NQNO production in *P. aeruginosa* PAO1 while feeding:
a) β-hydroxydecanoic acid; **b)** β-oxodecanoic acid to cultures. The error bars represent the population standard deviation.

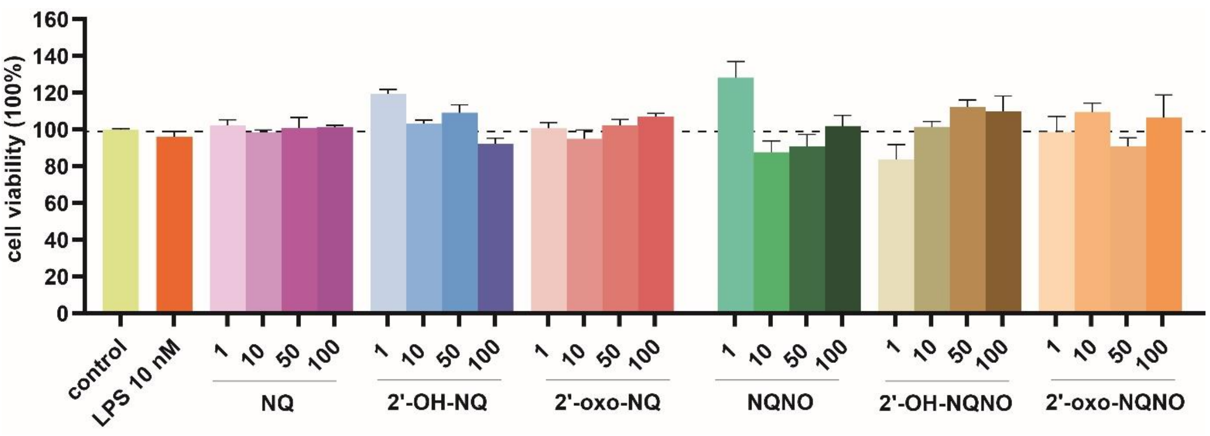

Figure S8. Cell viability of differentiated Caco-2 cells after 24 h of treatment with 10 nM LPS, 1-100 nM NQ, 2’-OH-NQ, 2’-oxo-NQ, NQNO, 2’-OH-NQNO, 2’-oxo-NQNO. Data are presented as Mean± SEM of four to five independent biological repetitions in triplicates.

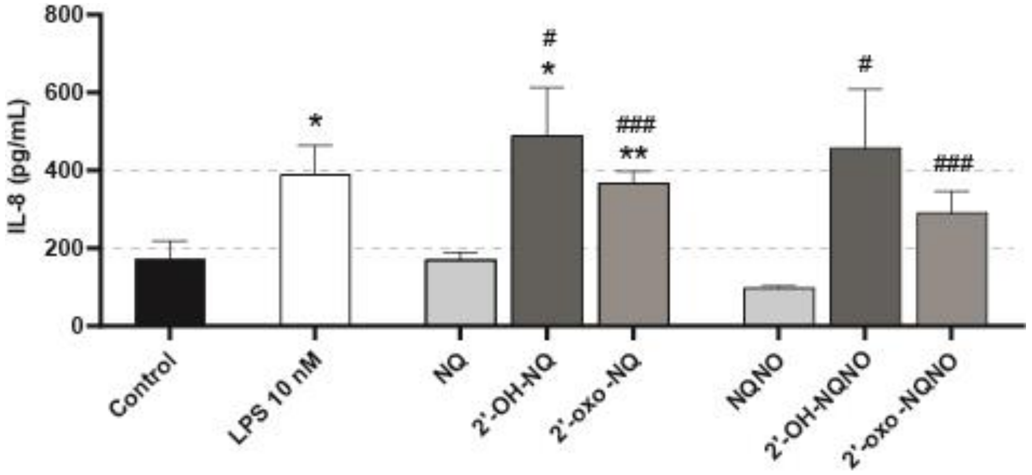

Figure S9. IL-8 release from differentiated Caco-2 cells after 24 h of treatment with medium (Control), 10 nM LPS, 100 nM NQ, 2’-OH-NQ, 2’-oxo-NQ, NQNO, 2’-OH-NQNO, and 2’-oxo-NQNO. Data are presented as Mean ± SEM of four to five independent biological repetitions in triplicates. * ($p < 0.05$) or ** ($p < 0.01$) indicates statistical significance compared with the control group. # ($p < 0.05$) or ### ($p < 0.001$) indicates statistical significance compared with NQ or NQNO. LPS = Lipopolysaccharides.

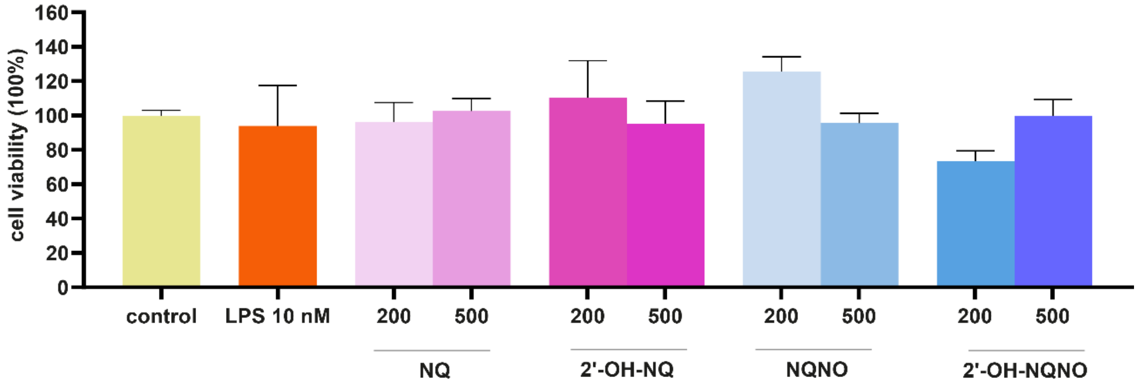

Figure S10. Cell viability of differentiated Caco-2 cells after 24 h of treatment with 10 nM LPS, 200 and 500 nM NQ, 2'-OH-NQ, NQNO, and 2'-OH-NQNO. Data are presented as Mean± SEM of four to five independent biological repetitions in triplicates.

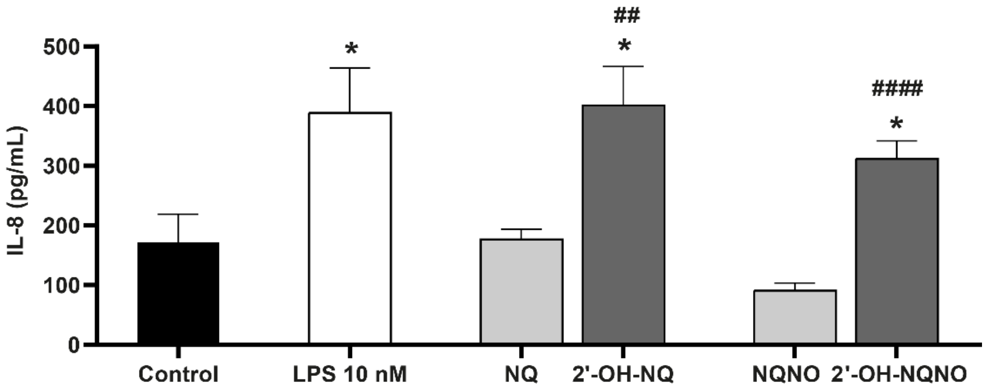

Figure S11. IL-8 release from differentiated Caco-2 cells after 24 h of treatment with medium (Control), 10 nM LPS, 500 nM NQ, 2'-OH-NQ, NQNO, and 2'-OH-NQNO. Data are presented as Mean ± SEM of four to five independent biological repetitions in triplicates. * ($p < 0.05$) or ** ($p < 0.01$) indicates statistical significance compared with the control group. # ($p < 0.05$) or ### ($p < 0.001$) indicates statistical significance compared with NQ or NQNO. LPS = Lipopolysaccharides.

a)

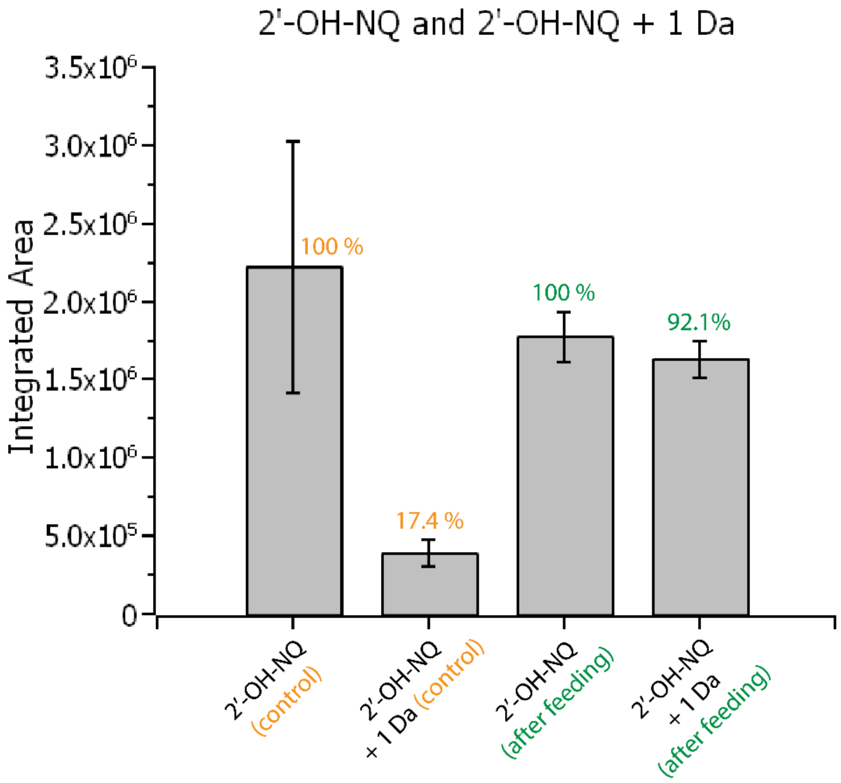

b)

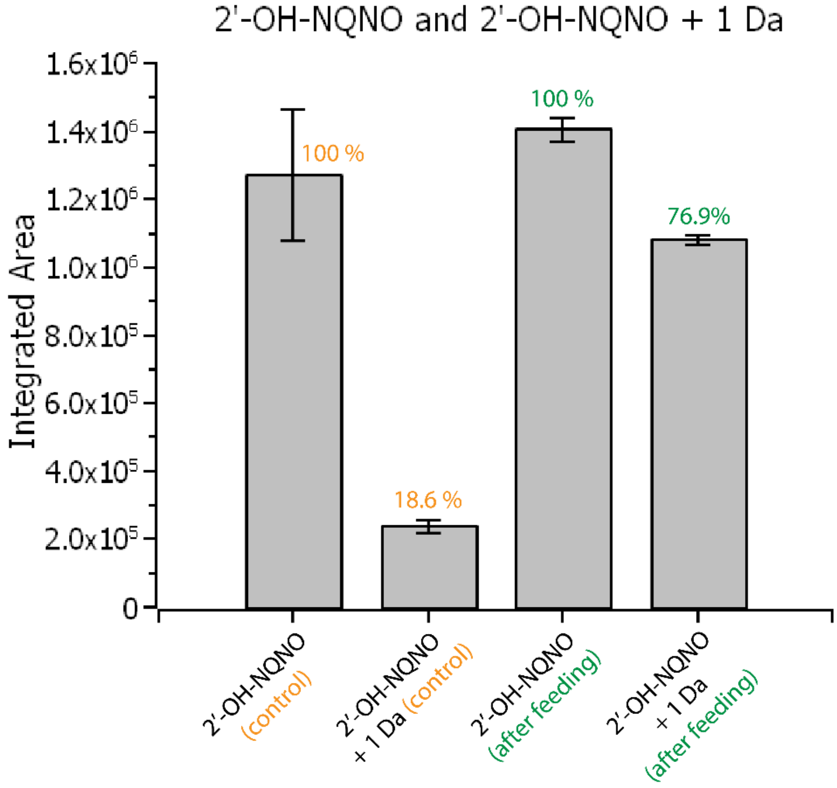

Figure S12. Levels of **a)** 2'-OH-NQ and 2'-OH-NQ + 1 Da; **b)** 2'-OH-NQNO and 2'-OH-NQNO + 1 Da production in *P. aeruginosa* PAO1 in control (orange labels) and after feeding 100 µM deuterated β-hydroxydecanoic acid (**3d**) to cultures (green labels). The error bars represent the population standard deviation.

6. Supplementary References

[1] Fulmer *et. al.* Organometallics. 2010, 29, 2176-2179.

[2] Zheng *et. al.* Bioorg. Med. Chem. 2018, 26, 5934-5943.

[3] Vleeschouwer *et. al.* Chem. Eur. J. 2014, 20, 7766-7775.

[4] Hodgkinson *et. al*. Tetrahedron Lett. 2011, 52 (26), 3291-3294.

[5] Szamosvari *et al.* Chem. Commun., 2020, 56, 6328-6331.
