# Supplementary material for: Biosynthetic flexibility of Pseudomonas aeruginosa leads to hydroxylated 2-alkylquinolones with proinflammatory host response
Source: Commun Chem. 2023 Jul 3;6:138. doi: 10.1038/s42004-023-00937-y (PMC10318067; doi:10.1038/s42004-023-00937-y)
Supplement: Supplementary file 2 — Description of Additional Supplementary Files [file 42004_2023_937_MOESM2_ESM.docx]

Description of Additional Supplementary Files

**File name:** Supplementary Data 1

**Description:** NMR spectra data

**File name:** Supplementary Data 2

**Description:** Raw data for Figure 5a (Growth curves of *S*. *aureus* USA300 in the presence of 50 µM of various quinolones and DMSO as a control)

**File name:** Supplementary Data 3

**Description:** Raw data for Figure 5b (IL-8 release from differentiated Caco-2 cells after 24 h of treatment with medium (control), 10 nM LPS, and 100 nM of various quinolones)
