# Supplementary material for: Biosynthetic flexibility of Pseudomonas aeruginosa leads to hydroxylated 2-alkylquinolones with proinflammatory host response
Source: Commun Chem. 2023 Jul 3;6:138. doi: 10.1038/s42004-023-00937-y (PMC10318067; doi:10.1038/s42004-023-00937-y)
Supplement: Supplementary file 3 — Supplementary Data 1 [file 42004_2023_937_MOESM3_ESM.pdf]

NMR spectra

Biosynthetic flexibility of *Pseudomonas aeruginosa* leads to hydroxylated 2-Alkylquinolones with proinflammatory host response

Viktoriia Savchenko[a,b], Dávid Szamosvári[a], Yifan Bao[b,c], Marc Pignitter[c] and Thomas Böttcher*[a]

[a] Faculty of Chemistry, Institute for Biological Chemistry & Centre for Microbiology and Environmental Systems Science, Department of Microbiology and Ecosystems Science, University of Vienna, Josef-Holaubek-Platz 2 (UZA II), 1090 Vienna, Austria

[b] Vienna Doctoral School in Chemistry (DoSChem), University of Vienna, Währinger Str. 42, 1090 Vienna, Austria

[c] Faculty of Chemistry, Institute of Physiological Chemistry, University of Vienna, Josef-Holaubek-Platz 2, 1090 Vienna, Austria

E-mail: thomas.boettcher@univie.ac.at

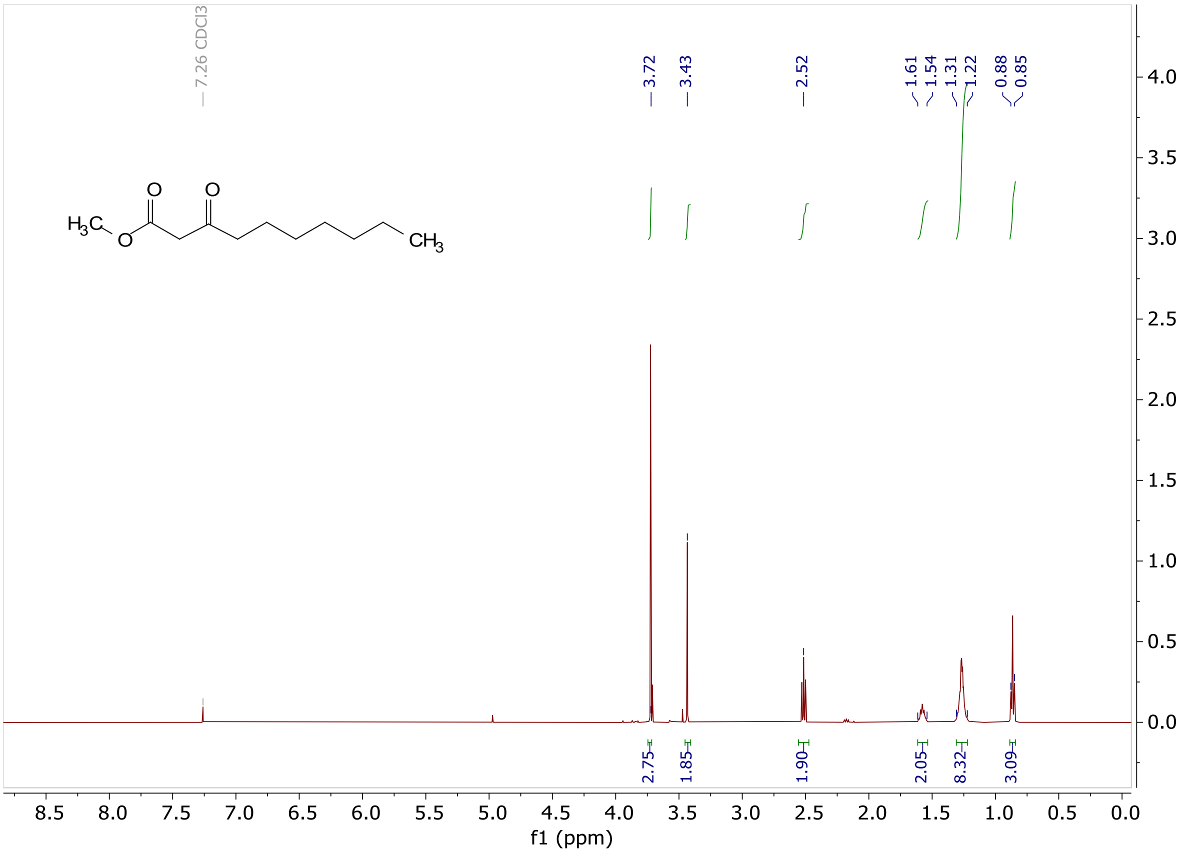

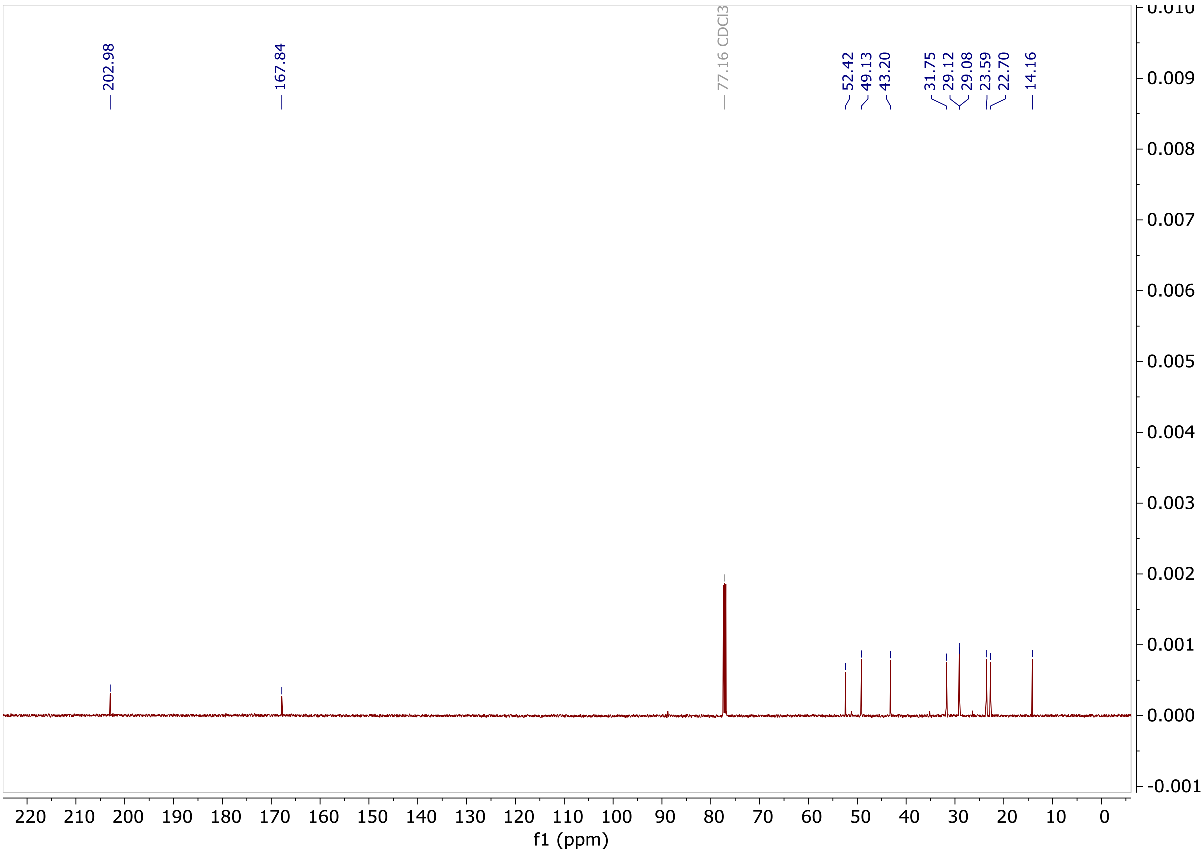

Methyl 3-oxodecanoate (**a**) ^{1}H-NMR (above) and ^{13}C-NMR (below) in $CDCl_3$

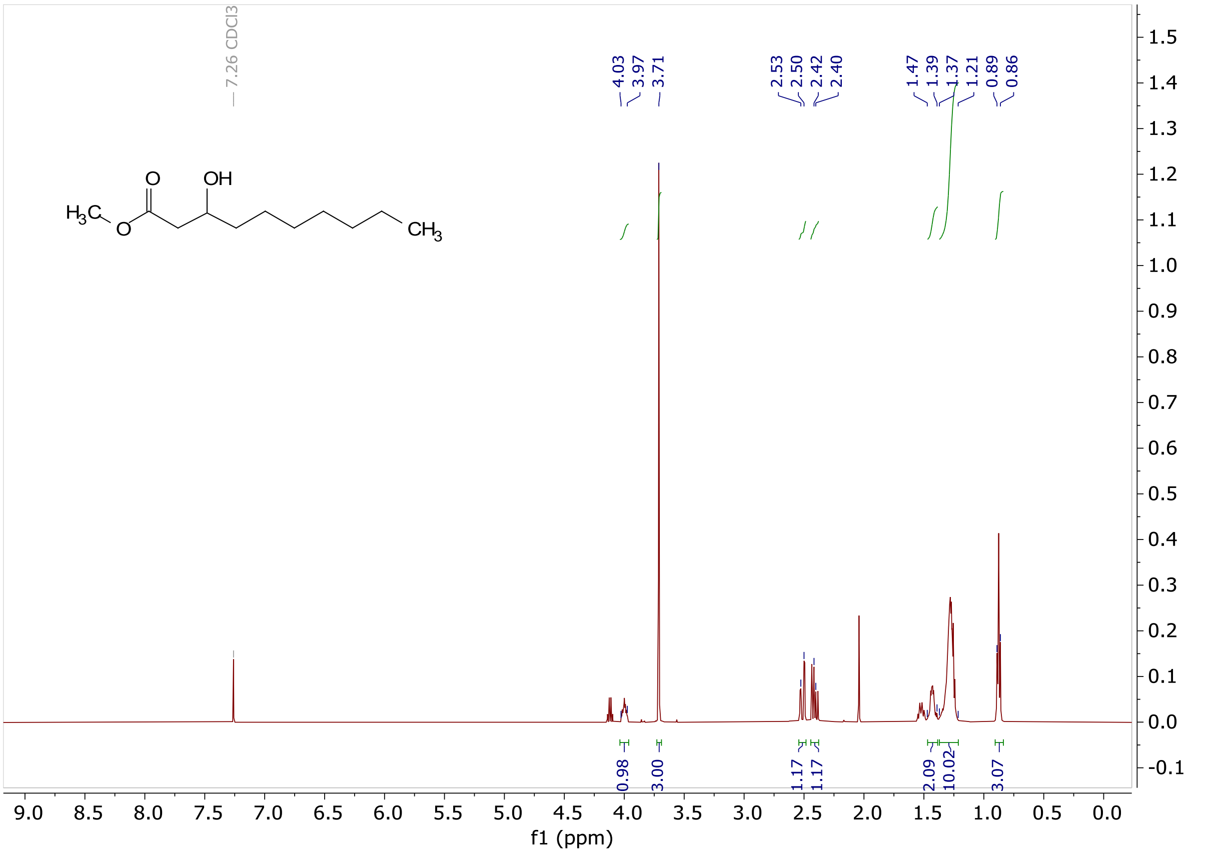

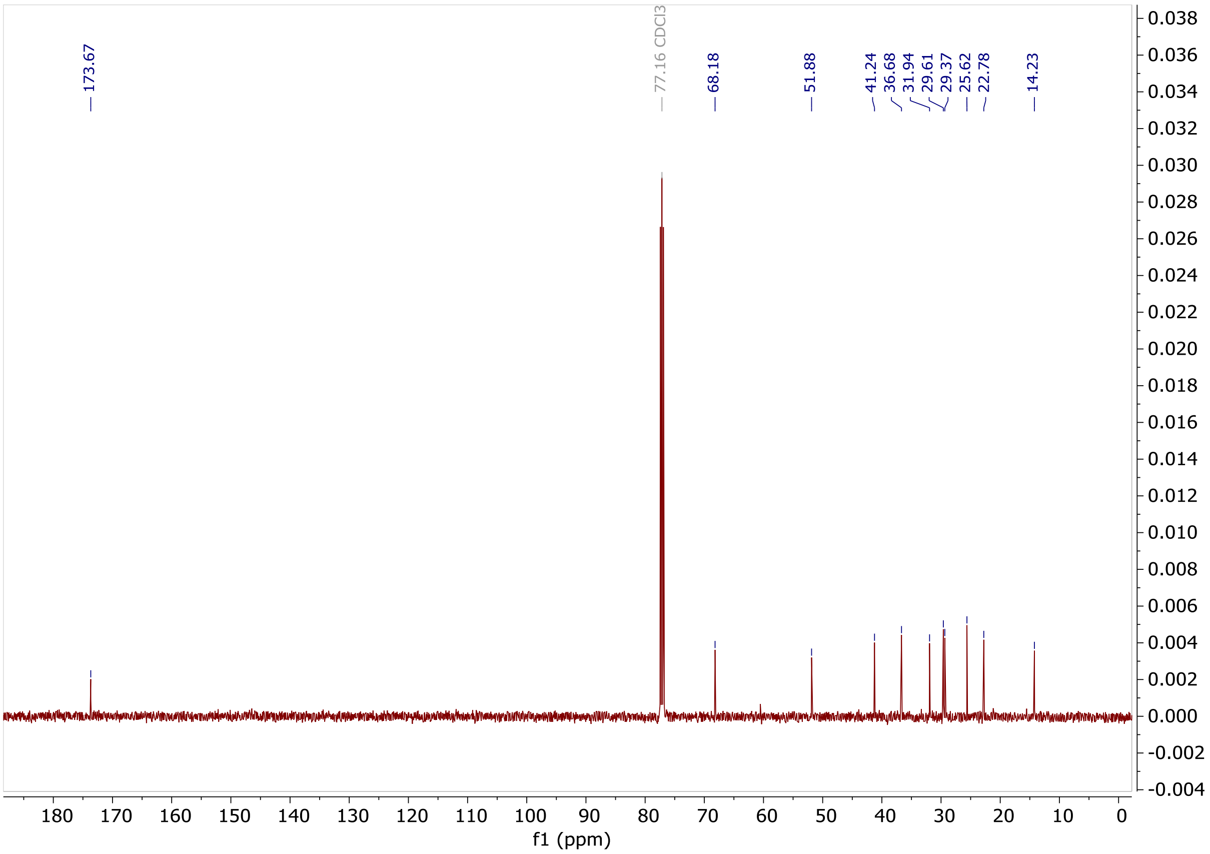

Methyl 3-hydroxydecanoate (**b**) ^{1}H-NMR (above) and ^{13}C-NMR (below) in $CDCl_3$

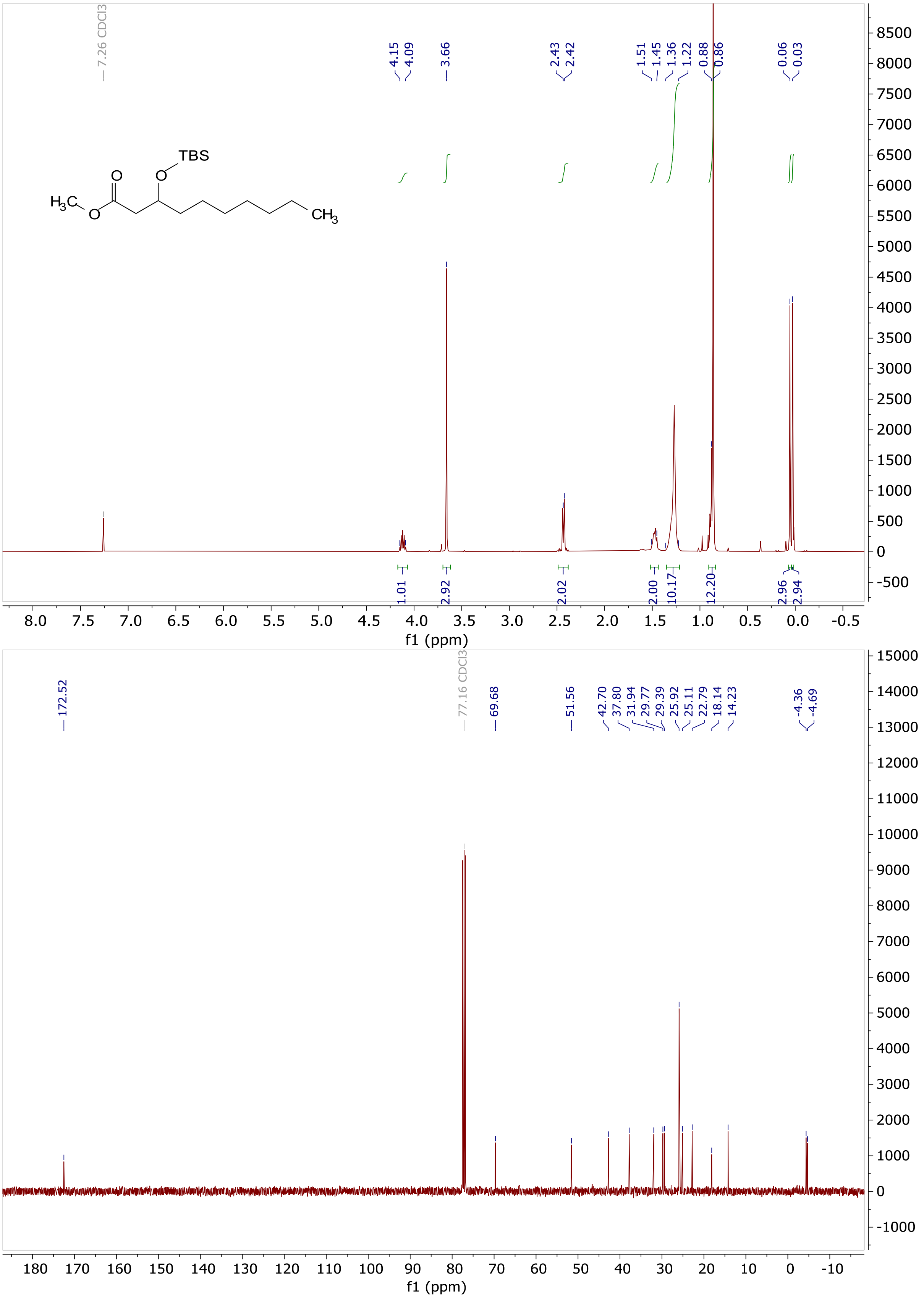

Methyl 3-((*tert*-butyldimethylsilyl)oxy)decanoate (**1a**) ^{1}H-NMR (above) and ^{13}C-NMR (below) in $CDCl_3$

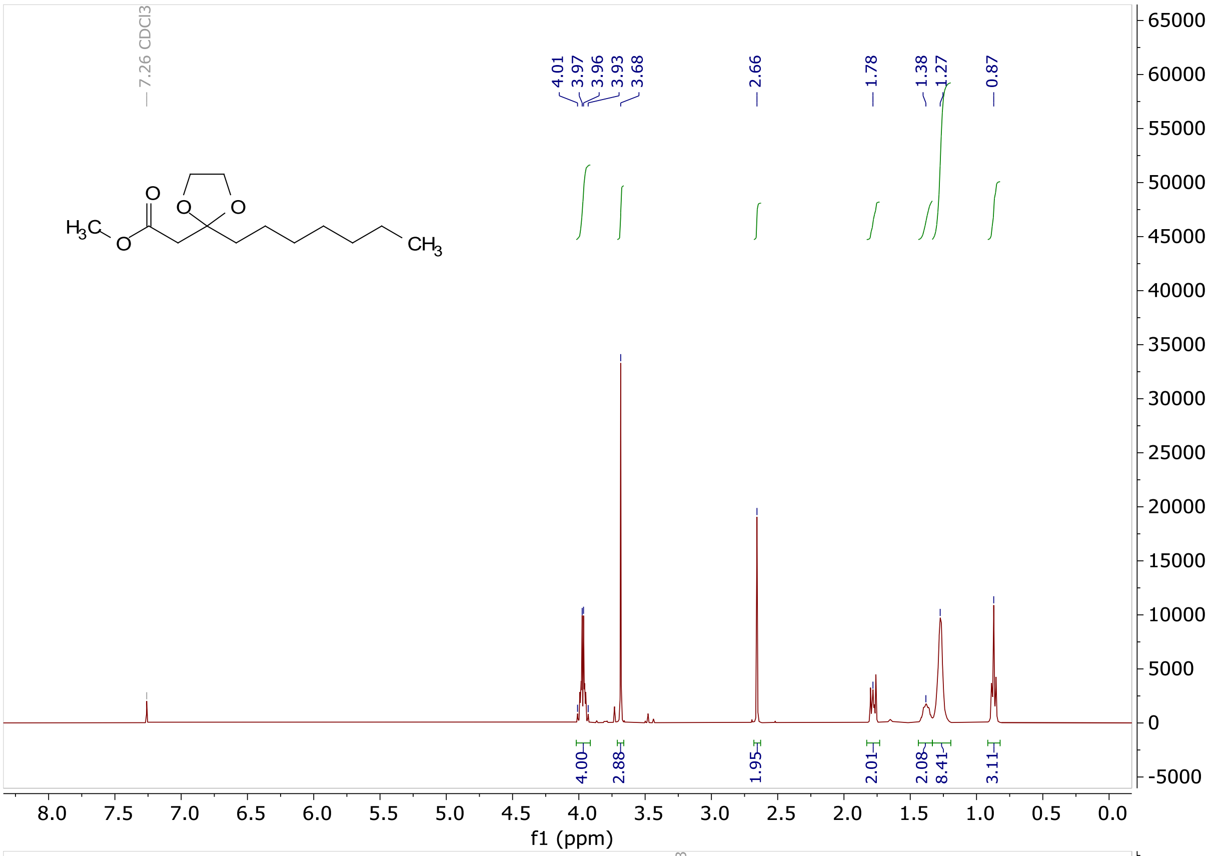

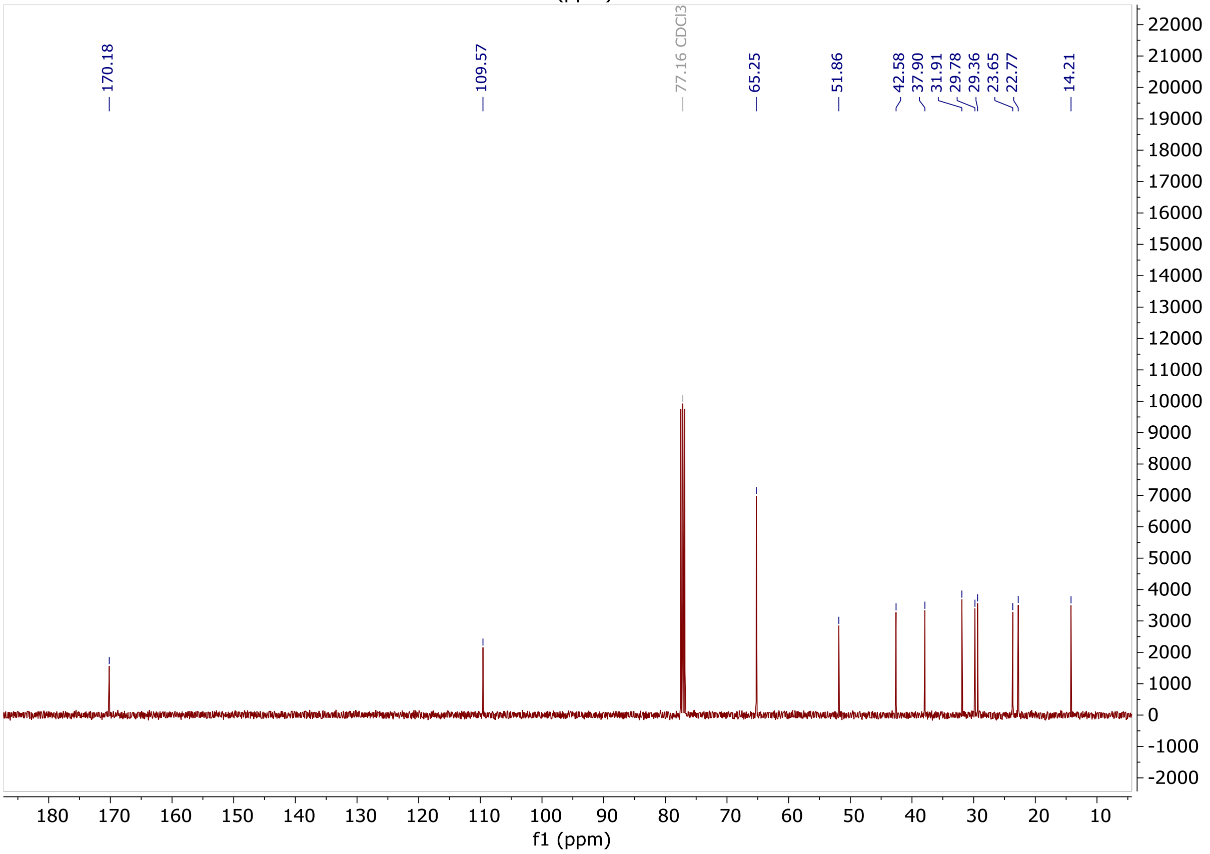

Methyl 3-(1,3-dioxolane)decanoate (**2a**) ^{1}H-NMR (above) and ^{13}C-NMR (below) in $CDCl_3$

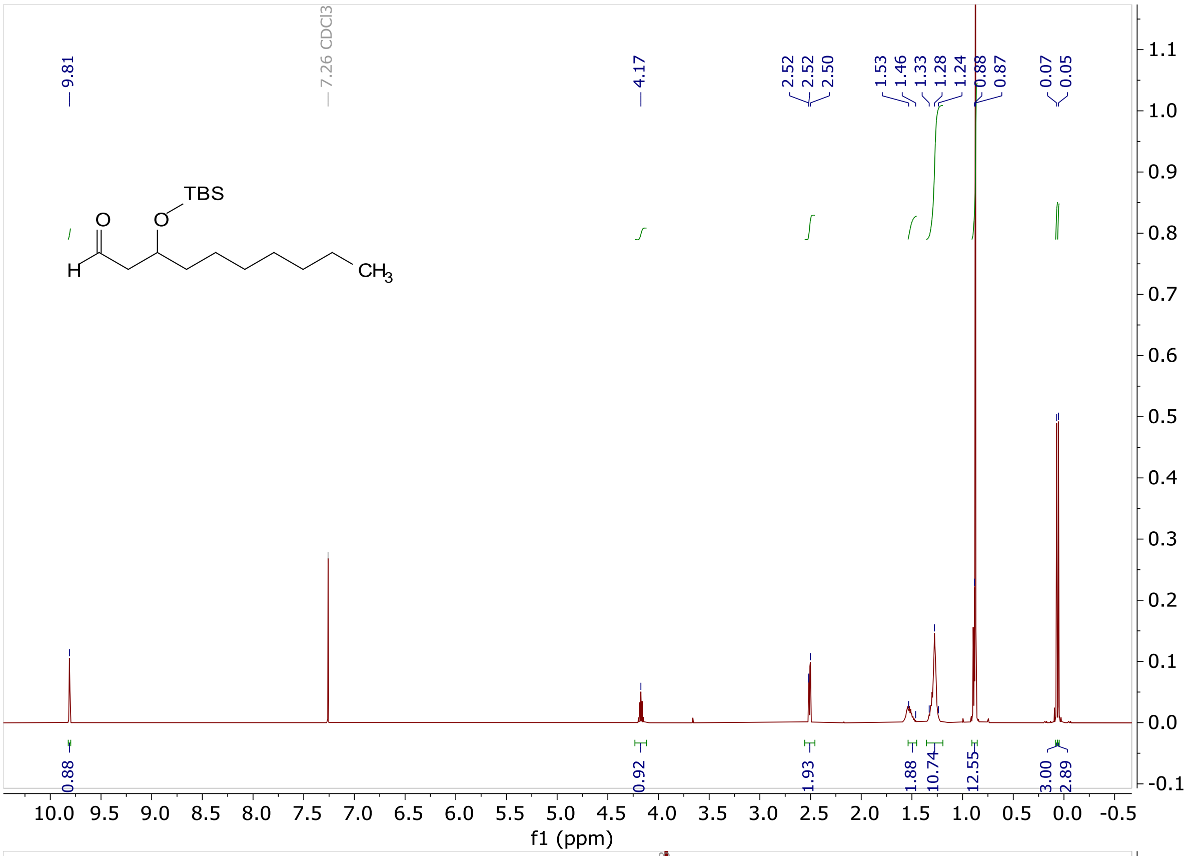

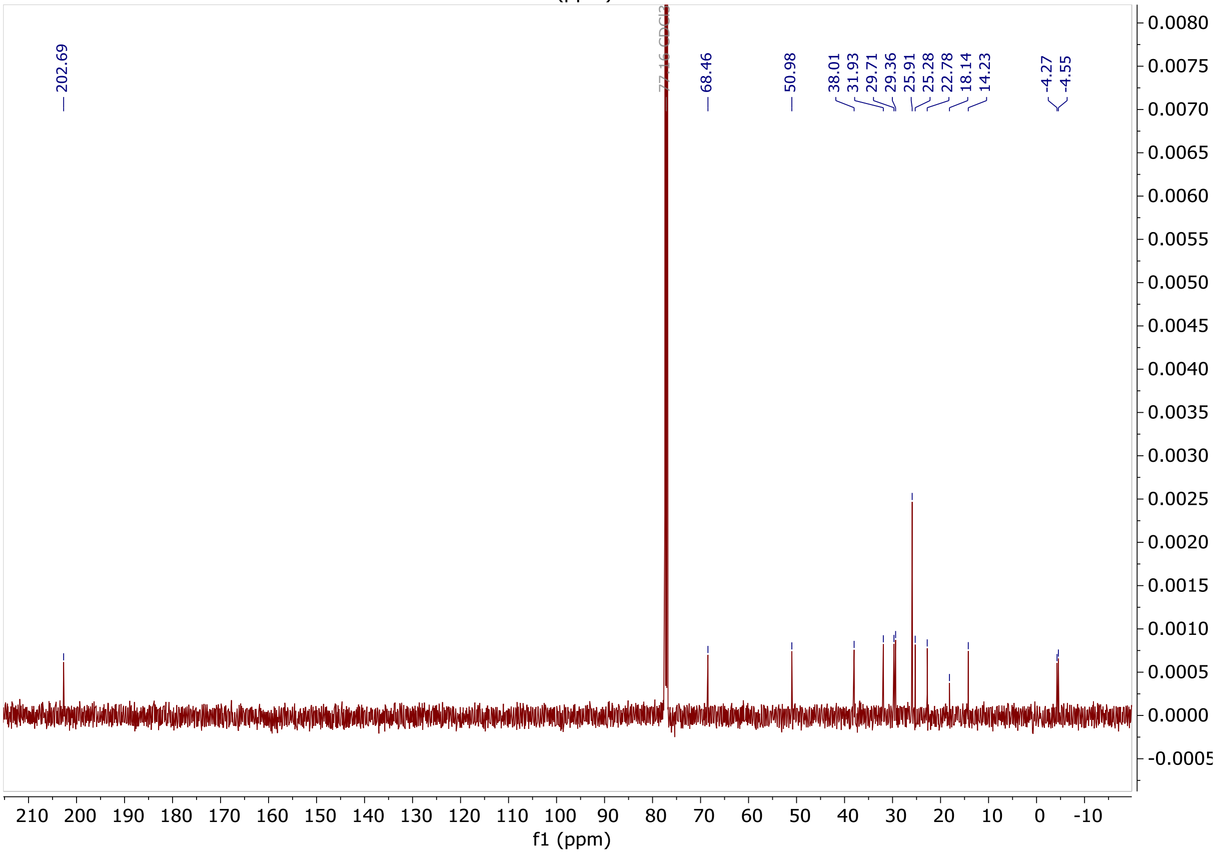

3-((*tert*-butyldimethylsilyl)oxy)decanal (**1b**) ^{1}H-NMR (above) and ^{13}C-NMR (below) in $CDCl_3$

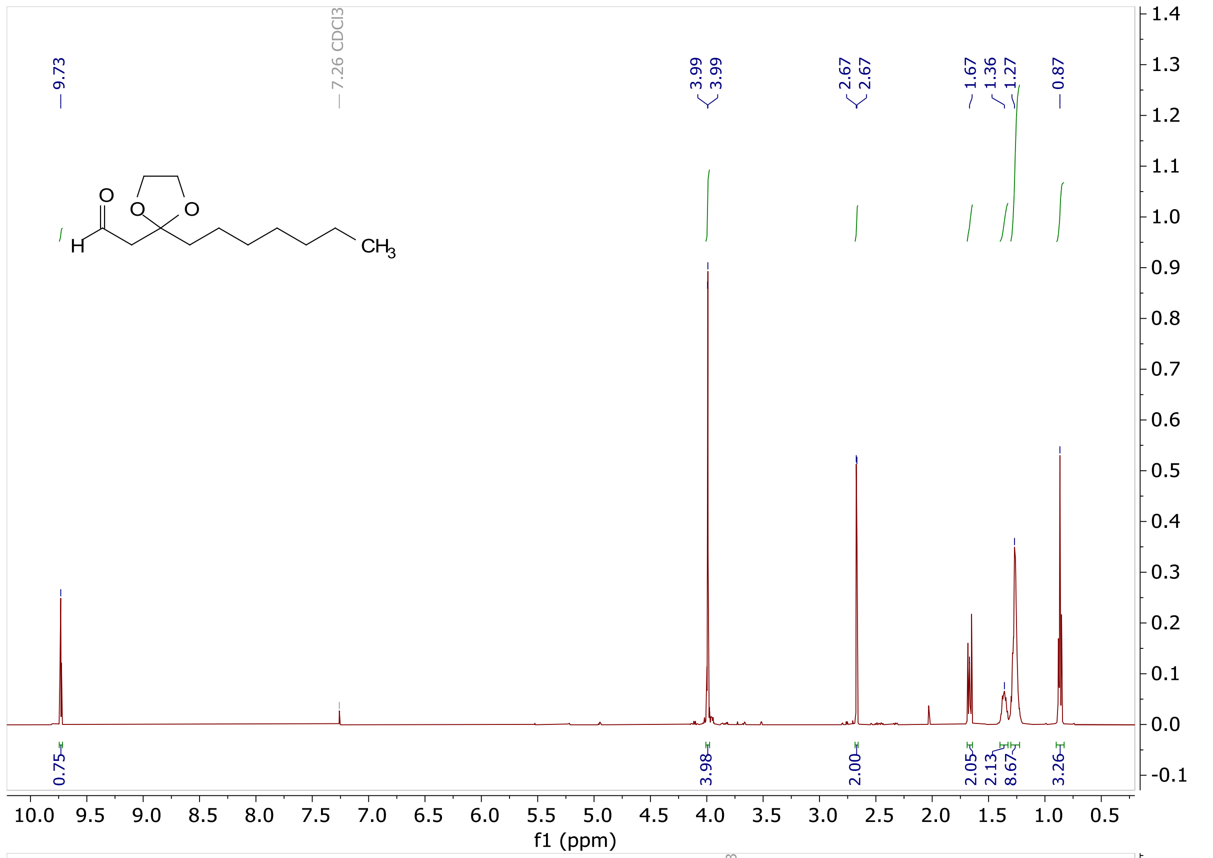

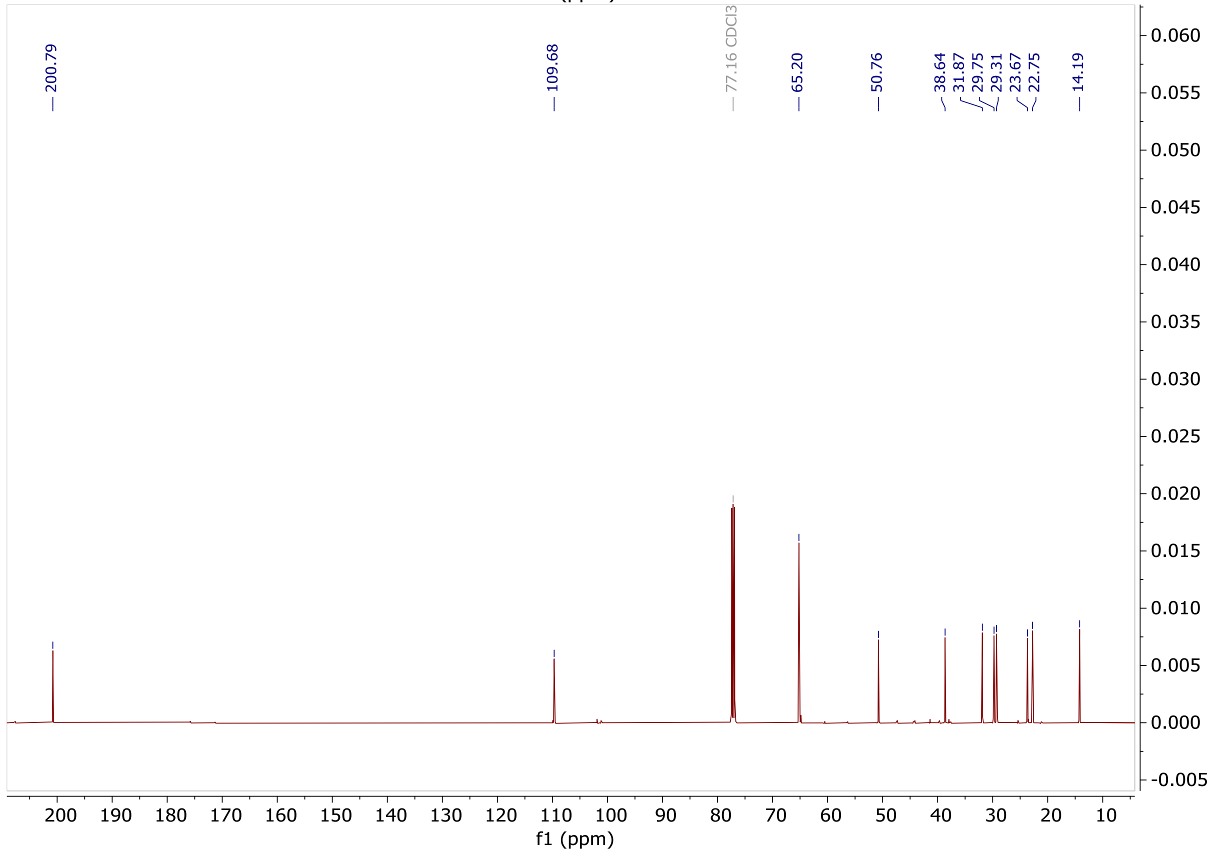

3-(1,3-dioxolane)decanal (**2b**) ^{1}H-NMR (above) and ^{13}C-NMR (below) in $CDCl_3$

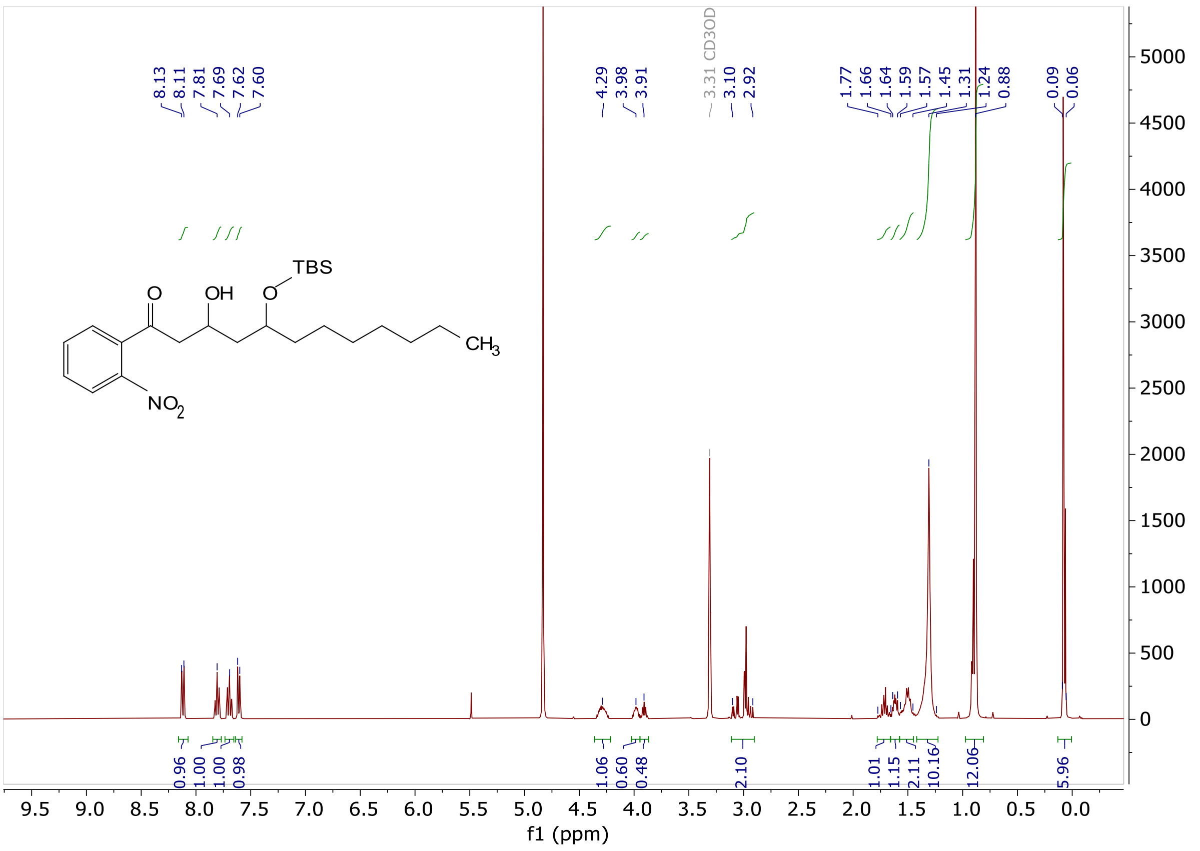

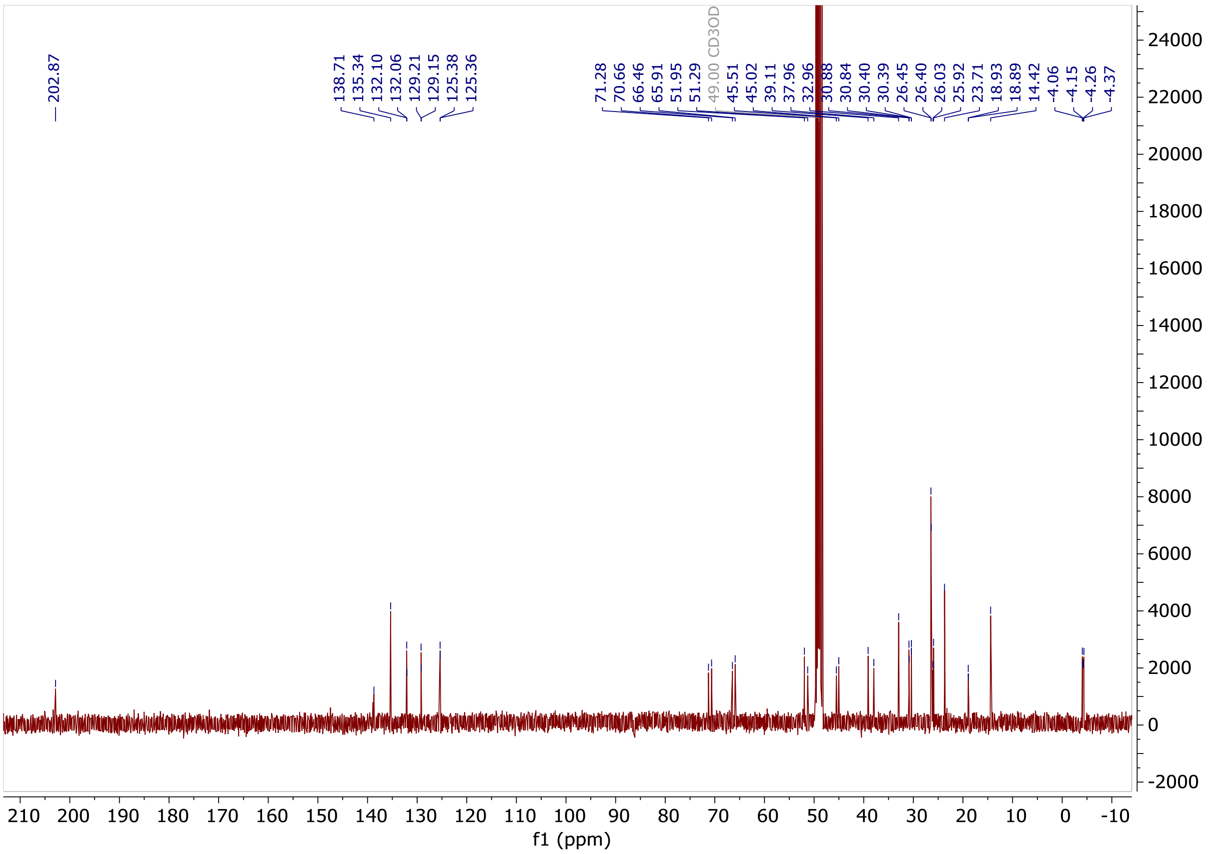

5-((*tert*-butyldimethylsilyl)oxy)-3-hydroxy-1-(2'-nitrophenyl)dodecan-1-one (**1c**) ^{1}H-NMR (above) and ^{13}C-NMR (below) in MeOH-d_4

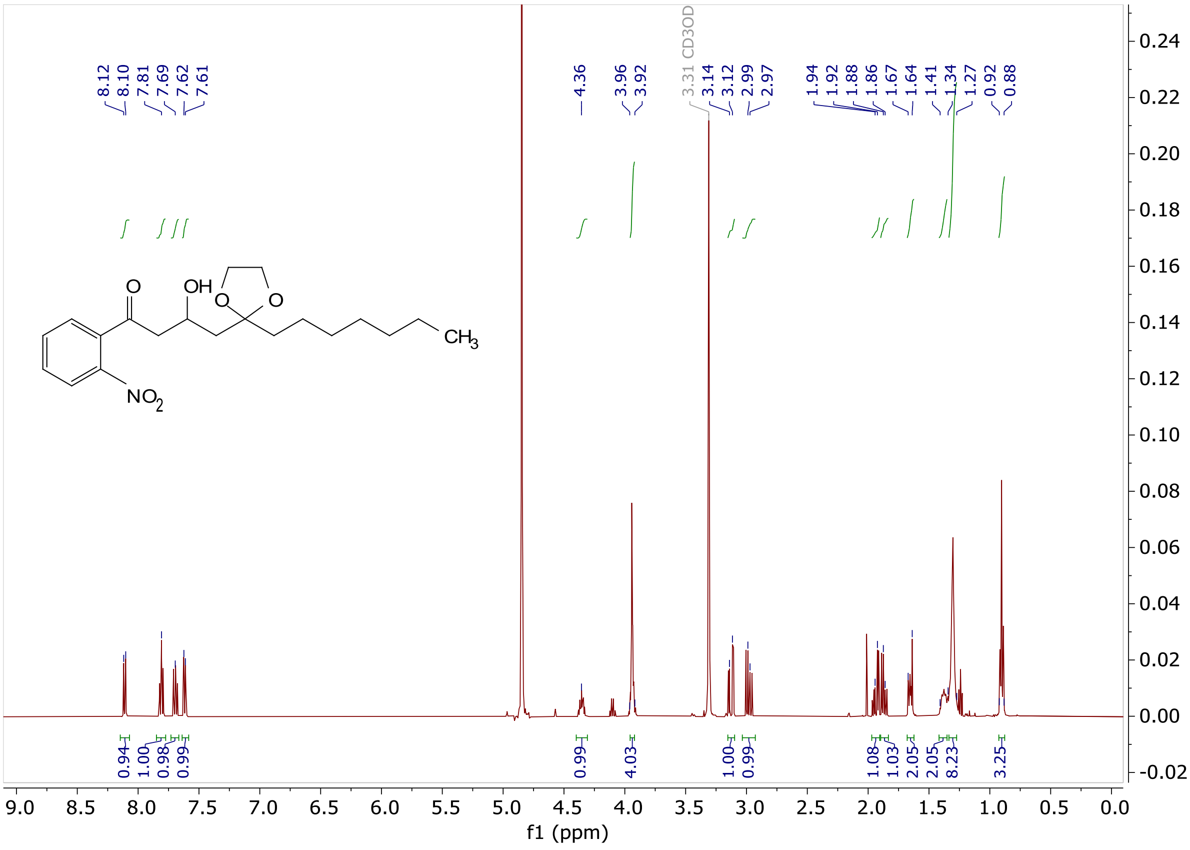

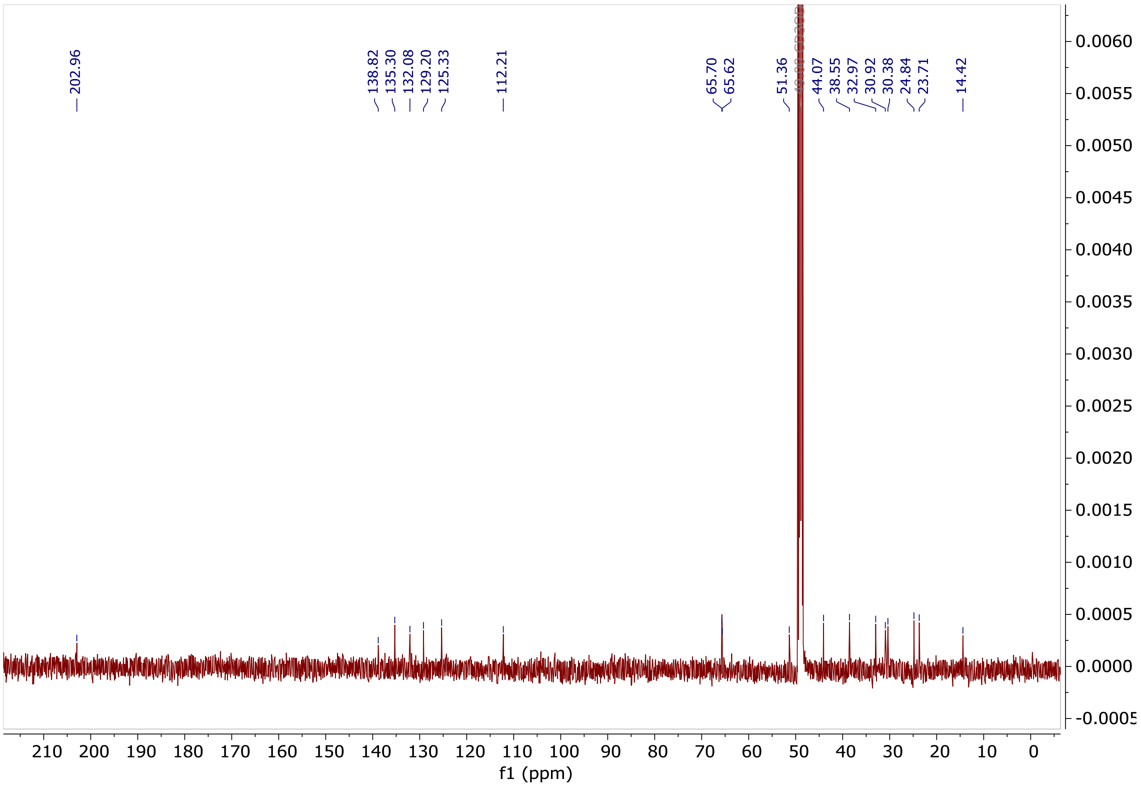

5-(1,3-dioxolane)-3-hydroxy-1-(2'-nitrophenyl)dodecan-1-one (**2c**) ^{1}H-NMR (above) and ^{13}C-NMR (below) in MeOH-d_4

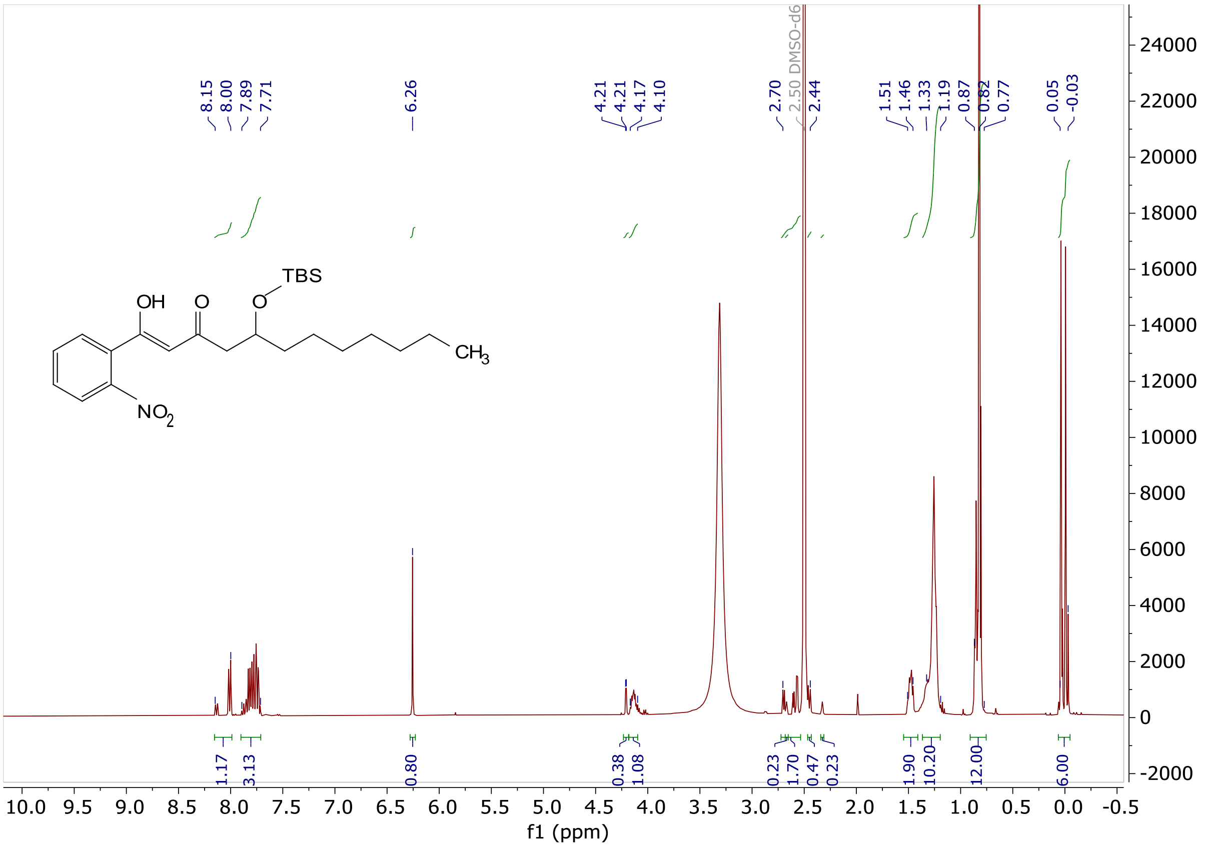

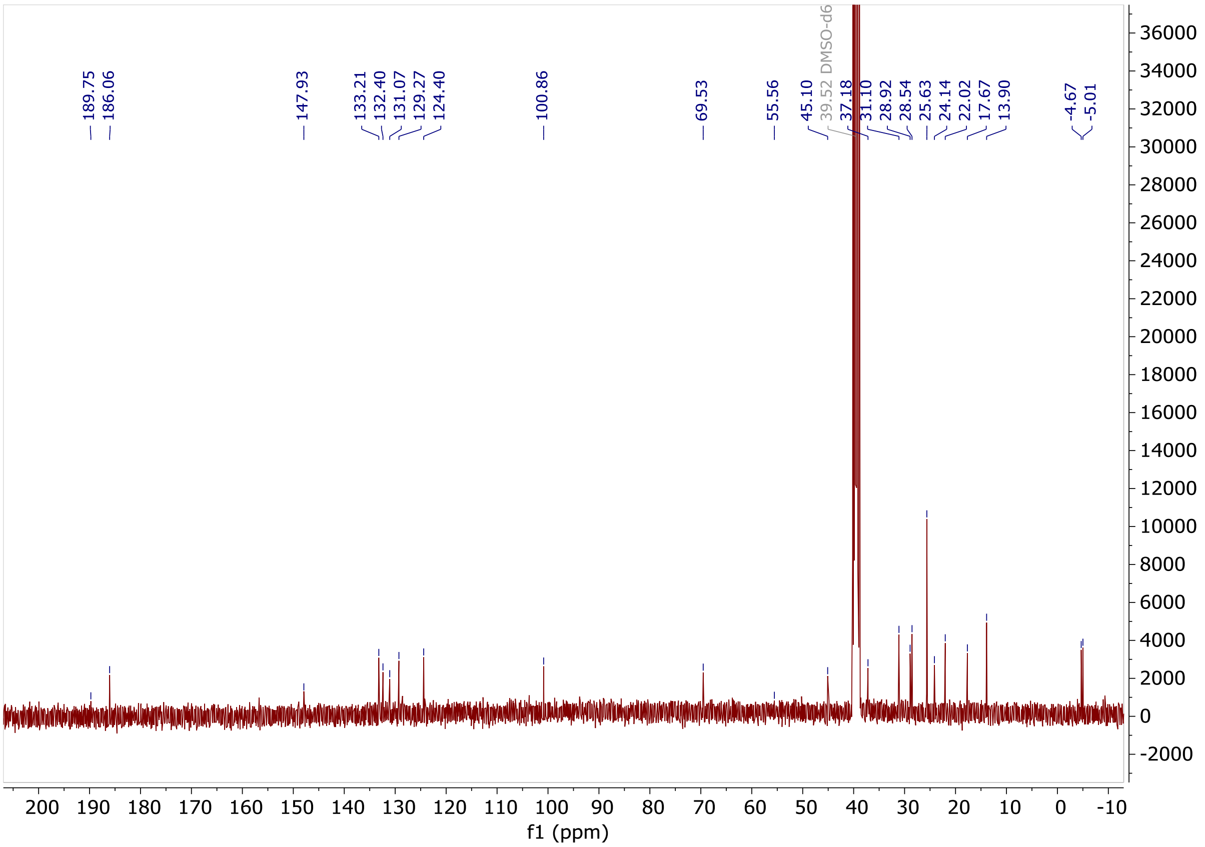

5-((*tert*-butyldimethylsilyl)oxy)-3-oxo-1-(2'-nitrophenyl)dodecan-1-one (**1d**) in ^{1}H-NMR (above) and ^{13}C-NMR (below) in DMSO-d_6

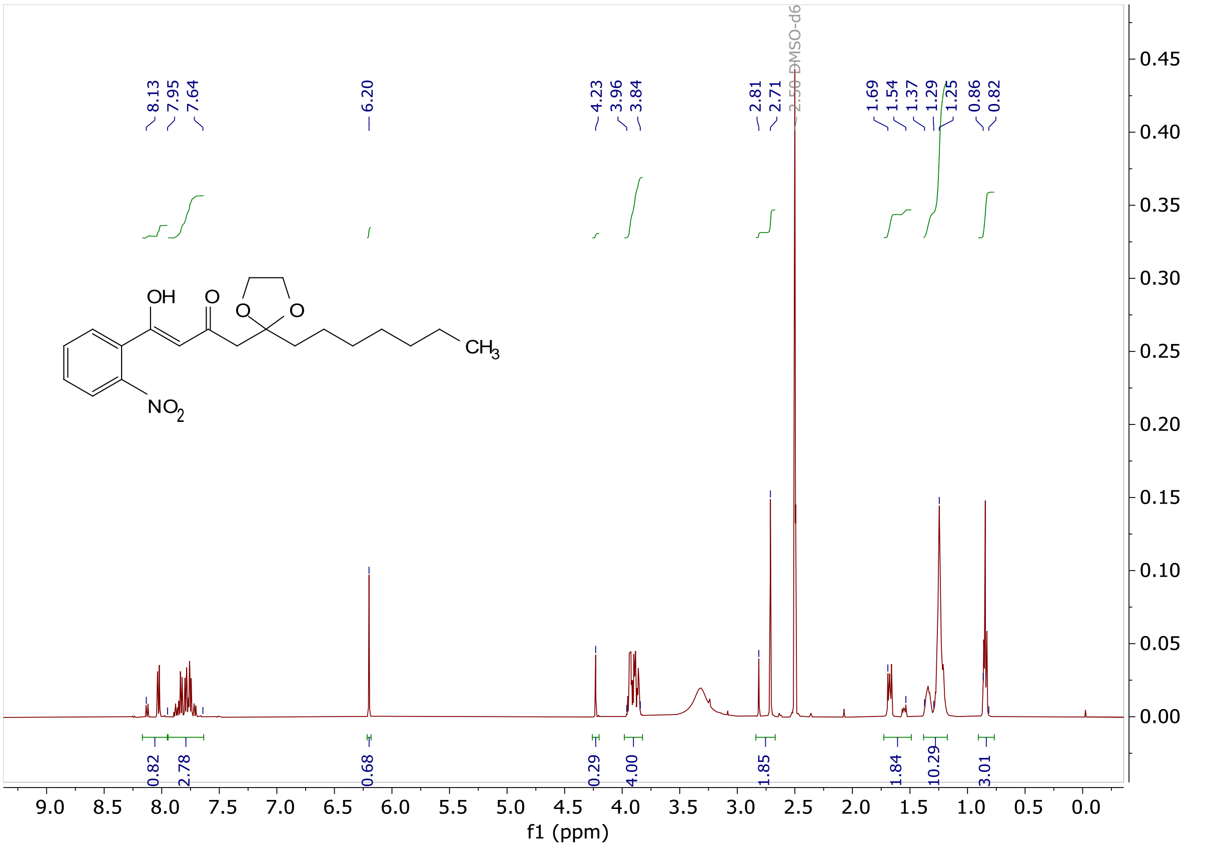

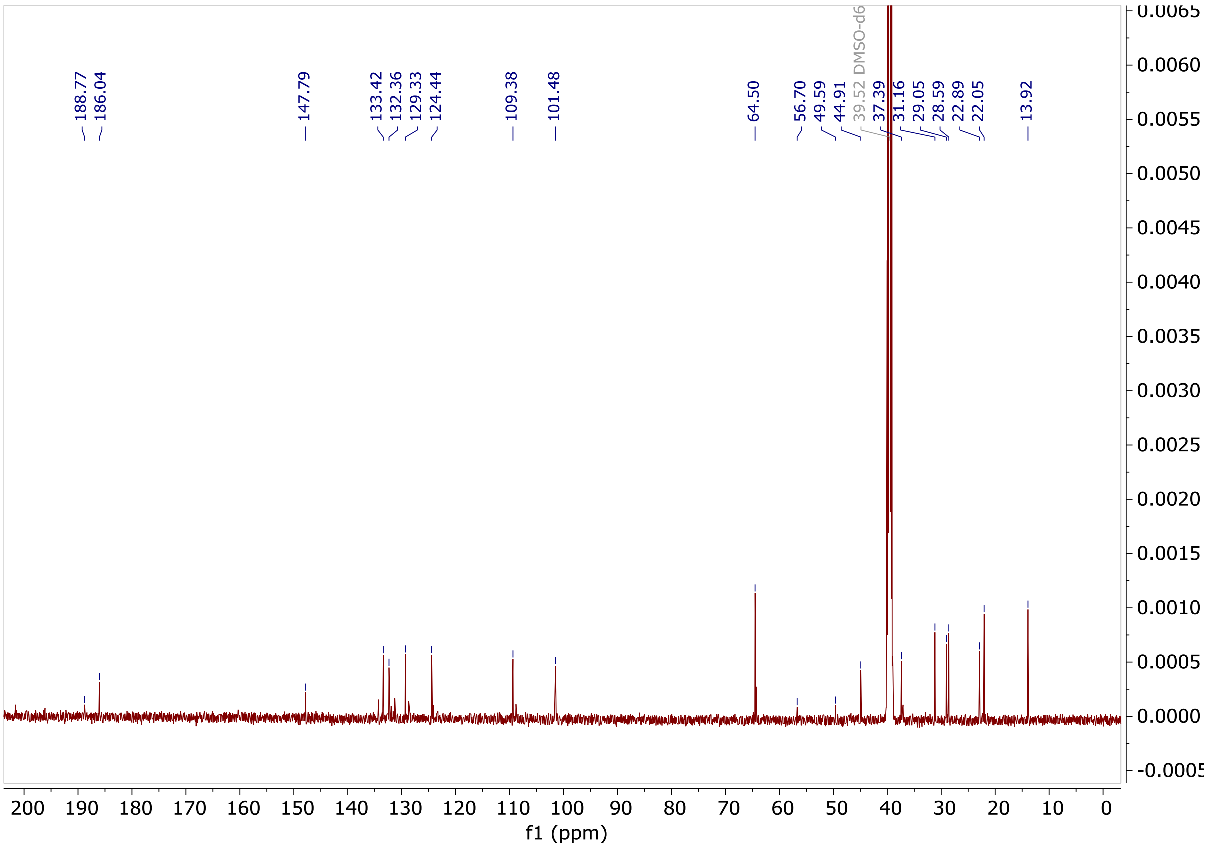

5-(1,3-dioxolane)-3-oxo-1-(2’-nitrophenyl)dodecan-1-one (**2d**) in ^{1}H-NMR (above) and ^{13}C-NMR (below) in DMSO-d_6

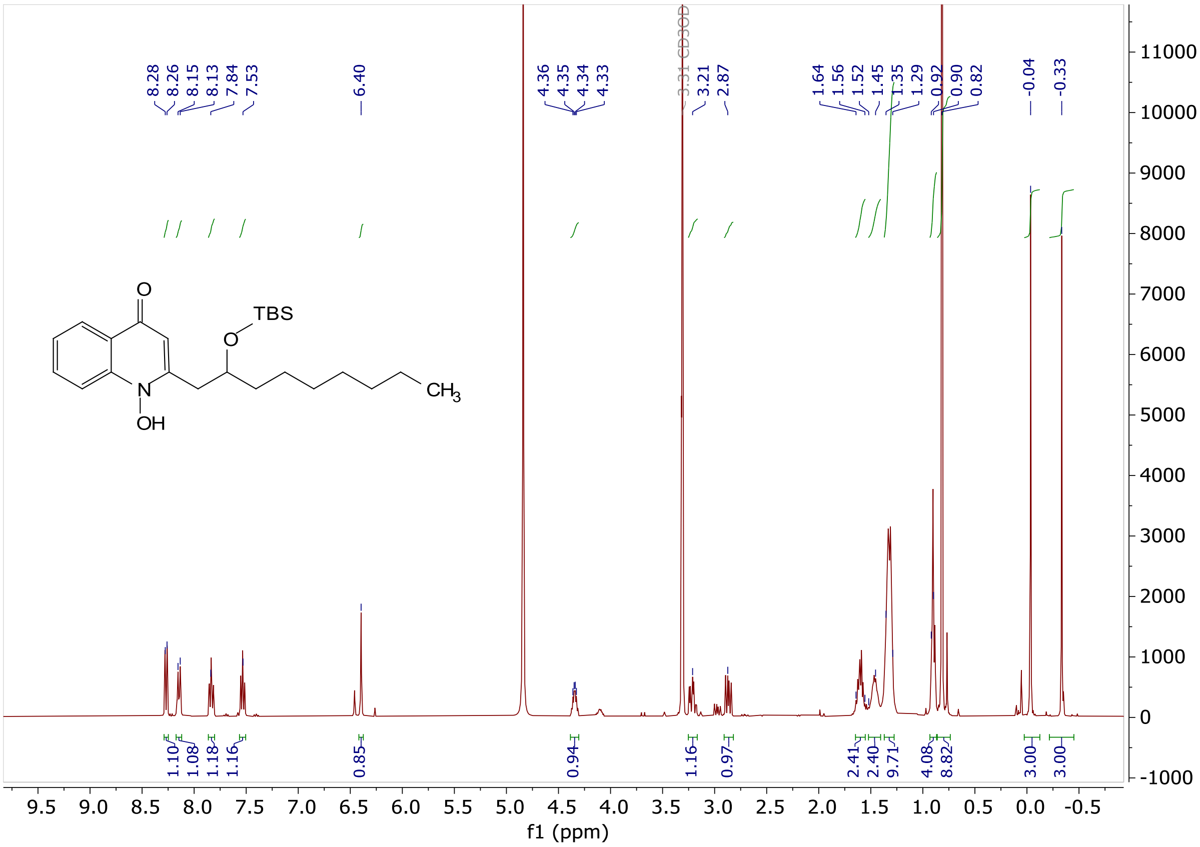

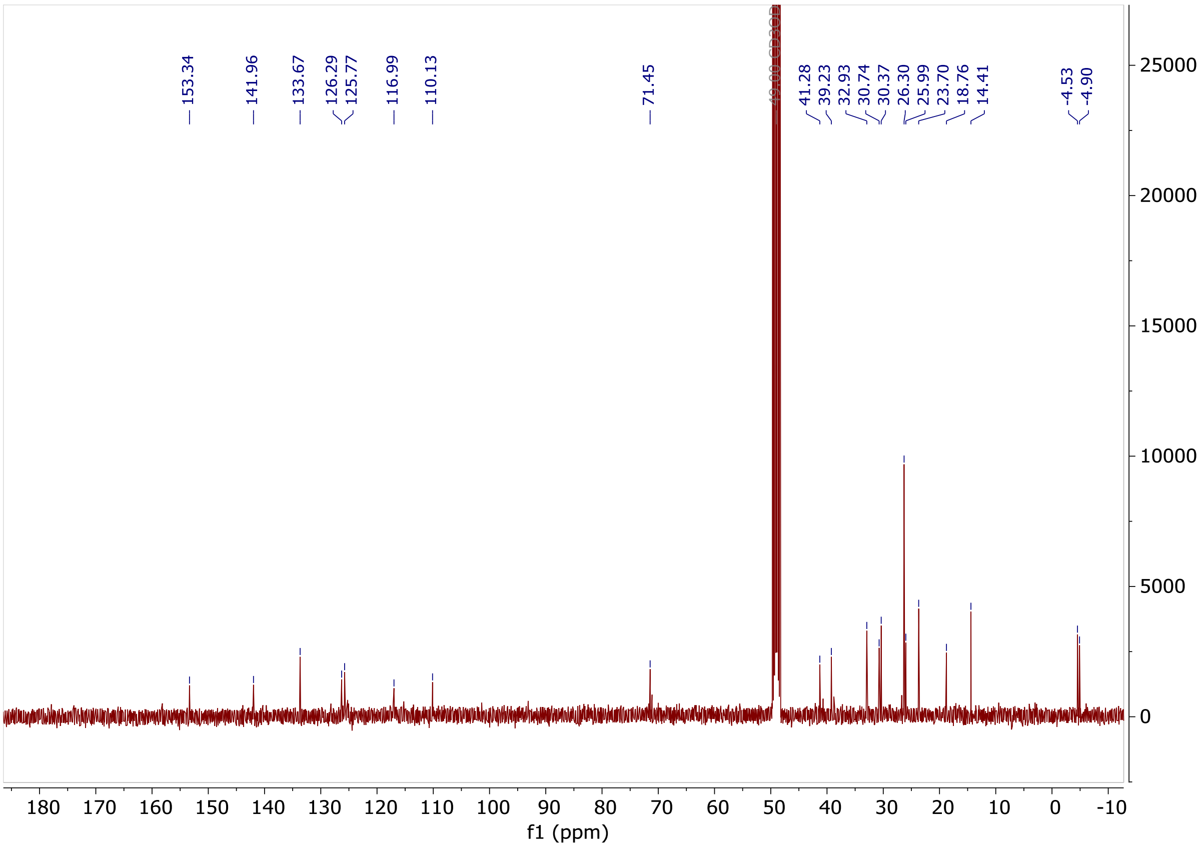

2-(2′-((*tert*-butyldimethylsilyl)oxy)nonyl)-1-hydroxyquinolin-4(1*H*)-one (2′-OTBS-NQNO) (**1f**)
^{1}H-NMR (above) and ^{13}C-NMR (below) in MeOH-d$_4$

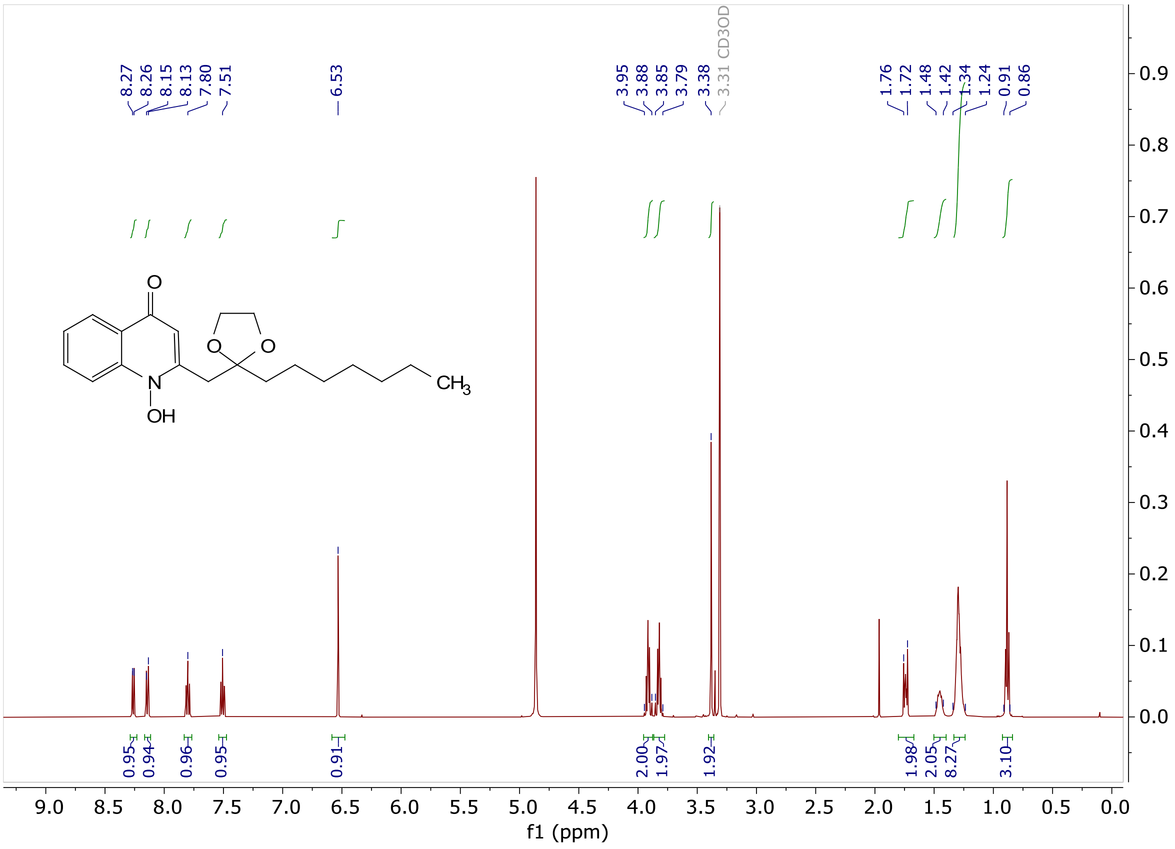

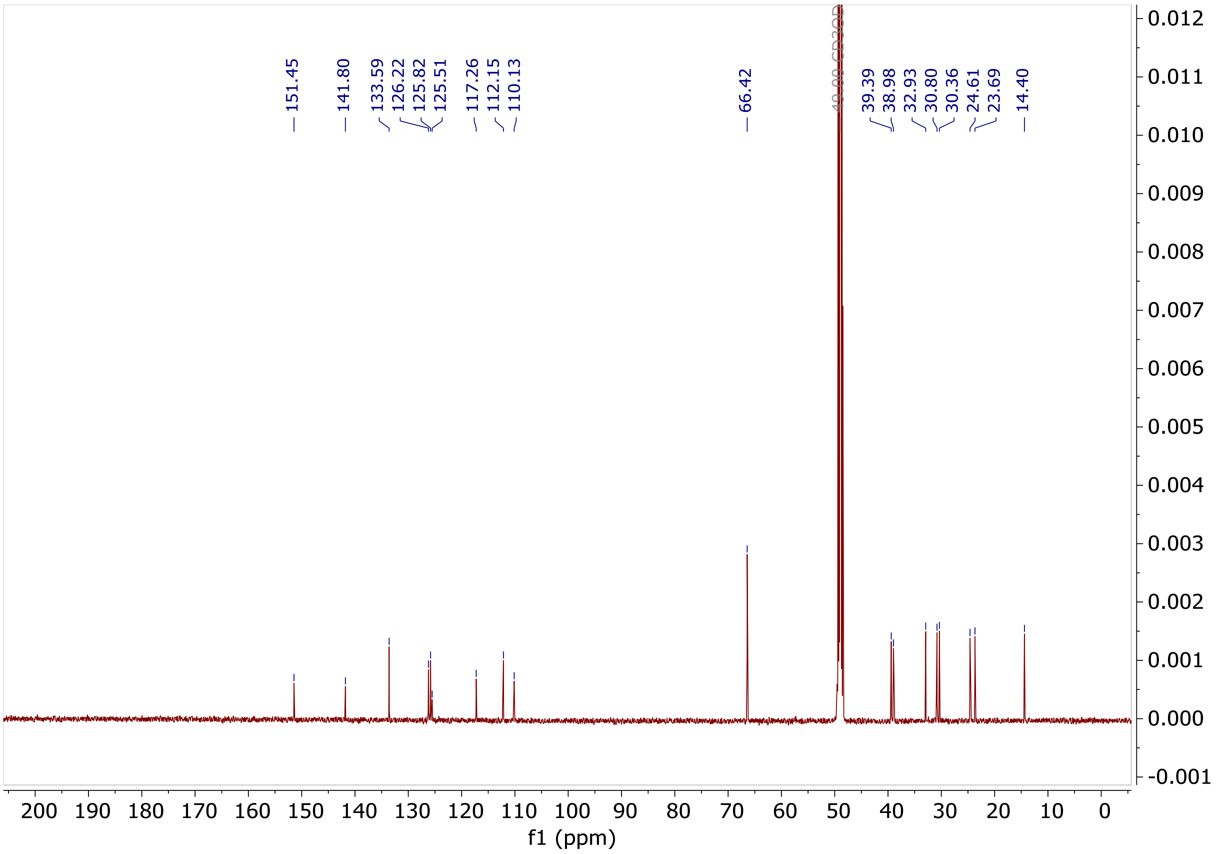

2-(2’-(1,3-dioxolane)-1-hydroxyquinolin-4(1*H*)-one (2’-(1,3-dioxolane)-NQNO) (**2f**) ^{1}H-NMR (above) and ^{13}C-NMR (below) in MeOH-d$_4$

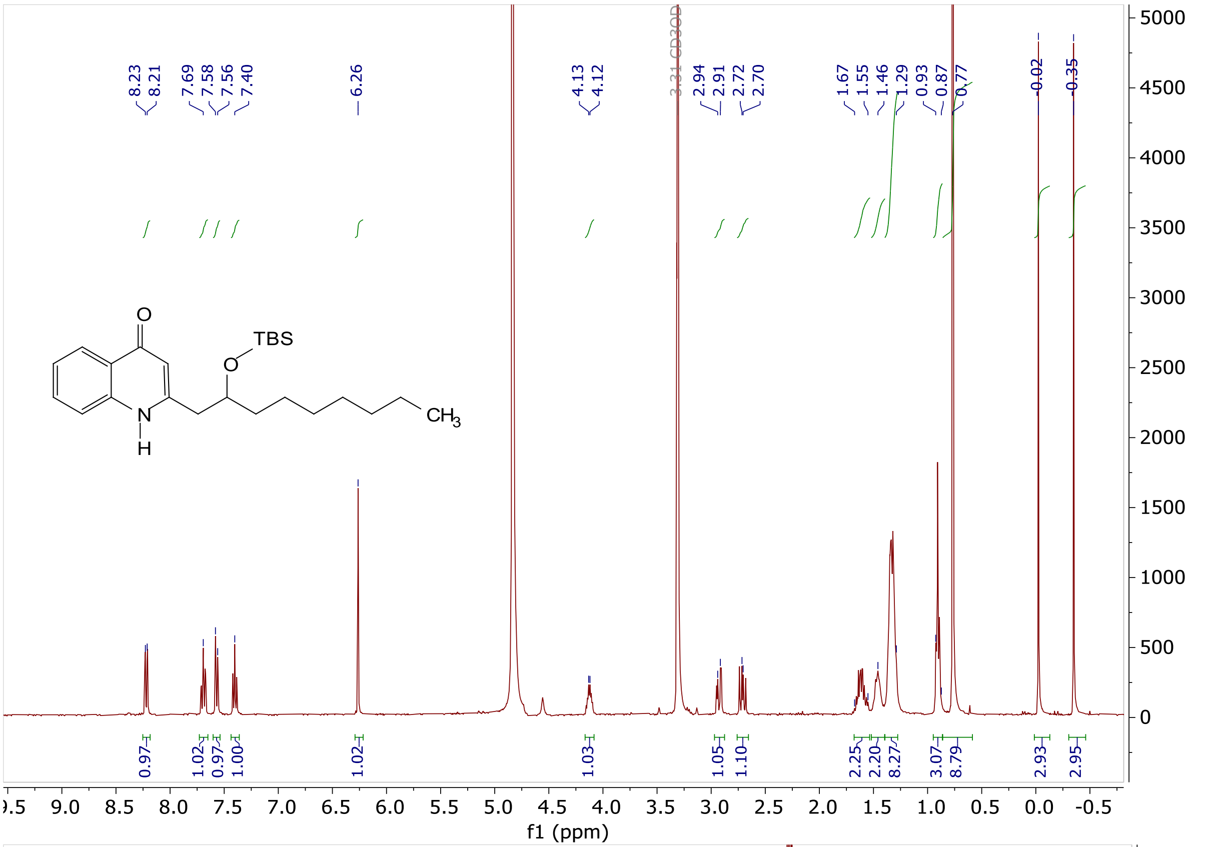

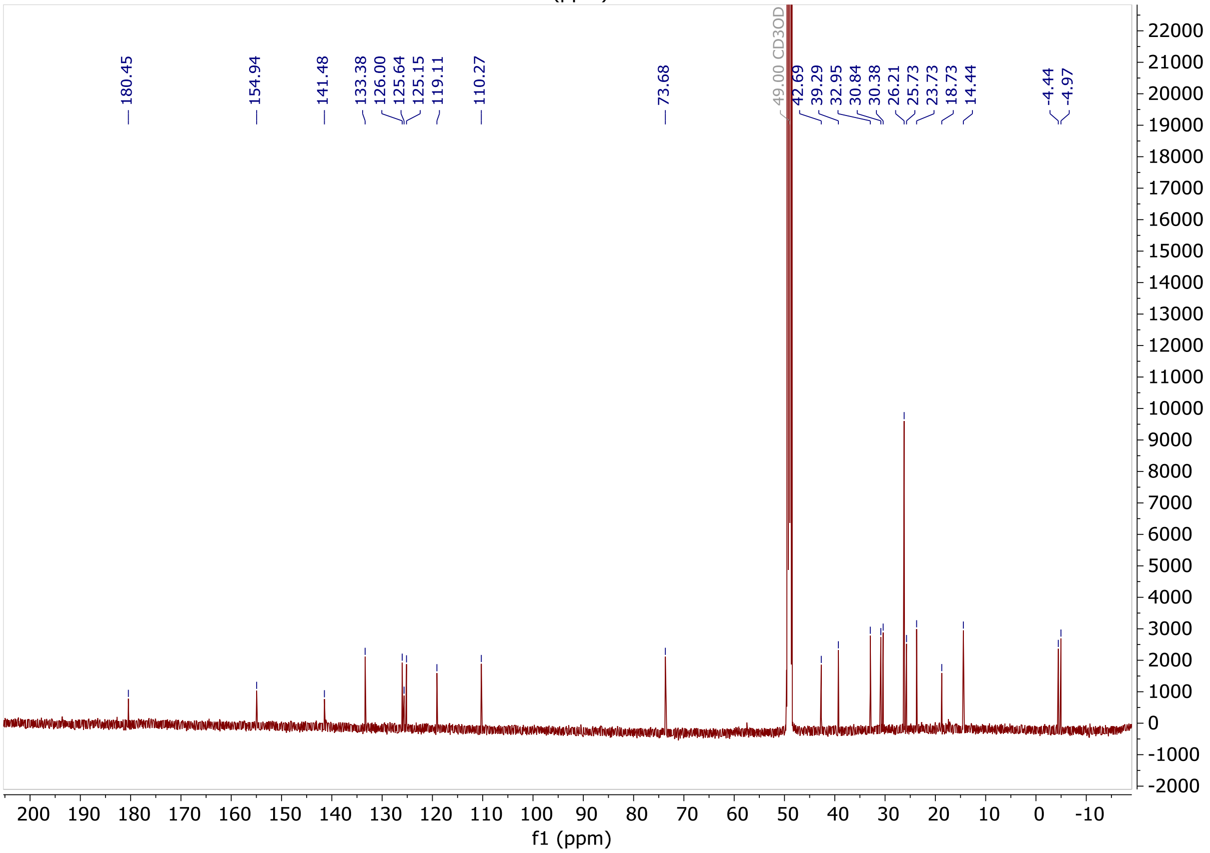

2-(2′-((*tert*-butyldimethylsilyl)oxy)nonyl)quinolin-4(1*H*)-one (2′-OTBS-NQ) (**1e**) ^{1}H-NMR (above) and ^{13}C-NMR (below) in MeOH-d$_4$

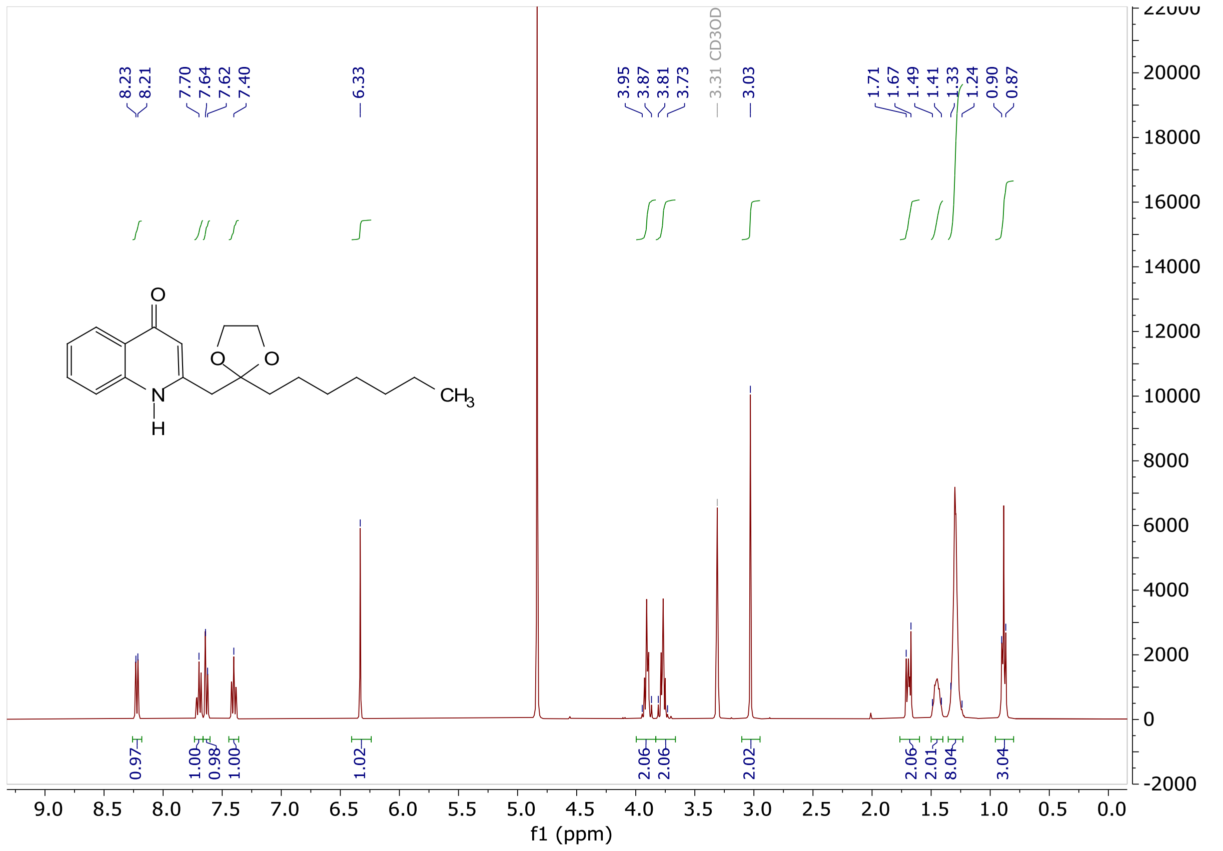

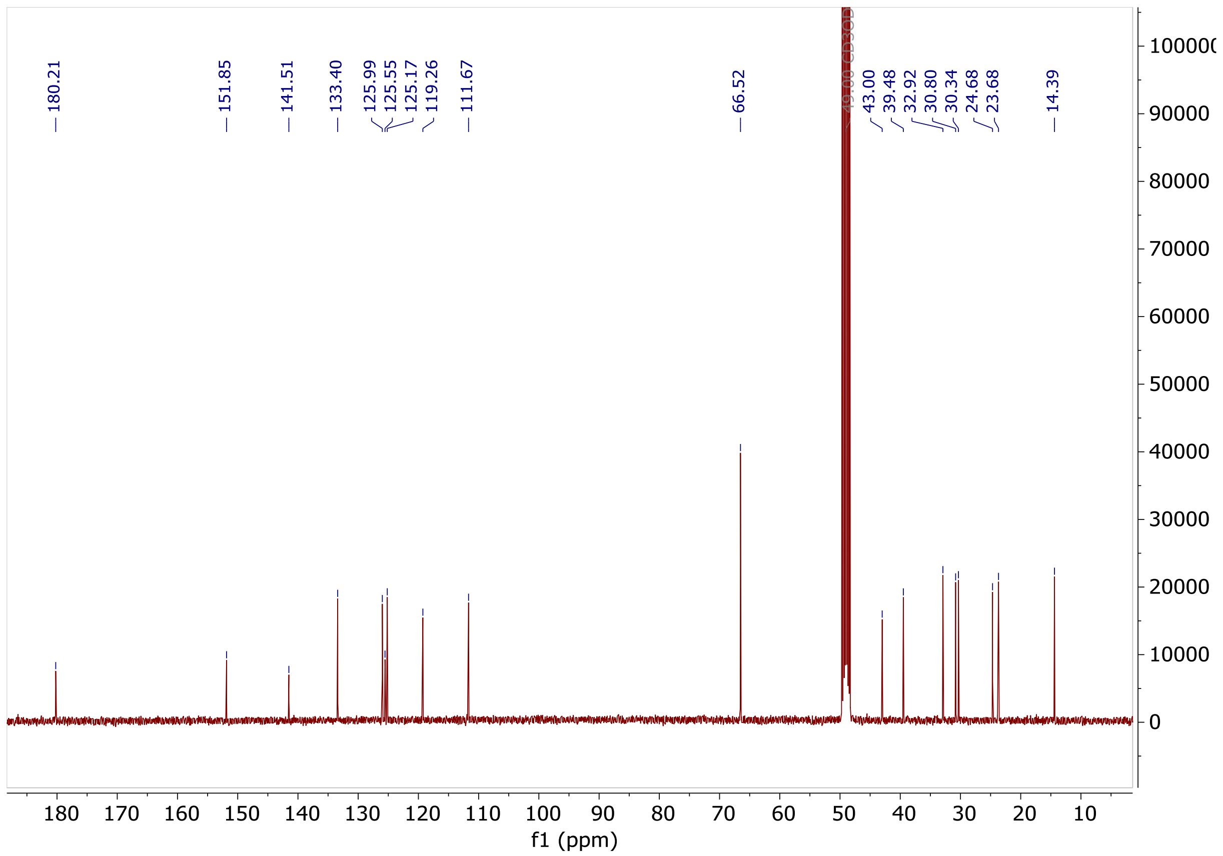

2-(2’-(1,3-dioxolane))quinolin-4(1*H*)-one (2’-(1,3-dioxolane)-NQ) (**2e**) ^{1}H-NMR (above) and ^{13}C-NMR (below) in MeOH-d_4

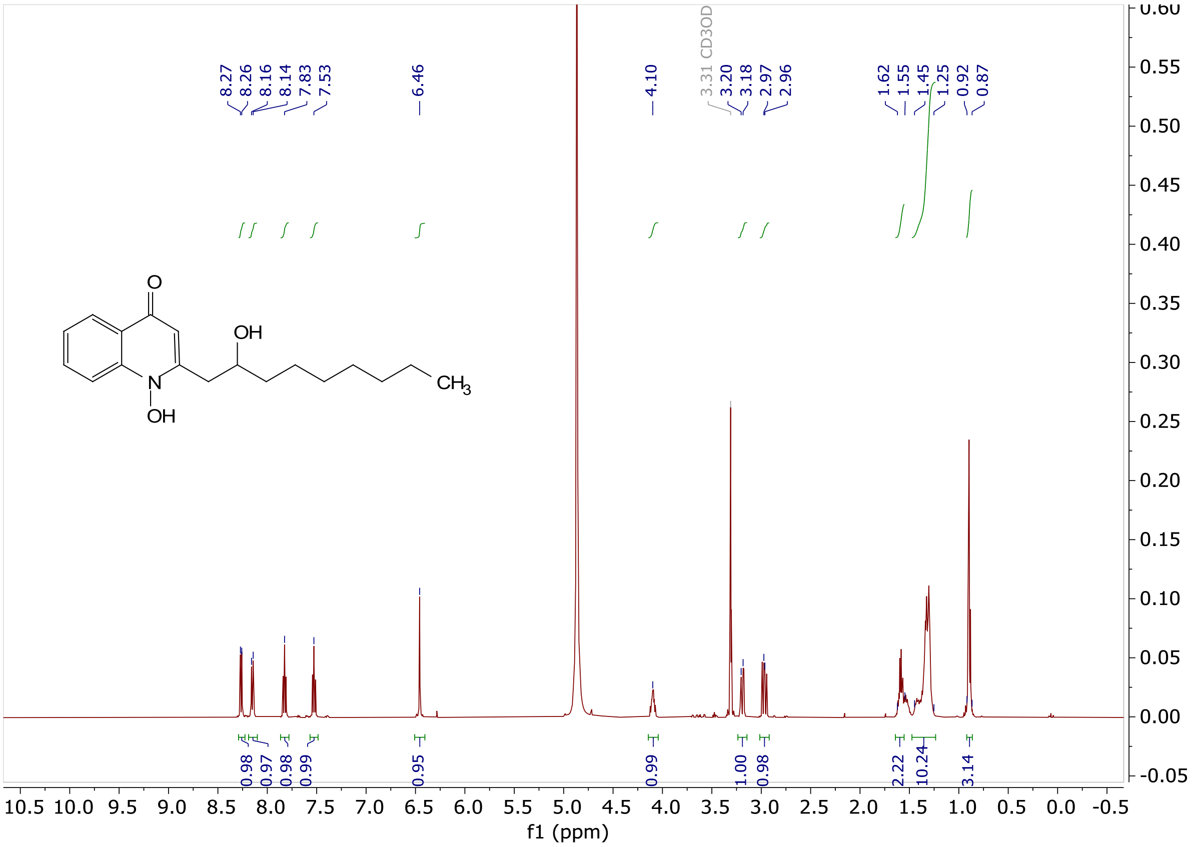

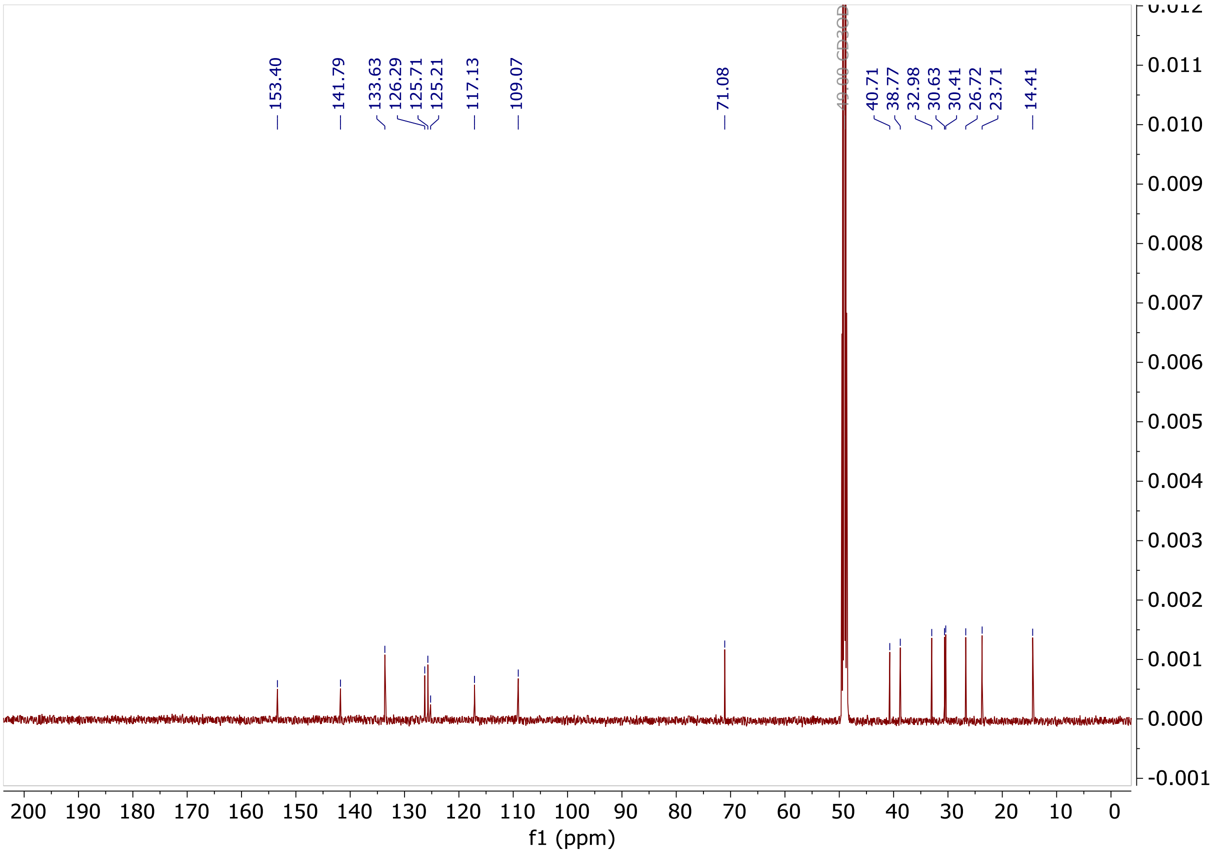

1-hydroxy-2-(2’-hydroxynonyl)quinolin-4(1*H*)-one (2’-OH-NQNO) (**1h**) ^{1}H-NMR (above) and ^{13}C-NMR (below) in MeOH-d_4

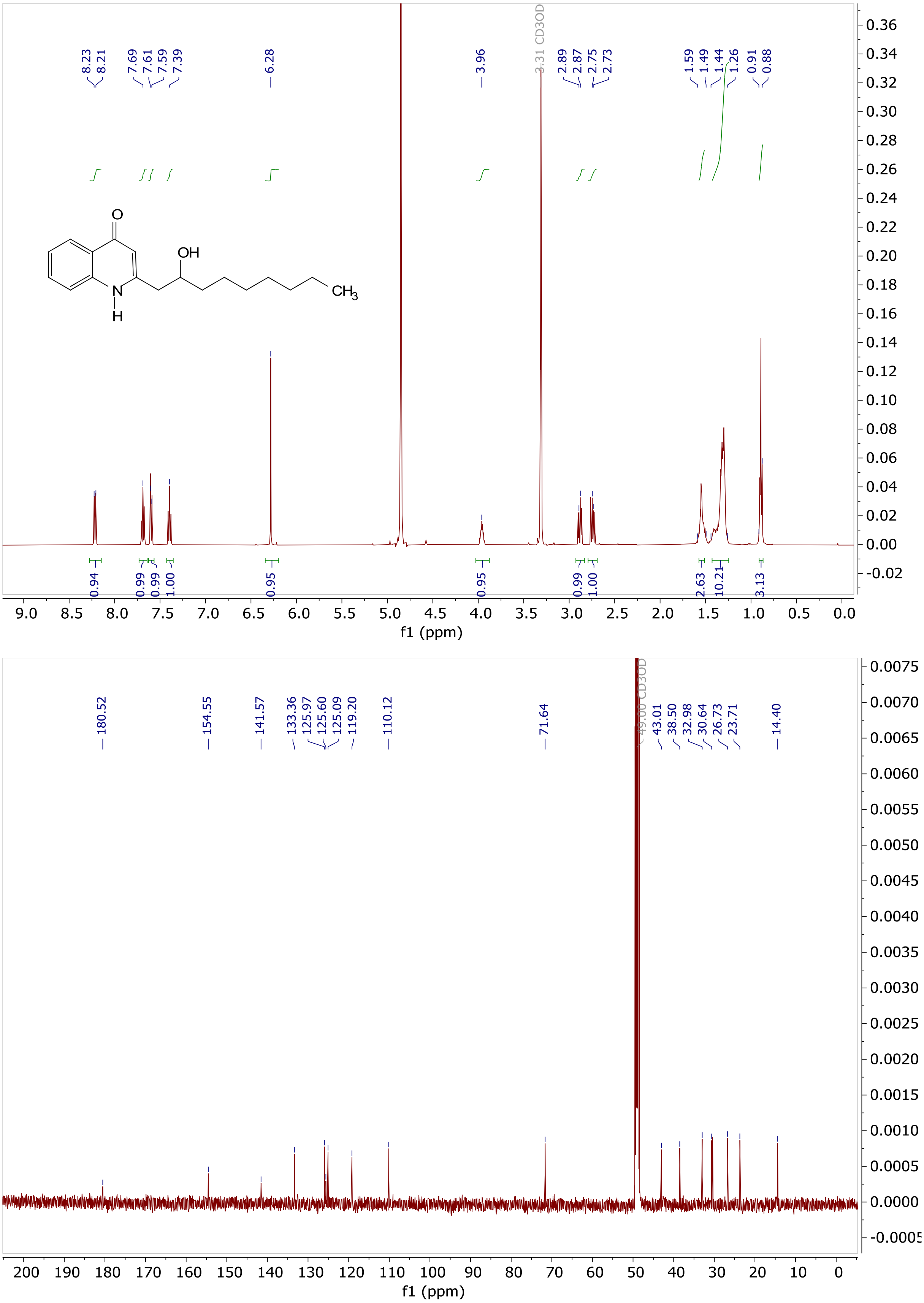

2-(2’-hydroxynonyl)quinolin-4(1*H*)-one (2’-OH-NQ) (**1g**) ^{1}H-NMR (above) and ^{13}C-NMR (below) in MeOH-d$_4$

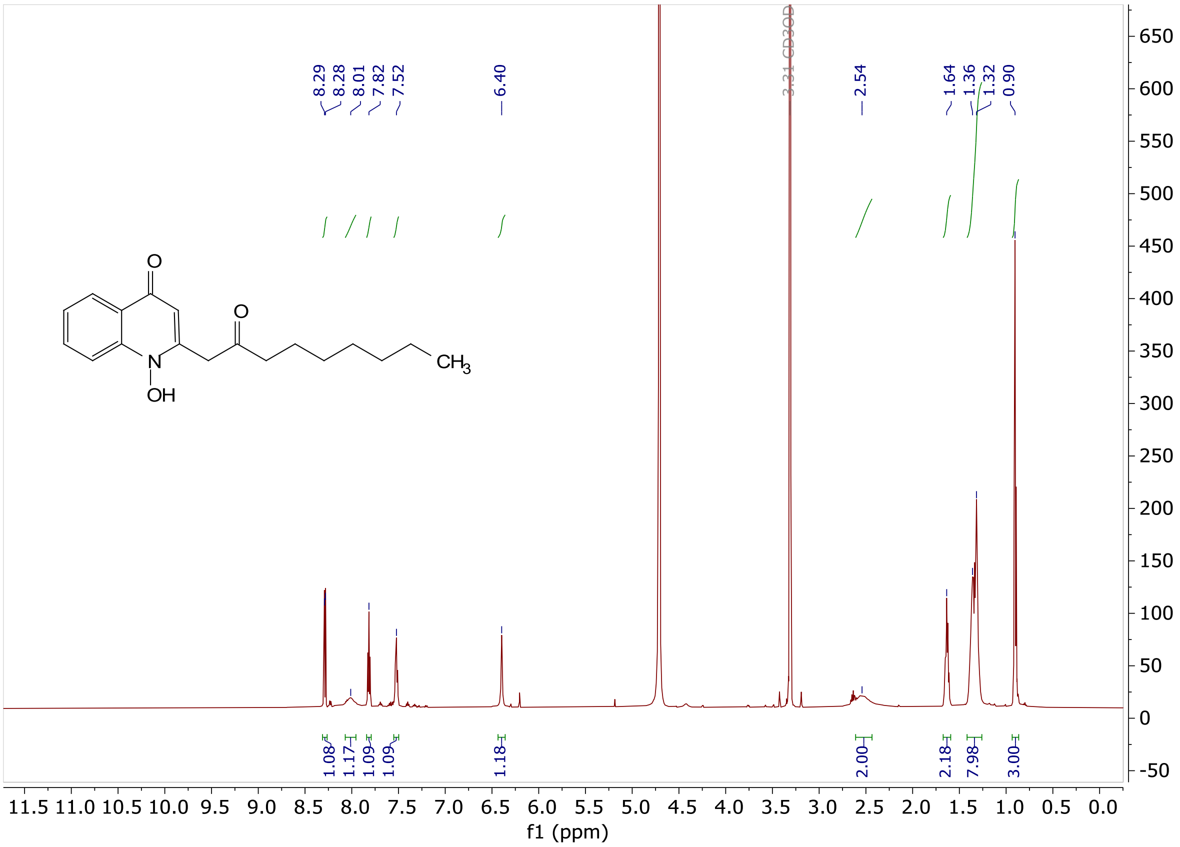

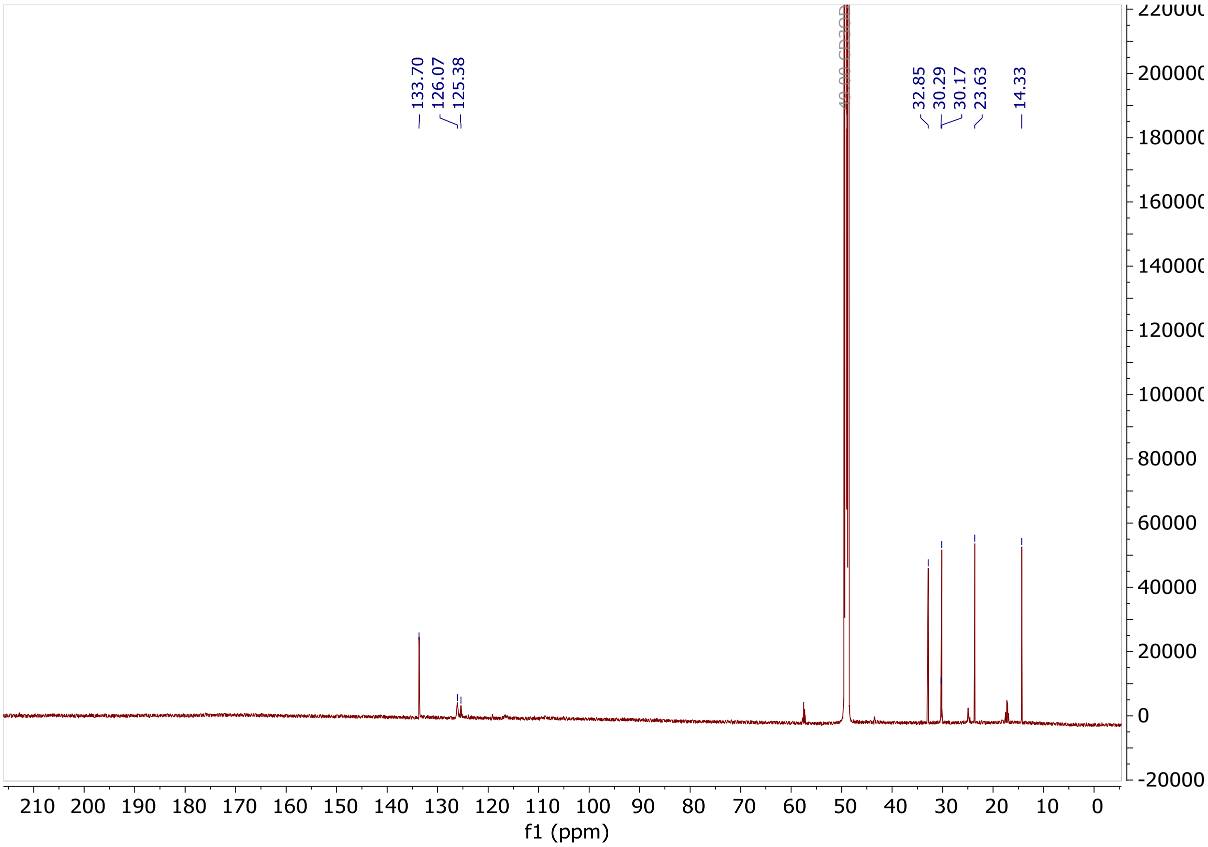

1-hydroxy-2-(2’-oxononyl)quinolin-4(1*H*)-one (2’-oxo-NQNO) (**2h**) ^{1}H-NMR (above) and ^{13}C-NMR (below) in MeOH-d_4

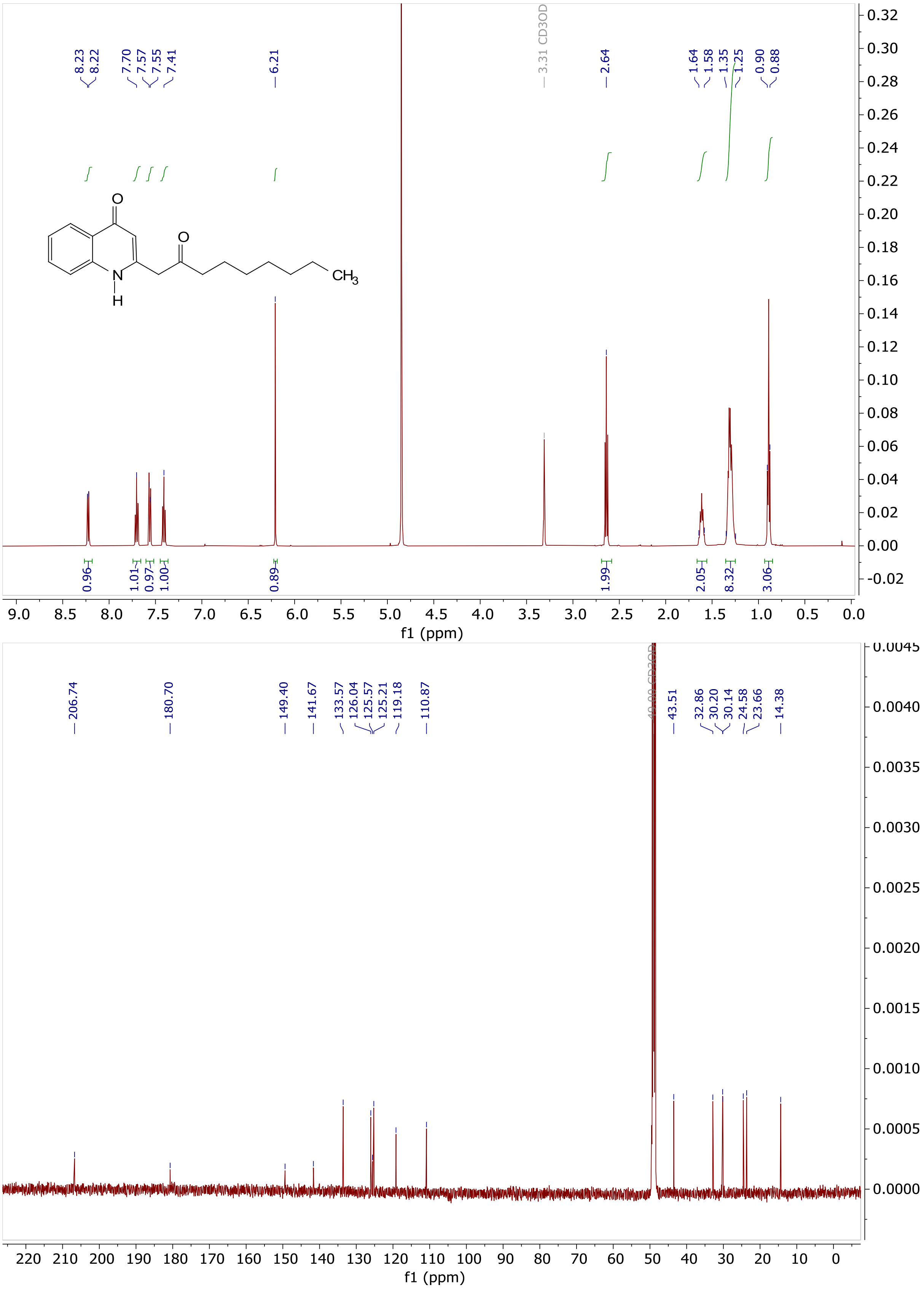

2-(2’-oxononyl)quinolin-4(1*H*)-one (2’-oxo-NQ) (**2g**) ^{1}H-NMR (above) and ^{13}C-NMR (below) in MeOH-d_4

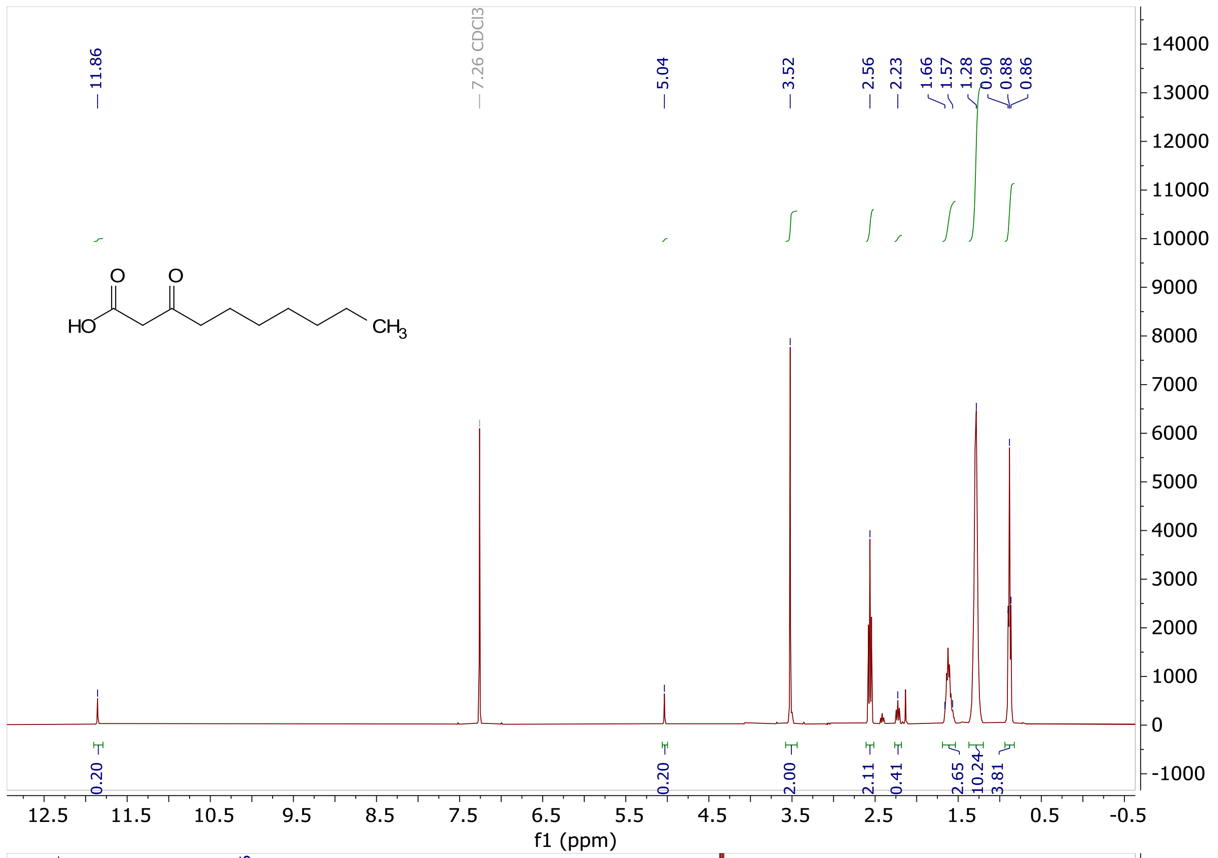

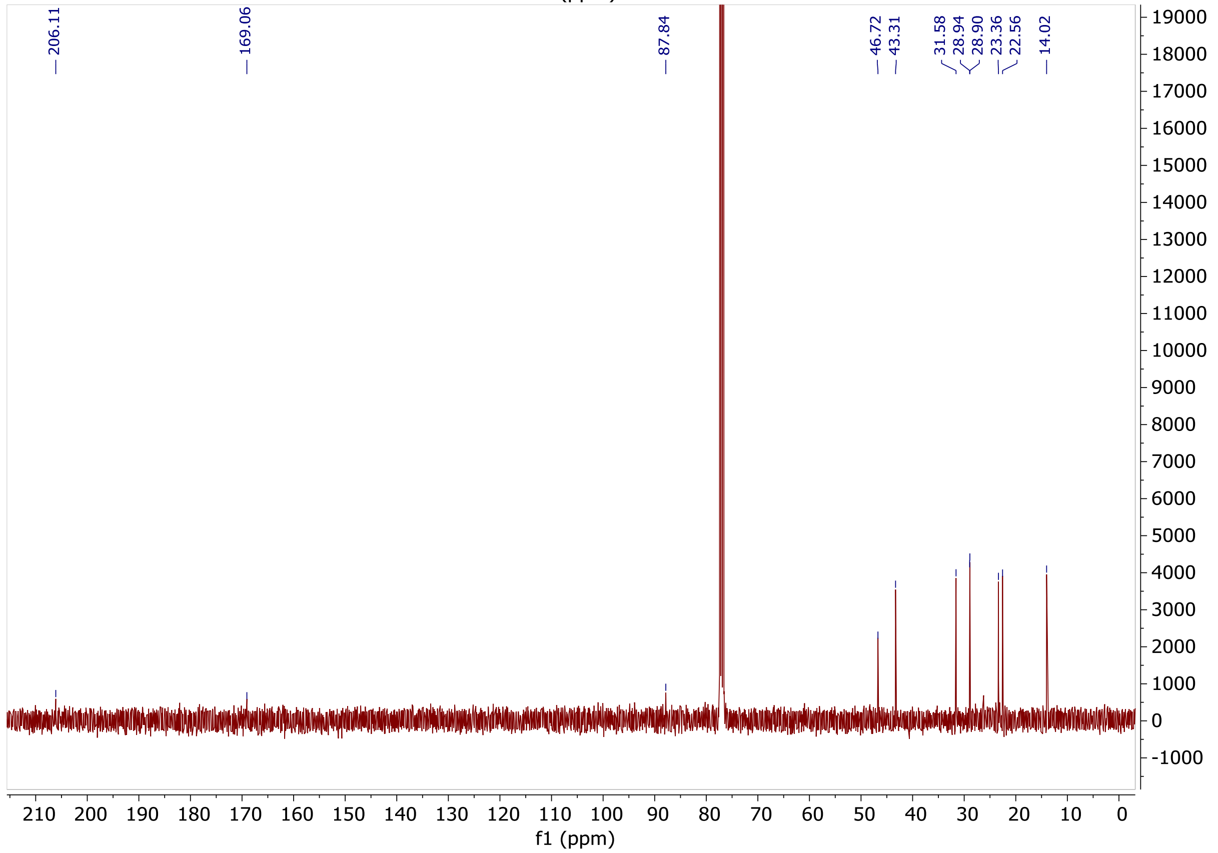

β-oxodecanoic acid (**3a**) ^{1}H-NMR (above) and ^{13}C-NMR (below) in $CDCl_3$

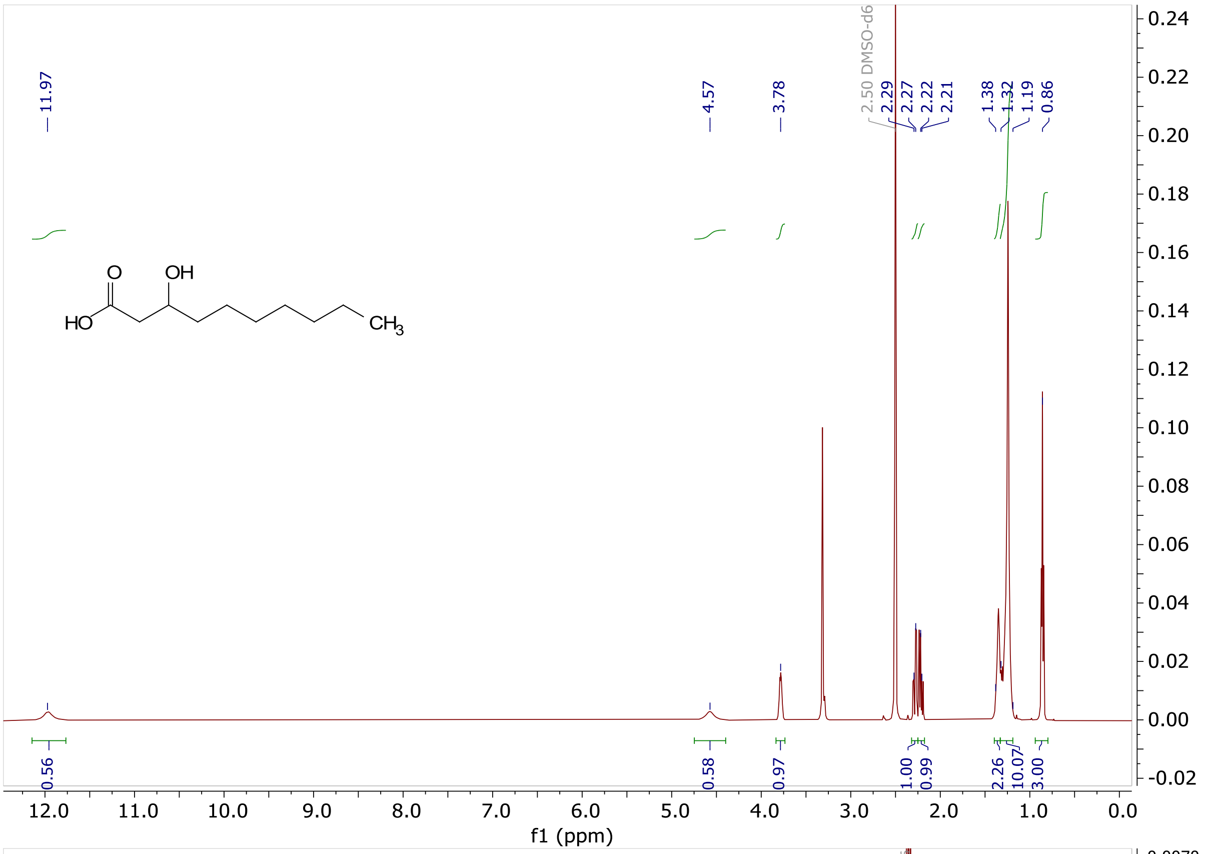

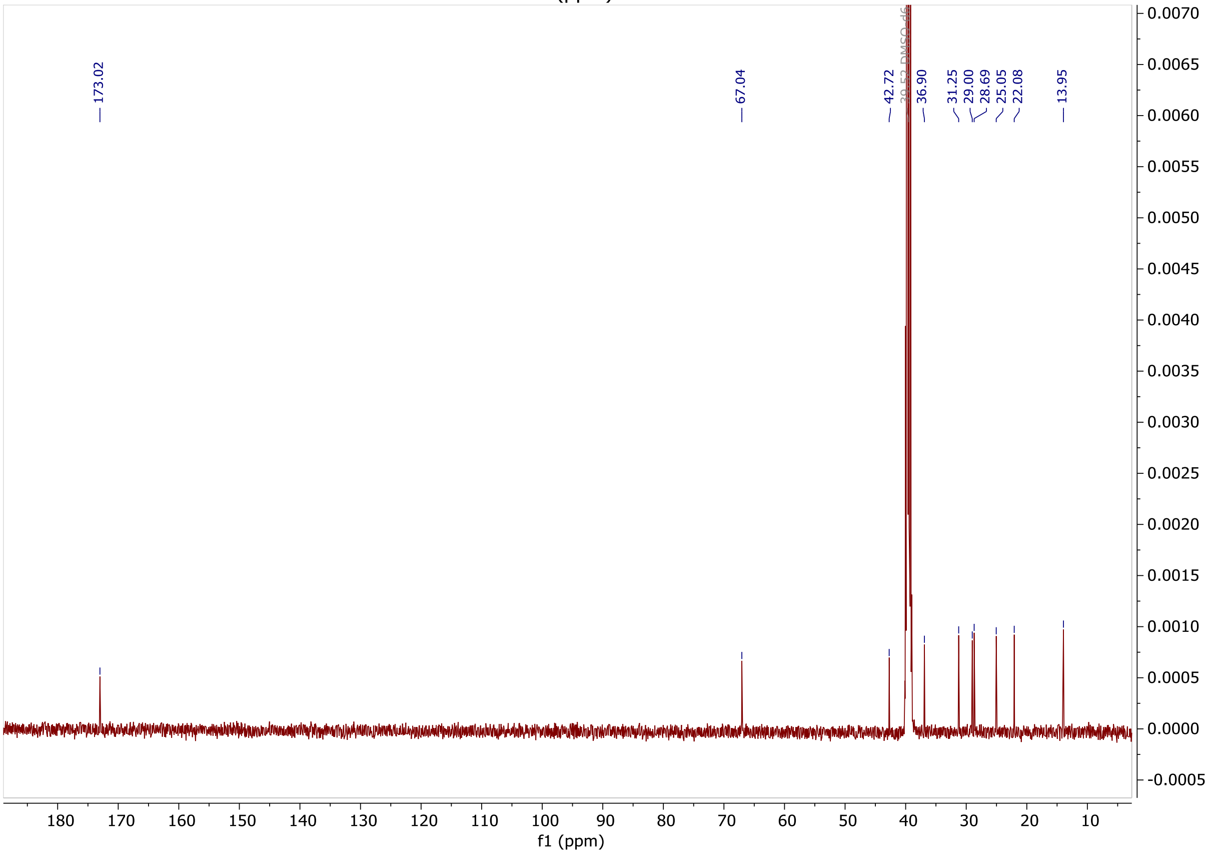

β-hydroxydecanoic acid (**3b**) ^{1}H-NMR (above) and ^{13}C-NMR (below) in DMSO-d$_6$

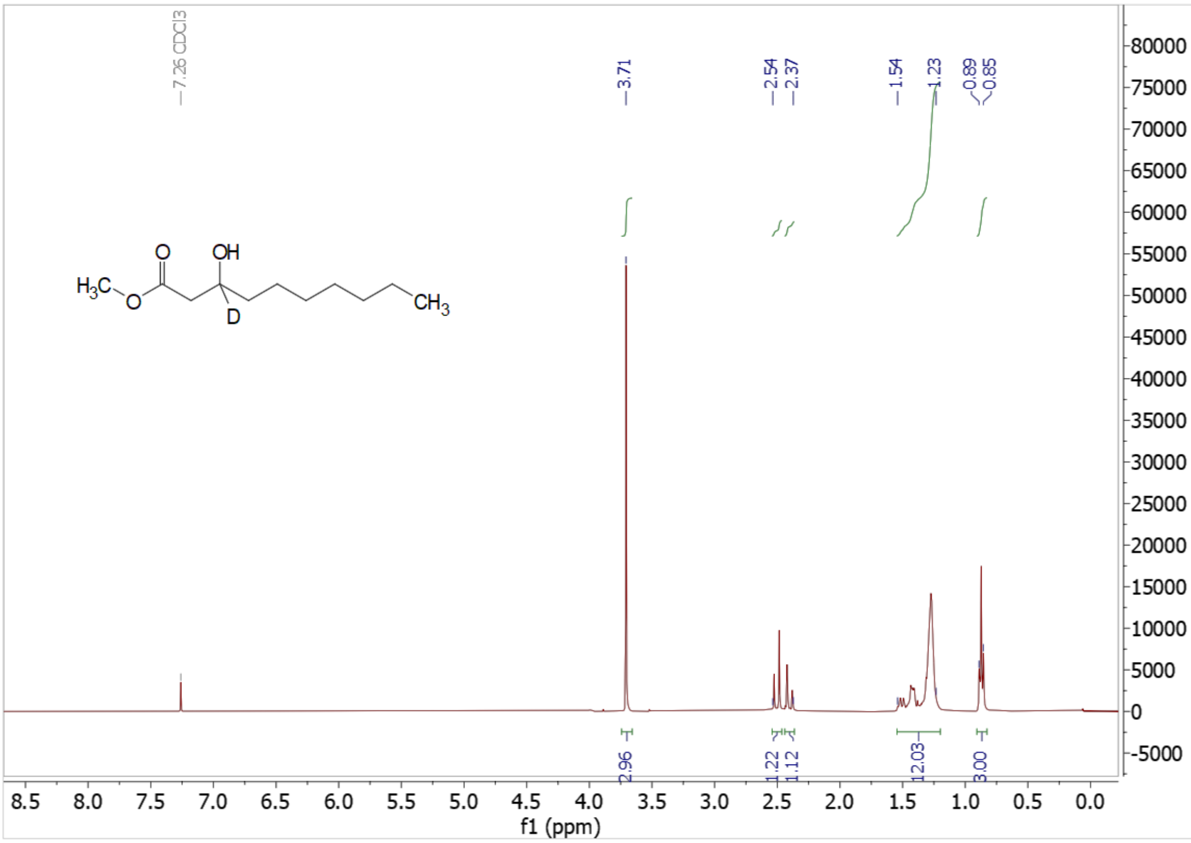

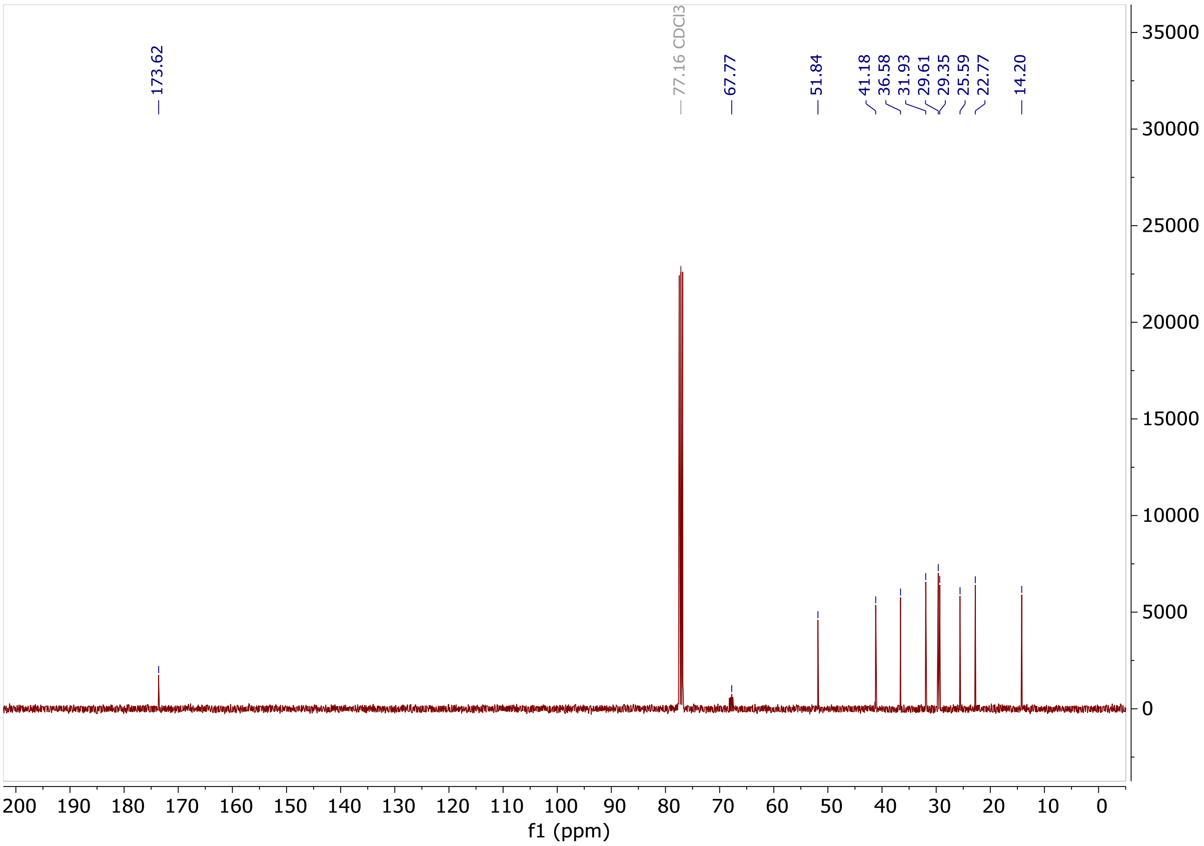

Methyl 3-hydroxydecanoate-d (**3c**) ^{1}H-NMR (above) and ^{13}C-NMR (below) in in $CDCl_3$

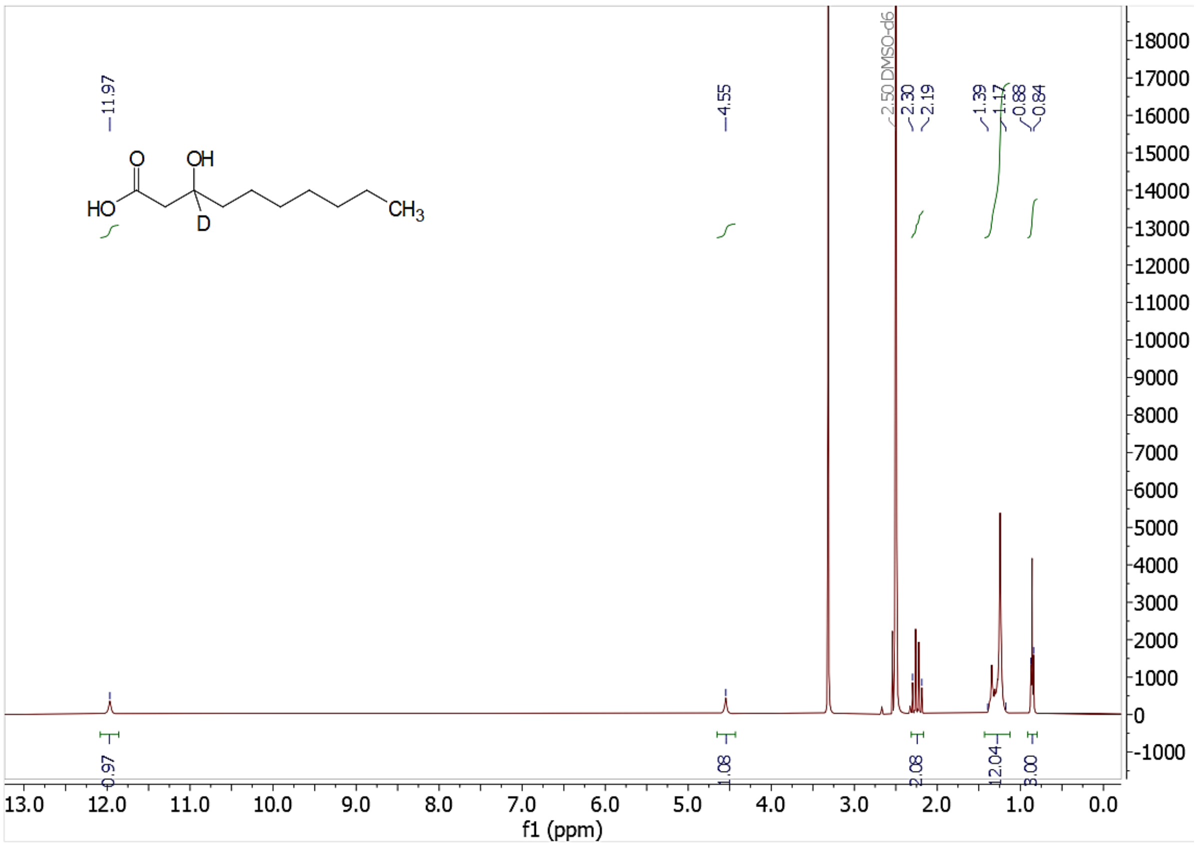

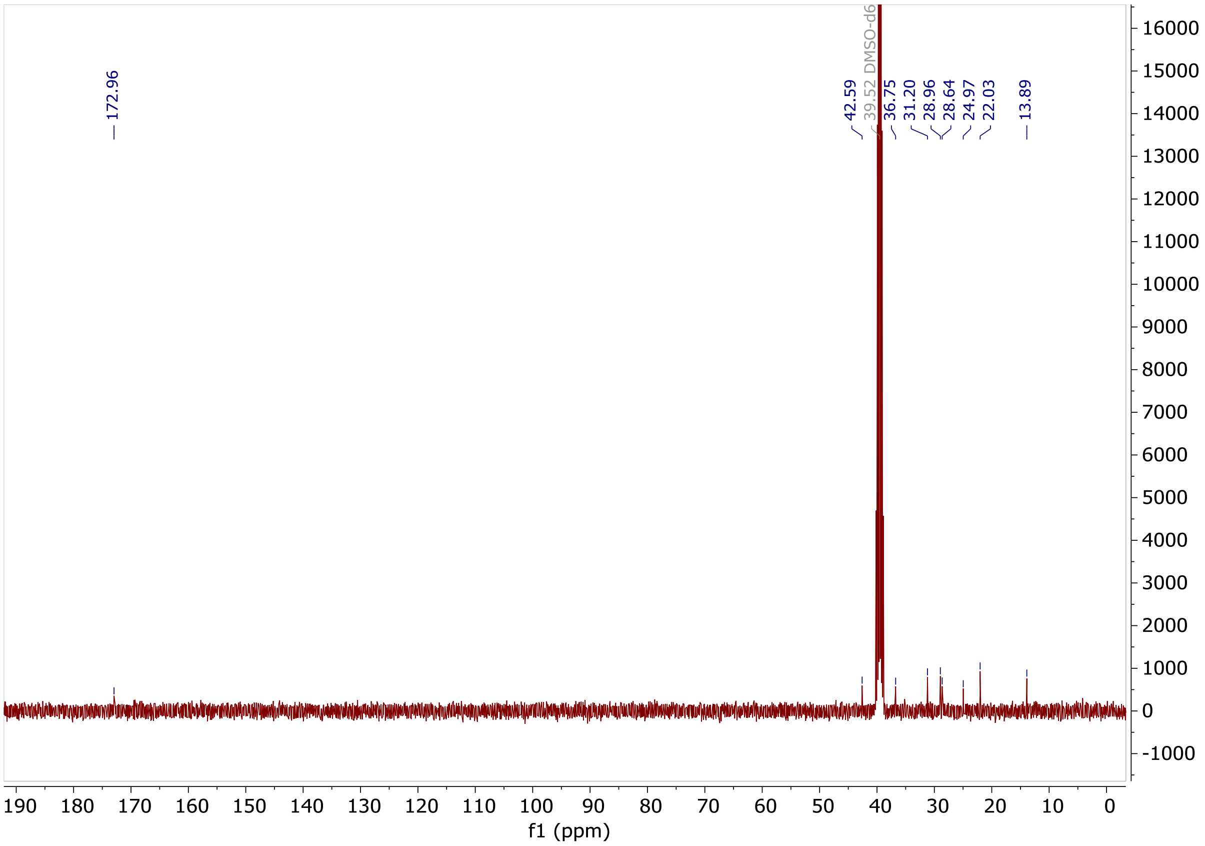

β-hydroxydecanoic acid-d (**3d**) ^{1}H-NMR (above) and ^{13}C-NMR (below) in DMSO-d_6
